# Automated monitoring of insects system: building a non-lethal and scalable solution for monitoring of nocturnal insects

**DOI:** 10.1016/j.ohx.2026.e00822

**Published:** 2026-08-06

**Authors:** William Lord, Simon Teagle, Joshua Alton, Gabriel Bannister, Jonas Beuchert, Kim Bjerge, Dylan Carbone, Alba Gomez Segura, Tim Howson, Toke Thomas Høye, Jenna Lawson, Abhi Ravivarma, David B. Roy, Dan Rylett, Grace Skinner, Alan Warwick, Tom August

**Affiliations:** a UKCEH, Wallingford, UK; b Department of Electrical and Computer Engineering, Aarhus University, Denmark; c Department of Ecoscience, Aarhus University, Denmark

**Keywords:** Insects, Monitoring, Nocturnal, Autonomous, Camera trap

## Abstract

There is growing evidence that human-induced climate change and habitat loss are having negative impacts on insect populations. New technologies have a vital role in improving and expanding global biodiversity monitoring capacity to understand where change is happening and to support restoration.

Monitoring of insects traditionally needs entomologists in the field, but insect camera traps powered by AI are emerging as a scalable approach to monitoring semi-autonomously. These systems attract, detect, and identify insects using a Raspberry Pi, camera and UV lights. AI algorithms are also being developed by a network of researchers across the world to help with identification, notably in Europe and North America.

The first version of a system for monitoring nocturnal insects was developed by Bjerge et al. 2021. An Automated Light Trap to Monitor Moths (Lepidoptera) Using Computer Vision-Based Tracking and Deep Learning. Here we describe the second generation of the system as an open-source solution. This paper aims to enable anyone to build their own system, and to iterate and improve the design for their needs. This system captures images at set intervals or based on motion detection to monitor insects that are attracted to lights at night. The UKCEH Automated Monitoring of Insects system (UKCEH AMI-system) is an insect camera trap designed using a single board computer, USB camera and attractant lights as the primary components along with peripheral accessories to make an autonomous system capable of long-term deployment in the field. Nearly 200 UKCEH AMI-systems have been deployed to date in over 30 countries around the world.

## Graphical abstract

**Figure:**
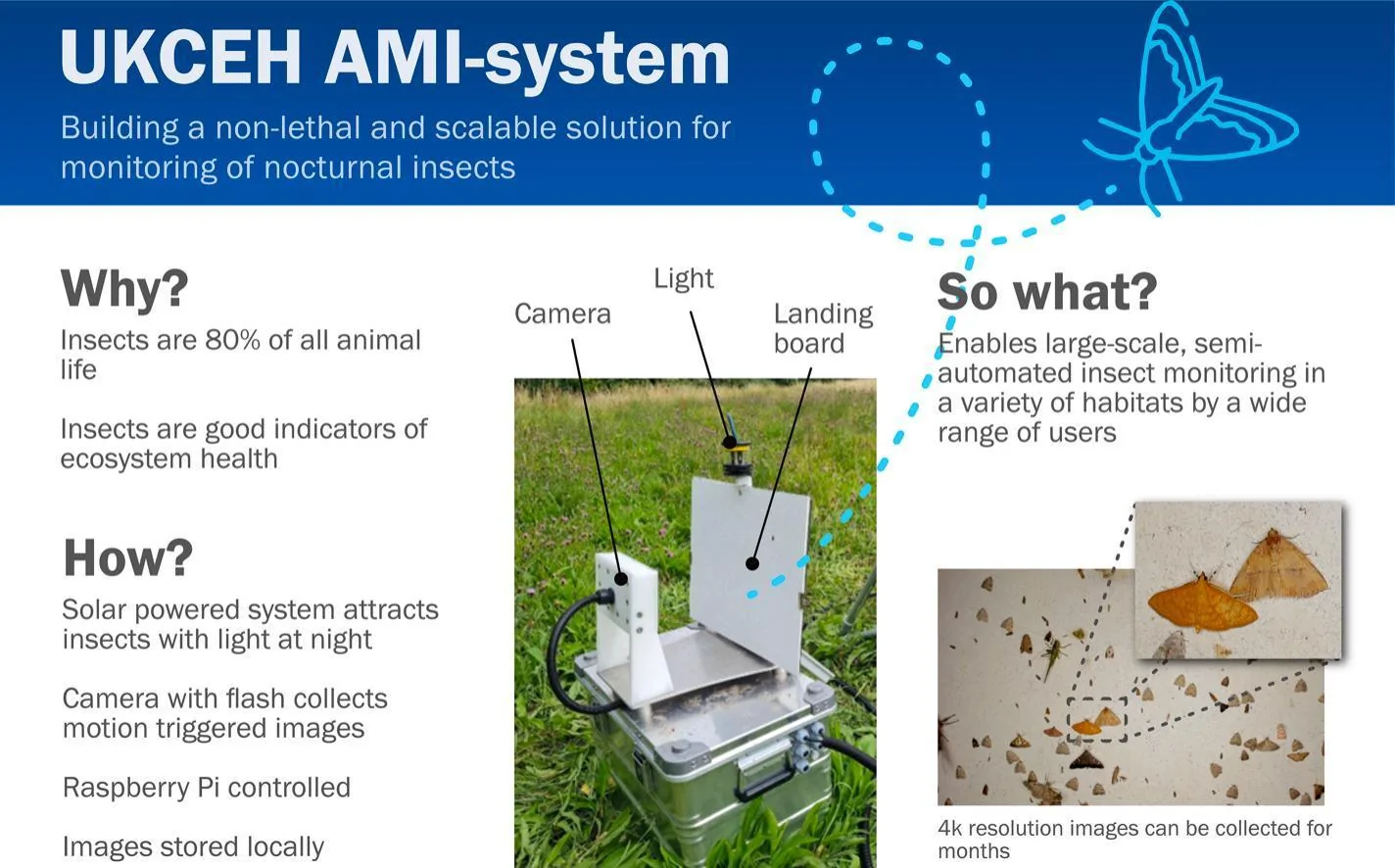

## 1 Hardware in context

There is growing evidence of widespread declines in insect diversity and abundance [1], caused by a range of environmental pressures such as habitat loss and climate change [2]. Robust monitoring data for insects is a priority for action [3], and necessary to identify solutions that lead to nature conservation, alongside economic prosperity. Insects are a critical component of biodiversity, playing a vital role in all terrestrial ecosystems, comprising approximately 80% of animal life [4] and providing critical ecosystem services such as pollination, pest control, nutrient cycling, and cultural services. The huge diversity of insects and their rapid response to environmental conditions also makes them highly effective as indicators of wider biodiversity condition. Nocturnal insects are a particularly attractive group to monitor since globally more insects are active during the night than during the day [5], and many taxa can be attracted to artificial light at night making them relatively easy to observe [6].

The solution we have developed, and field tested, is the UKCEH Automated Monitoring of Insects system (UKCEH AMI-system, Table 1). This builds on a system for monitoring nocturnal insects first published by Bjerge et al. [7]. We focus on a range of insects attracted to light (i.e. moths, and a range of other species groups), but note that spiders, molluscs and other invertebrates have been recorded by the traps as well. The system uses a light and camera to capture images. These images can then be classified using artificial intelligence (AI) algorithms and manually verified with entomologists. Platforms such as ANTENNA [8] and MothBot [9] allow users of the UKCEH AMI-system, and analogous devices, to run AI algorithms on their images and perform expert annotation. The system is deployed either powered by mains/grid electricity using a 12 V DC adapter or through a solar powered charging setup of 12 V batteries.

**Table 1:** Overview of the UKCEH AMI-system.

**Hardware name**	UKCEH Automated Monitoring of Insects system (UKCEH AMI-system)
**Subject area**	Environmental, Planetary and Agricultural Sciences
**Hardware type**	Field measurements and sensors
**Closest commercial analog**	Diopsis camera (https://diopsis.eu/en/camera/)
**Open source license**	CERN Open Hardware License Version 2 − Strongly Reciprocal
**Cost of hardware**	***Total cost of materials approximately – 2650 USD***
**Source file repository**	https://zenodo.org/records/18131879

The field of automated monitoring of insects with camera systems is relatively new, however, a number of systems, with both open and closed designs, are already available. Notably the commercially available Diopsis system is capable of monitoring nocturnal and day-flying insects [10]. Other systems include the EntoCam which uses design similar to traditional trail cameras [11], the MothBox which offers an open design [9], Lépinoc which uses a smartphone and bespoke app for capturing images [12], and others. In contrast to these systems, the UKCEH AMI-system aims to maximise the duration of deployment and weatherproof/ruggedness of the system, albeit at increased costs (Table2).

**Table 2:** A comparison of insect camera traps currently available.

Device name	Description	Cost (USD)	Camera resolution (megapixels)	Weight including batteries	Power options	URL
UKCEH AMI system	Rugged moth monitoring camera system trialed extensively around the world	2650	13 MP	60 kg	Battery, solar, mains	https://www.ceh.ac.uk/solutions/equipment/automated-monitoring-insects-trap
Diopsis	Commercially available day and night monitoring system	Not specified	8 MP	30 kg	Battery, solar, mains	https://www.faunabit.eu/en/products/diopsis
Moth Box	Rugged DIY moth monitoring camera system	393	64 MP	3 kg	Battery, solar, mains	https://mothbox.org/
Lépinoc	Citizen science moth monitoring camera system	Not specified	13 MP	2 kg	Battery	https://NOE.org/en/actions/lepinoc-pour-proteger-la-biodiversite-nocturne/
EntoCam	Compact and commercially available moth monitoring camera system	1395	64 MP	0.5 kg	Battery, solar, mains	https://outreachrobotics.com/drone-solutions/entocam/

There is currently a lack of standardised data on automated insect camera traps. Two parallel efforts are seeking to address this. First the WildLabs inventory is seeking to create a repository of all conservation technology including insect camera traps [13]. This inventory currently lacks many of the specific fields we would like to see for insect cameras, because it seeks to be a more general repository. Second is an initiative by the Insect AI COST action [14] to create a hardware database specifically for insect camera traps. This hardware database hopes to capture many details of greater relevance to these technologies, such as resolution in pixels per mm and external power options [15]. Both initiatives are still in early development. The data presented in Table 2 is in part informed by these two resources, as well as information from papers and websites, but is not an exhaustive list. Some specifications are hard to compare, as they can vary depending on how the user sets up the device, these include: run time on batteries, pixels per mm of the image, and the size of the area covered by an image. For an up-to-date review of insect camera traps we direct the reader to the WildLabs and InsectAI initiatives.

UKCEH AMI-systems, totaling 194, are now in use in over 32 countries across Asia, the Americas, Africa and Europe, supporting a range of projects. UKCEH AMI-systems have been deployed in arctic, temperate, and tropical systems, in open grassland and closed forest, and have endured tropical rainstorms as well as extreme heat. Users of the UKCEH AMI-system are diverse, and their questions similarly varied. This includes policy makers looking for tools to monitor the impact of policy decisions, businesses interested in evidencing their biodiversity action (e.g. habitat creation), researchers monitoring invasive species, farmers investigating sustainable farming practices, and financial institutions looking for methods to measure biodiversity credits.

## 2 Hardware description

The UKCEH AMI-system described here is a tool for researchers to study nocturnal insects attracted to light. With the capability of programmable intervals for autonomous data capture, weatherproof design and large storage capacity, the system can be deployed for long durations through the field season.

**Fig. 1:**
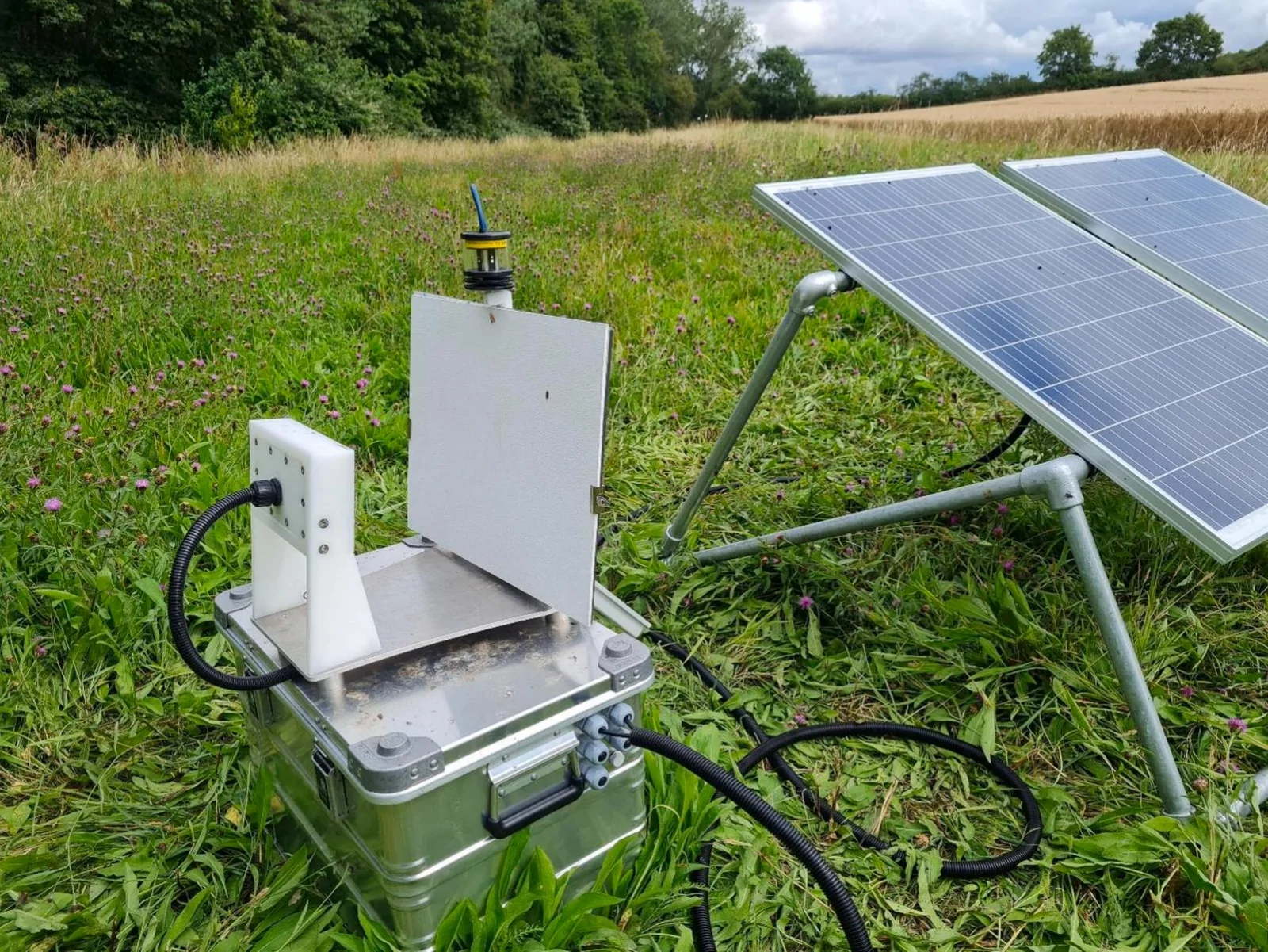
The UKCEH AMI-system consists of solar panels (right), and a battery box (bottom), on top of which is a camera housing (left), a white landing board (right) − where insects land − and an attractant light (on top of the backboard). The electronics enclosure is mounted to the back of the backboard (not visible).

Using experience from field deployments, we enhanced the original design of an insect camera system [7] to simplify manufacturing and assembly processes, improve operational usability, enhance image quality, and ensure overall system robustness. The current iteration of the AMI system enables deployment in remote locations and operation across a wide range of environmental conditions. Key improvements have focused on increasing resilience, allowing reliable performance in wet and high-humidity environments unlike the previous design, which required deployment under favorable weather conditions, manual start up and shutdown, and battery checks The updated system eliminates these constraints; the redesigned AMI system supports extended, autonomous operation, significantly reducing the need for frequent site visits while ensuring continuous and reliable data acquisition over prolonged periods (Fig. 1).

To achieve these improvements, we reduced the number of electronic components, ensured the system is protected against power interruption, can be turned off for periods of time to reduce power consumption, reinforced weatherproofing with an IP67 design, added solar charging capability for long term deployments in remote areas, and improved the LED light ring. Along with all these, we also paid attention to the environmental impact of materials used in construction, the commercial availability of sub-components and use a modular design to facilitate field-based repairs and maintenance, and future technical developments.

The majority of the components that make up the UKCEH AMI-system are contained within the electronics enclosure. All the electronics components used for the operation of the system are described below and listed in the Bill of Materials.

We chose a Raspberry Pi model 4B for a single board computer to maintain and control the system operation. This model was chosen to allow for the ability to interface a high-resolution USB 3.0 video camera with high bandwidth data storage capability, along with sufficient processing power to manage the load during increased levels of activity (rapid image count and data storage). This choice also allows for future developments using different cameras and exploring the application of AI models on the device for processing images. We recommend a minimum of 4 GB ram to operate the system and so while it may be possible to operate the system with other models of Raspberry Pi, we would expect some minor modifications to be required.

For the digital camera, we chose a Logitech BRIO 4 K web camera with USB 3.0 to capture images of insects. The camera provides high resolution images (4 K) and is compatible with the open source software “motion” [16] that enables easy camera configuration, automated operation using scripts, and motion detection. The “motion” software allows for extensive configuration which is documented in the project’s GitHub repository [16]. Images are saved to the device into day folders (YYYY_MM_DD when using a motion trigger YYYY_MM_DD-snapshot when using a set interval) and within this saved as.jpg files (YYYYMMDDhhmmss.jpg when using motion and YYYYMMDDhhmmss-snapshot.jpg when using a set interval).

We use a Super Watchdog Hat from Sequent Microsystems on the Raspberry Pi. Besides a watchdog timer, it provides an uninterrupted power supply (UPS) for only the Raspberry Pi, camera and SSD. It does not supply power to the lights or timer relay. In case of an unexpected power loss, it supplies power from a 2500 mAh18650 lithium-ion battery until pending tasks are completed and the system has safely shut down. This prevents corruption of data, i.e. the pictures being collected by the system.

To maintain the clock when the system is powered off, we use the Adafruit RTC DS3231, a real-time clock, with a CR1220 coin cell battery. This maintains the clock settings, crucial metadata information embedded into the images captured for analysis.

There are two data storage devices in the system, a micro-SD card in the Raspberry Pi, and a solid-state drive (SSD) attached to the Raspberry Pi for storing pictures. A Raspberry Pi OS image is flashed onto the micro-SD card of 64 GB capacity, this contains all the software that is operating the UKCEH AMI-system. The SSD is a high capacity and fast read/write capable storage utility used to store all the pictures captured by the system. This is also a USB 3.0 device that can be swapped during data retrieval to speed up data collection. We typically use a 500 GB SSD which can hold an entire season’s data with images collected every 60 s. Should a more intensive schedule be used, or a drive be allowed to accumulate many images over a long time period and become full, the device will stop saving new images.

The 12 V timer relay serves 2 functions, first to reduce power consumption and second to control the operating regime for the UKCEH AMI-system. By introducing the 12 V timer relay we are able to fully shutdown the system, meaning no power requirements during sleep intervals, with the timer relay much more energy efficient in these times. When scheduled to switch on, the relay boots up the system, this is useful for field deployments to operate the system autonomously during nocturnal periods. This also permits scheduling nights when the system does not run. We recommend not running the system every night, in an attempt to avoid negative impacts on local insect populations.

An LED light ring fitted around the camera housing supports illumination for the photography, these are a bespoke design made using 18 LEDs and 6 resistors mounted on to a PCB. The PCB construction is single sided FR4 (150 deg C) middle Tg, 1.2 mm thick with an immersion silver finish, size 170 mm x 90 mm. See the Zenodo repository [17] for the bill of materials and Gerber files.

To stabilise the 12 V supply to the LED lighting (preventing inconsistencies in brightness), we use a 12 V DC-DC regulator, which prevents fluctuations in the voltage supplied to the LED light ring for photography hence maintains uniform lighting of pictures.

For the attractant light, used to attract the insects towards the backboard, we chose the LepiLED [18] with a threaded bar, after iterations of designing our own UV light tube and evaluating other light options. LepiLED models are built using LEDs that emit UV, blue and green light in the electromagnetic spectrum (the three sensitivity peaks of most nocturnal insects) by eight Nichia Power LEDs contained in a weatherproof housing with an effective heat dissipation design. New models of attractant light are becoming available (e.g. EntoLED2 [19], entolight [20], MothBeam [21]) and the UKCEH AMI-system can work with any 12 V attractant light as long as there is sufficient battery capacity to operate it continuously. Light sources with a different voltage can be used in conjunction with a regulator.

The backboard is manufactured from an aluminium composite panel. This allows the electronics enclosure and LepiLED to be securely mounted to the backboard as well as providing support to the landing board, which is the surface on which insects land to be photographed.

The landing board is a 5.5 mm thick 480 mm x 360 mm weather and boil proof (WBP) plywood sheet which is painted with Sandtex white textured paint. The textured paint provides the insects with an easier surface to land on rather than the smooth surface of the backboard. In addition to this, the paint provides further protection from the weather and diffuses the light reflected off the surface, reducing glare. The landing board is secured to the backboard using bulldog clips to prevent it from moving. In our experience the landing board can become dirty over time as the result of animal excrement, mould growth or dirt, amongst other things. It has been our observation that these markings can interfere with the downstream AI processing of images, in particular with patches of dirt being mistakenly detected as insects. While this is not a critical issue, we recommend cleaning or repainting boards seasonally.

The base plate joins the backboard, camera housing and battery box and is manufactured from 6082 aluminium. This material has excellent corrosion resistance as well as the strength required to maintain rigidity. We used a water jet cutter to make this part as it ensures high accuracy and is cost-effective, though other methods could be used. The high accuracy also ensures the camera housing and back board are in the same position each time the system is assembled, reducing the chance of images becoming out of focus.

The camera housing has the BRIO camera and LED light ring secured inside. The main body of the camera housing and back cover are machined from white acetal which has the required properties for withstanding a wide variety of environmental conditions. The front cover is manufactured from 4 mm polycarbonate. The polycarbonate ensures protection to the electronics inside and will not degrade with UV exposure, whilst letting all the light from the LEDs through. The front and back of the camera housing is sealed with a 1.5 mm silicone gasket. This gasket provides the waterproof seal for the housing.

The battery box is a metal Zarges box (mfr. part no. 40564). It has a series of glands installed into the box to allow for the solar panels and the UKCEH AMI-system to be connected. Inside the box there is space for three batteries (though we often use two); we use 120 Ah AGM lead acid deep cycle / marine batteries in parallel (12 V), but other similar batteries will also work. The maximum size per battery is 328 mm x 175 mm x 237 mm (length x width x height), to ensure they fit within the box. The box also contains a solar regulator (Morningstar Sunsaver) where the batteries, solar panels and UKCEH AMI-system are connected. The solar regulator controls the charging and load to ensure the batteries are not over charged or deeply discharged, which can damage them.

The design is modifiable, and we would encourage future users to consider how the system might be improved. For example, alternative light ring and attractant lights could be considered so long as they can run on the current power system. The “motion” software works with wide range of cameras which could be trialed – though this would require a modification the camera enclosure to accommodate the shape of any new camera.

## 3 Design files summary

MothLEDBoard_v2.zip – This file contains all the CAD files needed for making the LED board that acts as the ring light around the camera. This illuminates the nocturnal insects to ensure good image quality and to some degree acts to attract insects to land on the illuminated board.

AMI_hardware_drawings.zip – This file contains PDF drawings of the components that need to be cut. This includes detailed information on materials, dimensions and the size and positioning of holes.

AMI_solidworks_assembly_and_parts.zip – This file contains all the original SolidWorks part files and the assembly file. These files allow future users to more easily make edits to the designs we created.

**Fig. 2:**
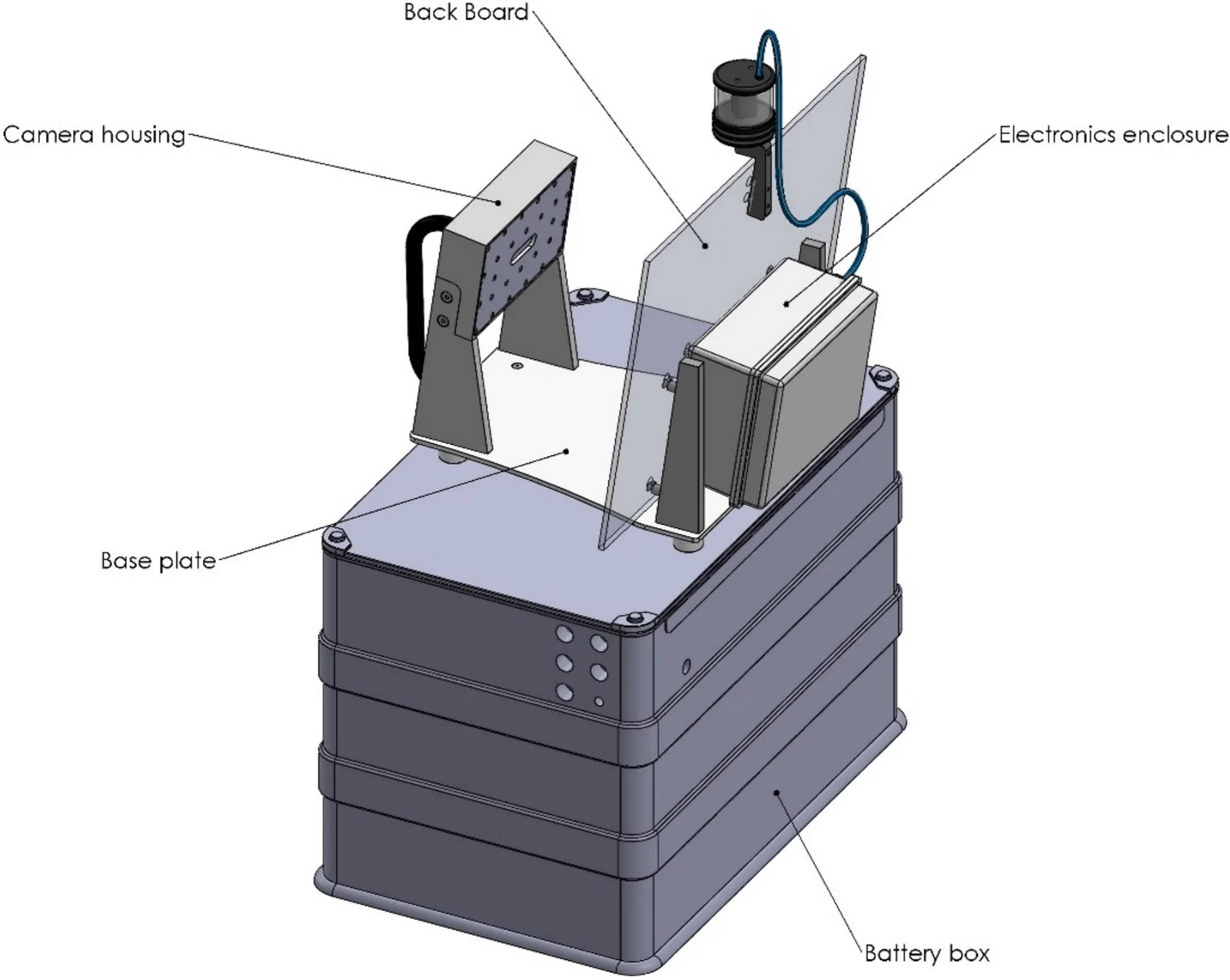
Overview of an AMI system on a battery box.

AMI_gasket_designs.zip – This file contains three DXF files, one for each of the gaskets that are used in the camera enclosure (Fig. 2).

AMI_overview diagrams.zip – This file contains PDF drawings showing how the various components of the UKCE AMI-system fit together.

In addition to the design files listed above (Table 3), the Zenodo repository also includes a detailed build manual, the bill of materials (BOM) and the image file for the Raspberry Pi [17].

**Table 3:** Showing the location and type of files required to build a system.

**Design filename**	**File type**	**Open source license**	**Location of the file**
MothLEDBoard_v2.zip LED ring light PCB design files	CAD files	CERN Open Hardware License Version 2 − Strongly Reciprocal	https://doi.org/10.5281/zenodo.20760744
AMI_hardware_drawings.zip Mechanical construction design files	Figures / drawings	CERN Open Hardware License Version 2 − Strongly Reciprocal	https://doi.org/10.5281/zenodo.20760744
AMI_solidworks_assembly_and_parts.zip Solidworks files	Solidworks assembly and parts	CERN Open Hardware License Version 2 − Strongly Reciprocal	https://doi.org/10.5281/zenodo.20760744
AMI_gasket_designs.zip Design files for three gaskets used in the assembley of the camera enclosure	DXF files	CERN Open Hardware License Version 2 − Strongly Reciprocal	https://doi.org/10.5281/zenodo.20760744
AMI_overview diagrams.zip	Figures / drawings	CERN Open Hardware License Version 2 − Strongly Reciprocal	https://doi.org/10.5281/zenodo.20760744

## 4 Bill of materials

The complete bill of materials is available with all other materials in our Zenodo repository for the UKCEH AMI-system, and is available here in the appendix [17].

## 5 Build instructions

The UKCEH AMI-system is broadly made up of five parts; the electronics enclosure, the battery box, the camera housing, the backboard, and the baseplate (Fig. 2). The system has some hazardous components and so the warnings in Table 4 should be placed on the system, and in instructions provided with the system.

**Table 4:** These warnings should be displayed in materials disseminated for the construction of UKCEH AMI-systems, and displayed on the device once constructed, to ensure safe operation.

	Warning: General warning, all users should be appropriately trained in: ● Manual handling ● Using required tools
	Warning: Fragile glass parts, handle with care to avoid breaking and then cutting with injury.
	Warning: Although all circuitry is considered low voltage, take care when dealing with electrical terminals and use correct/insulated tools where appropriate.
	Warning: Risk of electrical shock, ensure all power is switched off before any work on the electrical enclosure.
	Warning: UV light present. When system is powered on avoid eye exposure, avoid looking directly at light. Wear proper eye and skin protection.

### 5.1 Assembling the camera enclosure

For more detailed step by step instructions, see the build manual in the associated repository [17].

1. Mount the LED board (These can be manufactured using the files ‘MothLEDBoard_v2.zip’ and a PCB manufacture for example pcbtrain.co.uk) into the front of the LED light and web camera housing using the 4 x M3 standoffs and 4 x  M3 nylon screws (Fig. 3).

**Fig. 3:**
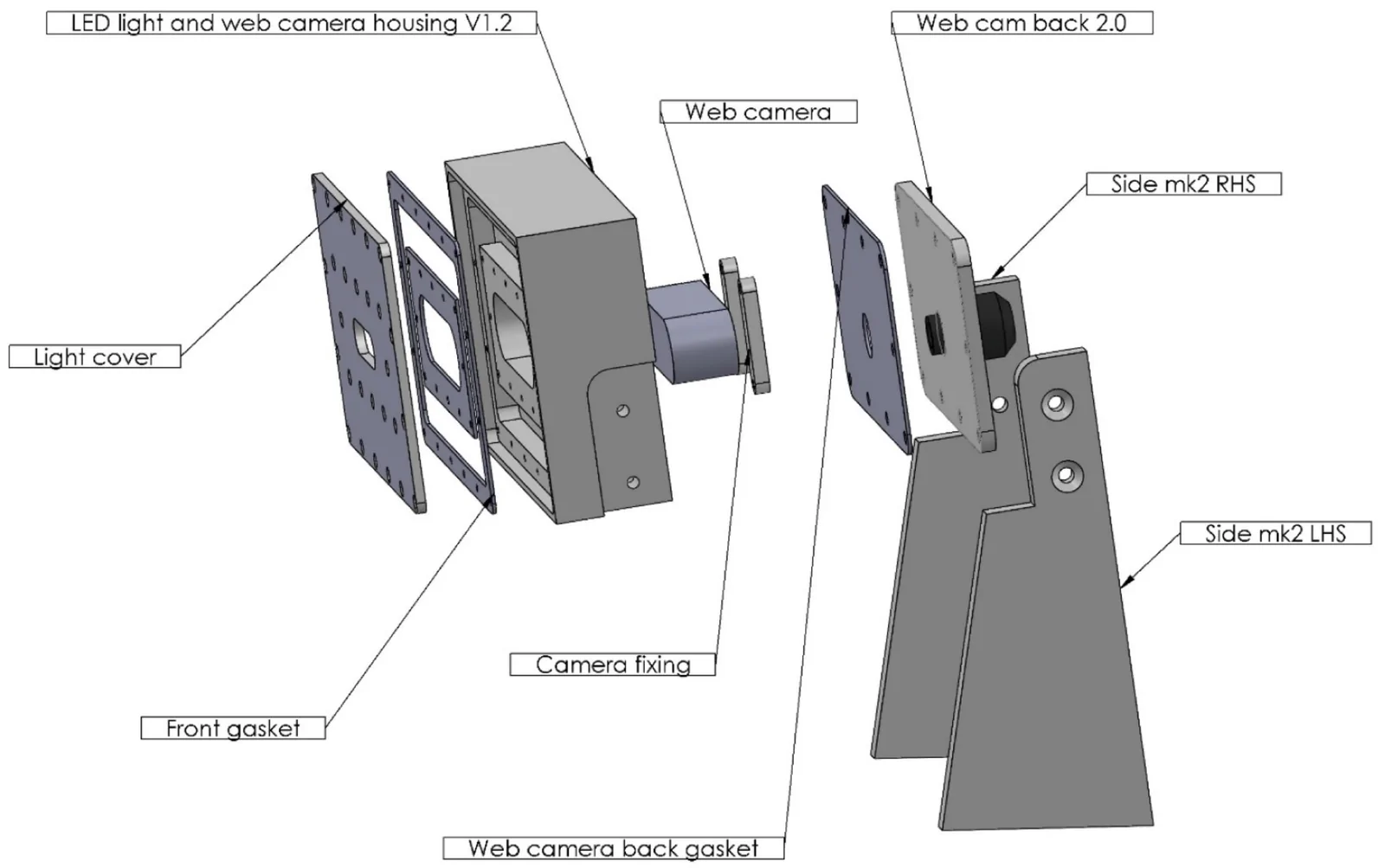
The assembly of the camera enclosure, including LED light ring, and the web camera.
2. Outer and inner gaskets are cut and punched by hand from laser cut stencil using the AMOS outer and inner gasket.dxf files.
3. Camera box is CNC milled.
4. Line the screw holes of the inner and outer gaskets with the holes on the housing and secure the LED cover using 28, M3 x 8 mm screws.
5. Turn the camera housing over and place the webcam into the slot and secure it with the 2 camera fixings.
6. Line the holes up in the back gasket with the holes in the web camera back and secure in place with the webcam back and 10, M5 x 12 mm screws.
7. Finally attach the left and right MK2 legs to the camera housing using 4, M6 x 20 mm screws.
8. Solder the two core cable to the positive and negative cable on the LED PCB board. Then pass the cable through the hole into the back of the camera housing. Next thread the two core cable and the USB cable from the camera through the cable gland and conduit.

### 5.2 Assembling the battery box

The battery box is adapted from a Zarges box 40,564 (410 x 600 x 400 mm).

1. Mark out the top of the battery box and drill the 5.3 mm diameter holes (Fig. 4). Use the battery box jig drawing for dimensions [17].

**Fig. 4:**
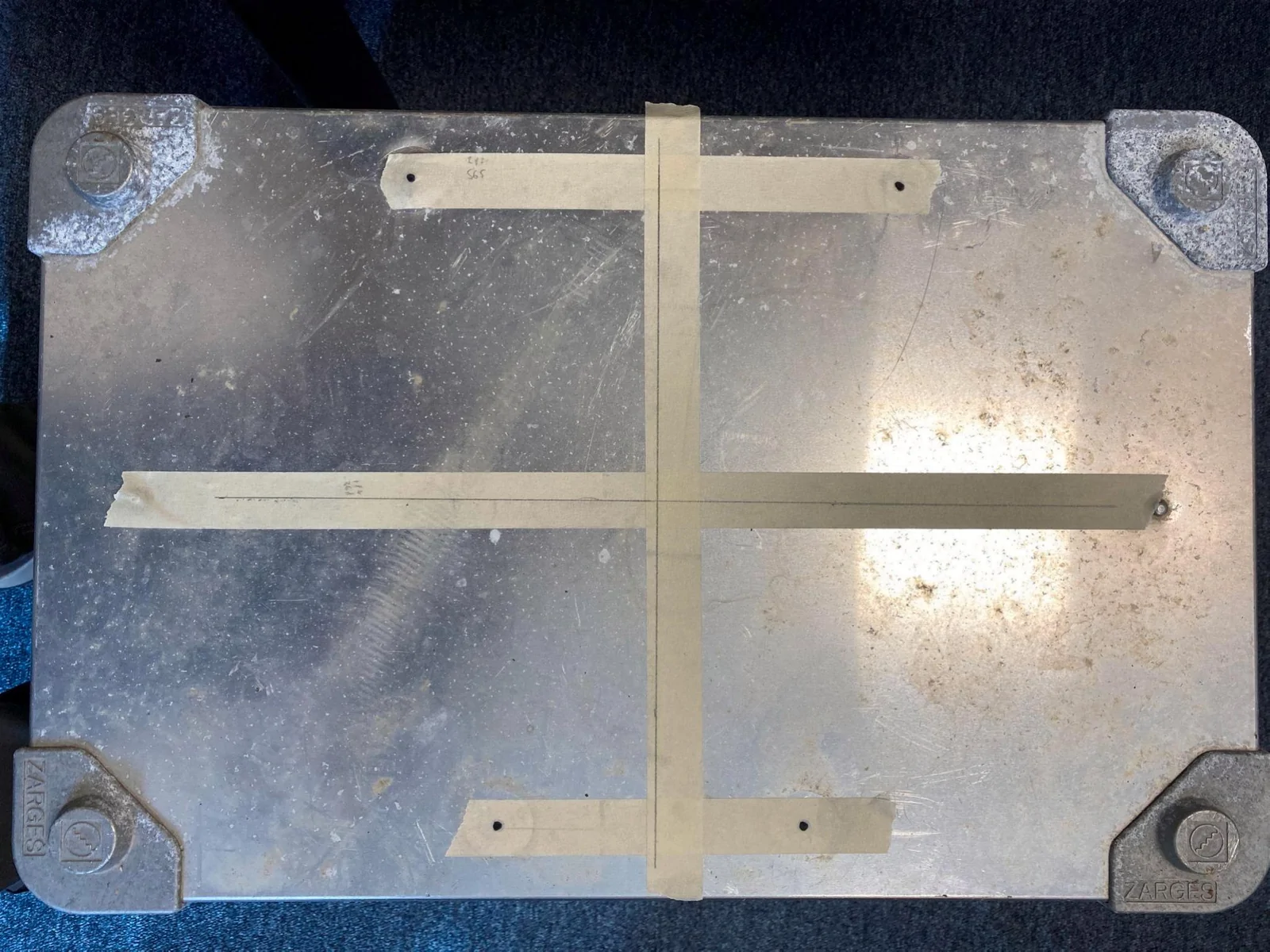
Holes drilled into the top of the battery box.
2. Mark out the hole position shown in Fig. 5 and drill a 16 mm hole in the back left of the box to allow a power supply cable that will run from the battery box to the electronics enclosure (Fig. 5).

**Fig. 5:**
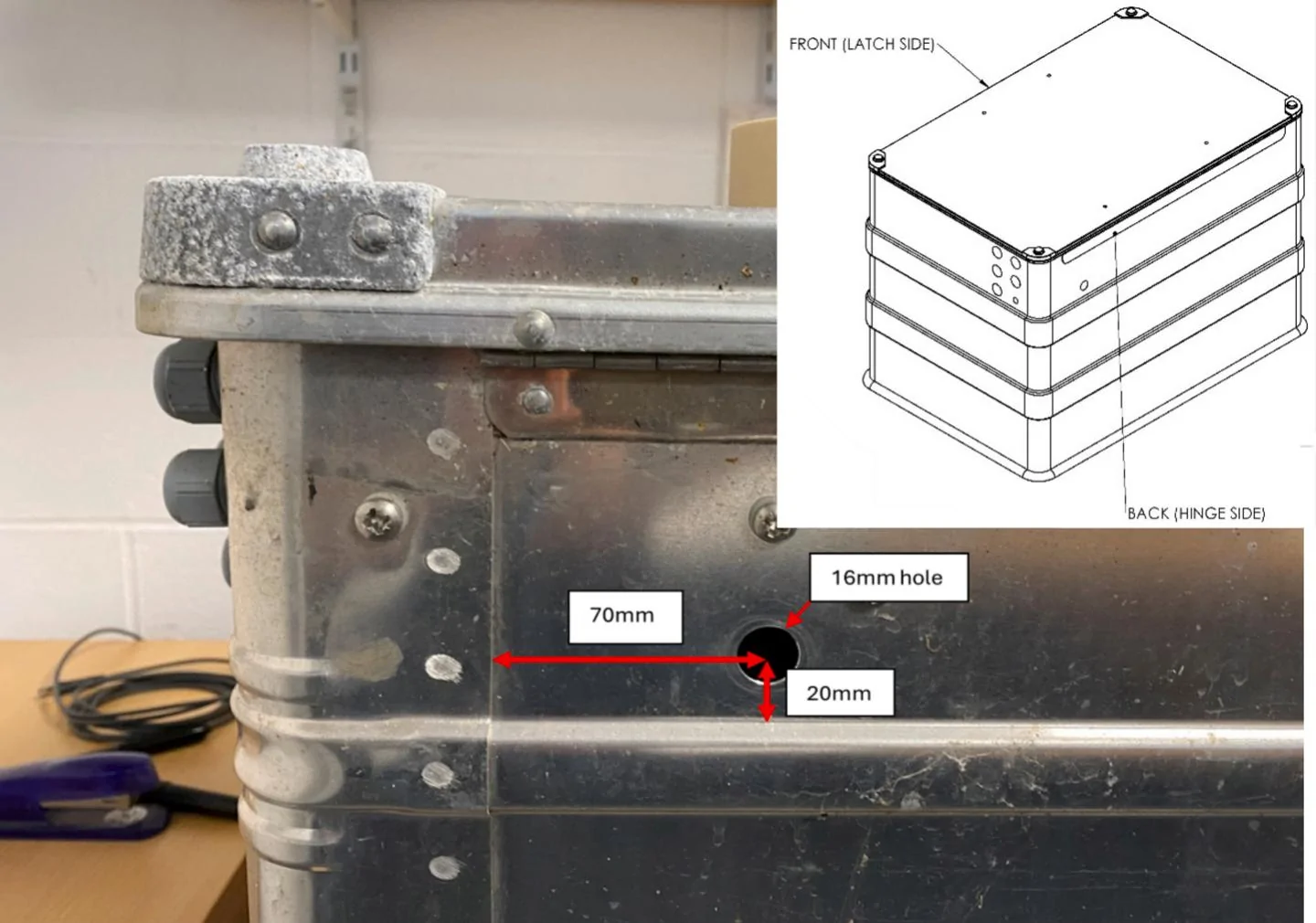
Marking up the hole on the back of the battery box.
3. Install a solar regulator to the back of the battery box close to the top to allow for cables to connect.
4. Install fuses to the back right of the battery box, in line with the top of the box to allow space for cables to connect.
5. Wire the solar regulator to fuses following the manufacturers instructions.
6. Pass the load wires from the fuses into conduit (300 mm long) securely attached to the hole made in step 2, ready to connect to the electronics enclosure.
7. For advice on batteries and solar panels see the section ‘Solar panel and battery recommendations’.

### 5.3 Assembling the electronics enclosure

**Fig. 6:**
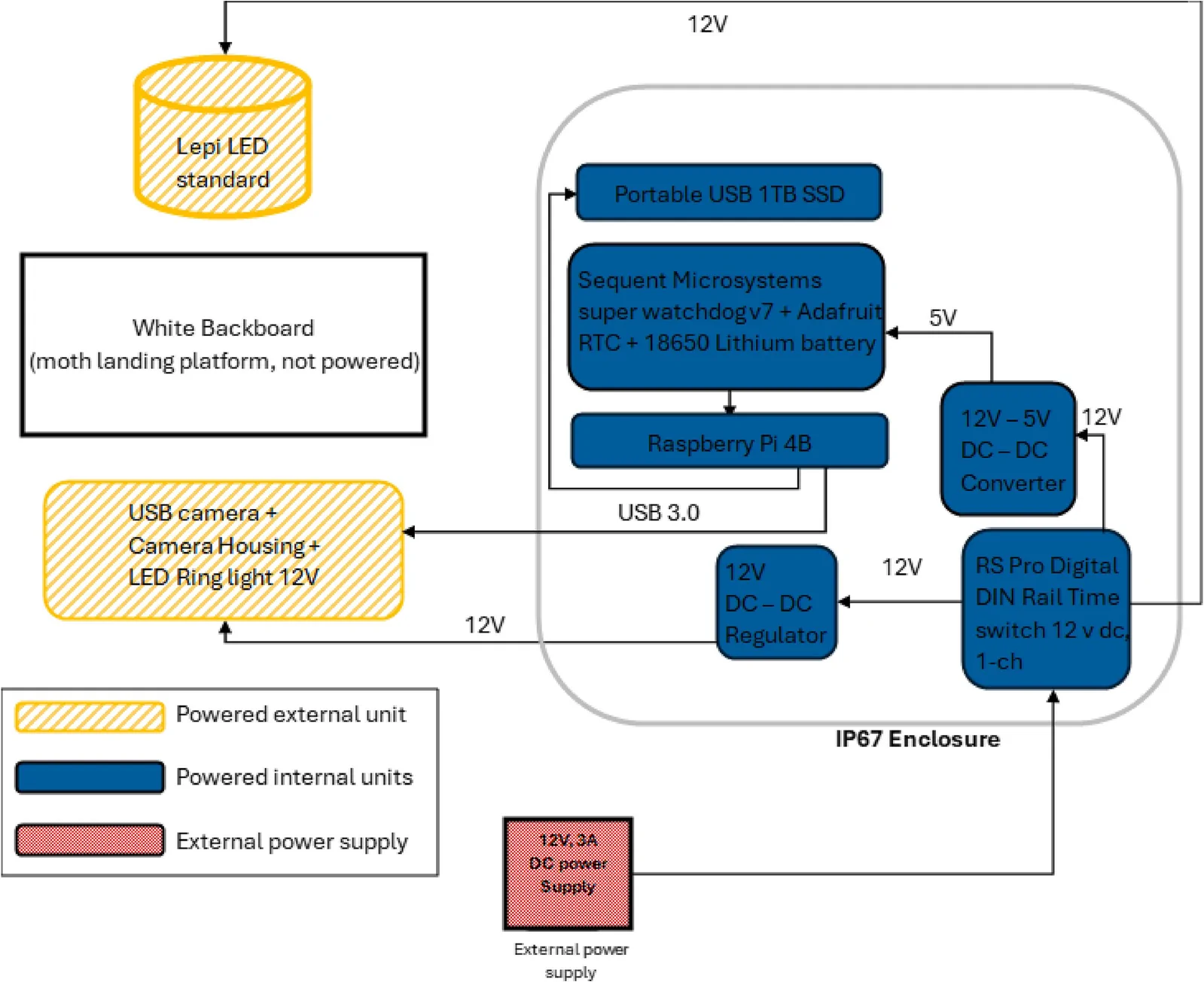
A schematic diagram for the electronics operating the UKCEH AMI-system, most of this is housed inside the electronics box (grey line).

The electronics enclosure is an IP67 enclosure that contains all the components that enable the central functionality through software operation and data capture. Fig. 6 gives an overview of the electronics operating the UKCEH AMI-system, much of which is housed in the electronics enclosure. For detailed step-by-step instructions on installing components into the electric’s enclosure see the build manual [17]. The procedure is outlined here in summary.

1. Install the DIN rail into the electronics enclosure and clip on the terminal blocks and the timer relay (Fig. 7).

**Fig. 7:**
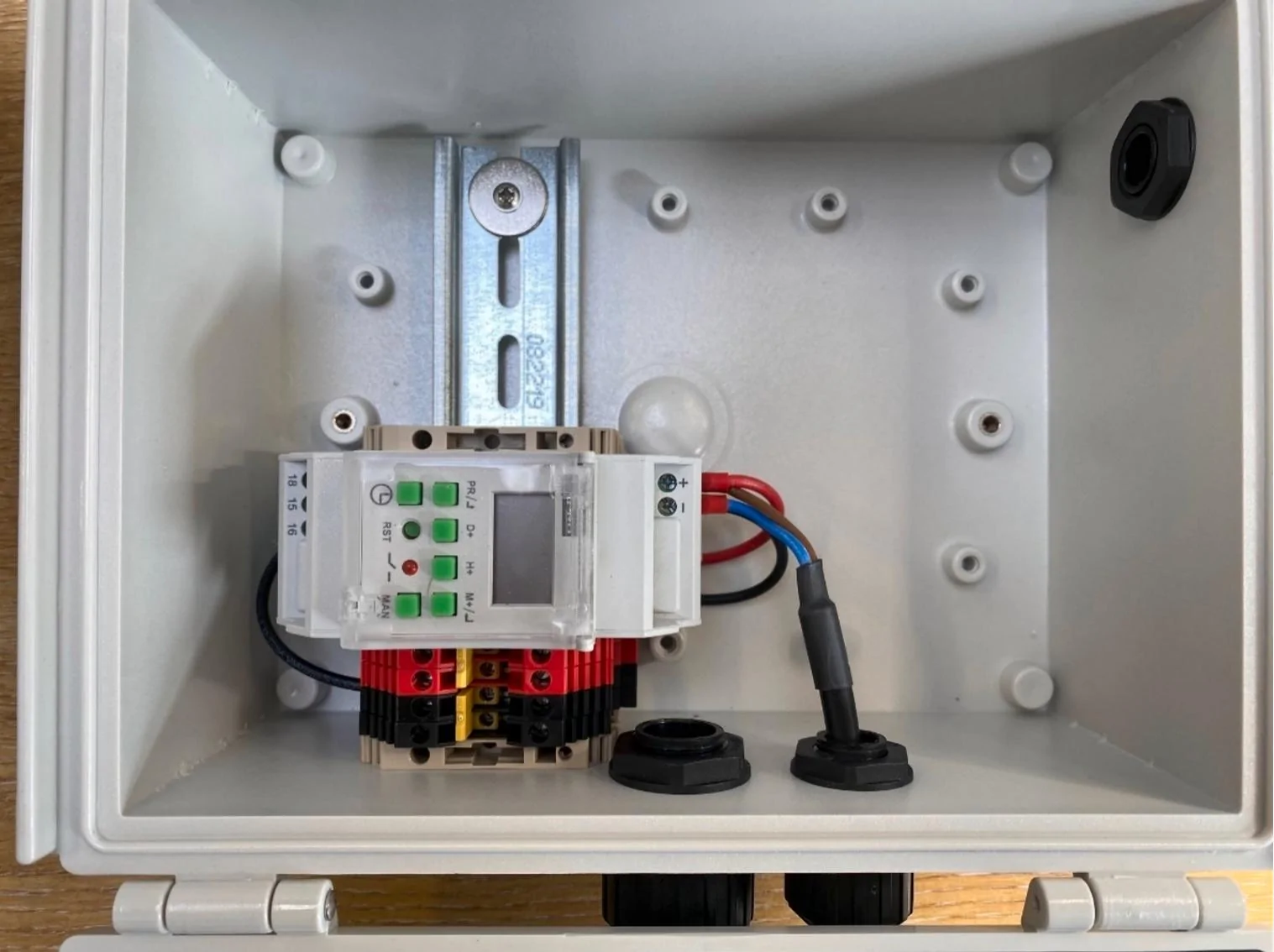
Terminal block and timer relay installed on DIN rail (wiring described in later steps).
2. Secure 12 V regulator mounting plate to the enclosure box using two polyfix 4 x 10 mm screws and mount the 12 V regulator with two number 4 self-tapping screws (Fig. 8).

**Fig. 8:**
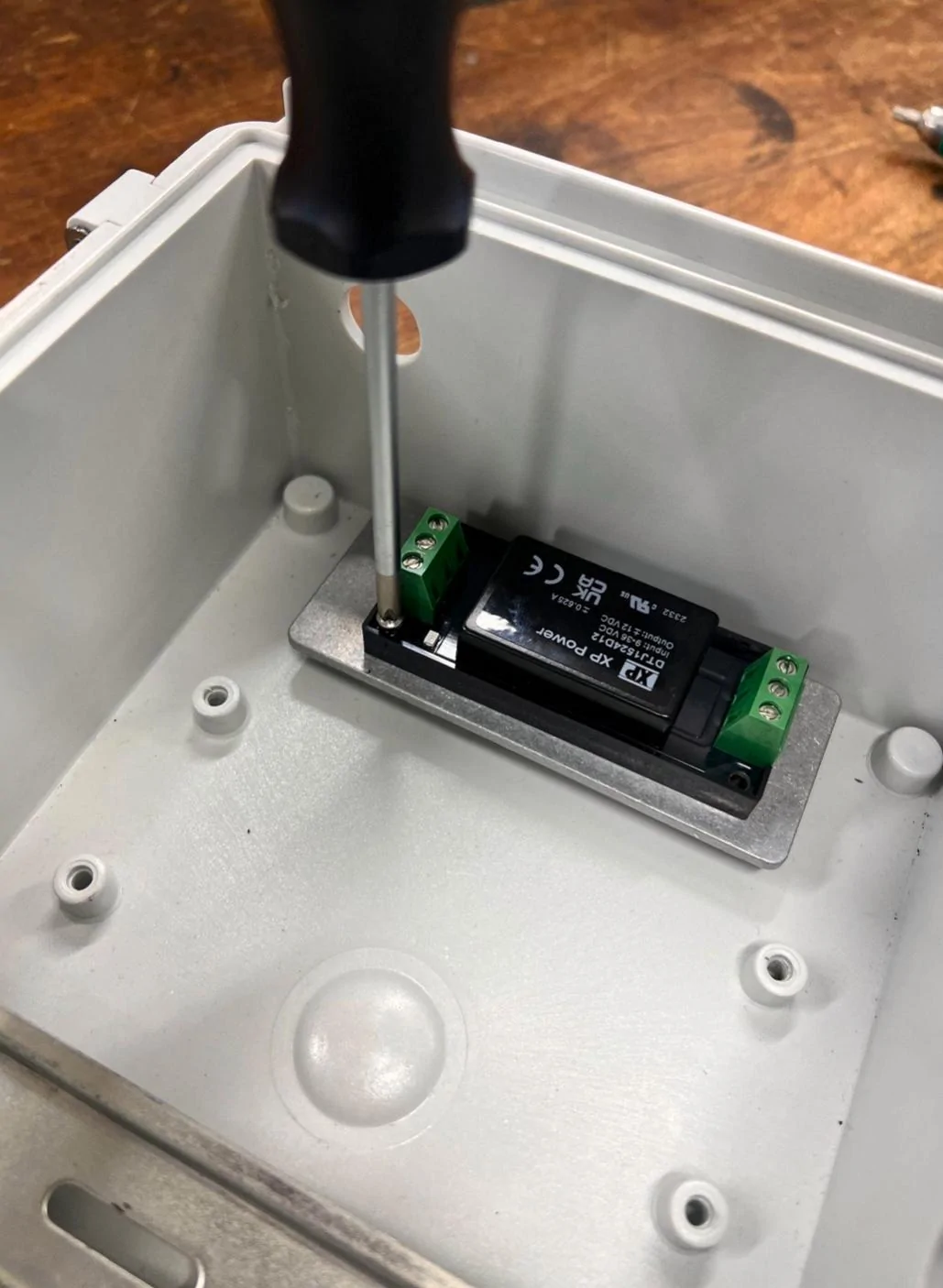
Mounting the 12 V regulator, see Fig. 17 for overview of electronics enclosure.
3. Mount the Raspberry Pi on top of the DINrPlate and then mount the watchdog on top of the Raspberry Pi on stand-offs (Fig. 9).

**Fig. 9:**
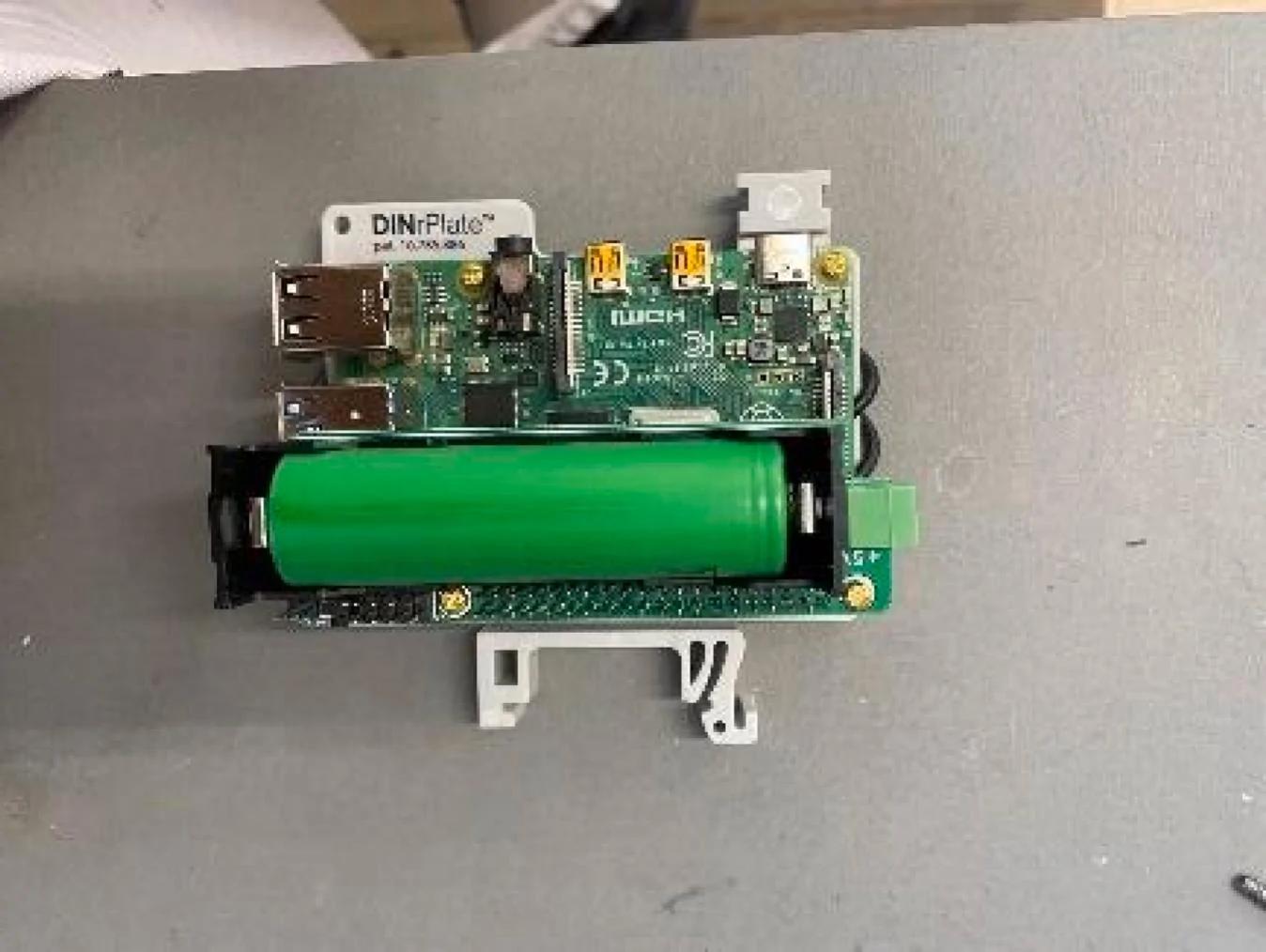
Raspberry Pi and Watchdog mounted on top of DINrPlate.
4. Mount the header extender on the RTC, and mount them onto the watchdog.
5. Make sure the battery (CR 1220) is inserted into the real time clock, this should last for 5 years with an accuracy of +/- 1 min per year.
6. Plug the watchdog into the 12 V to 5 V stepdown and mount the assemblage of Raspberry Pi, watchdog, RTC and DINrPlate onto the DIN rail above the timer relay (Fig. 10).

**Fig. 10:**
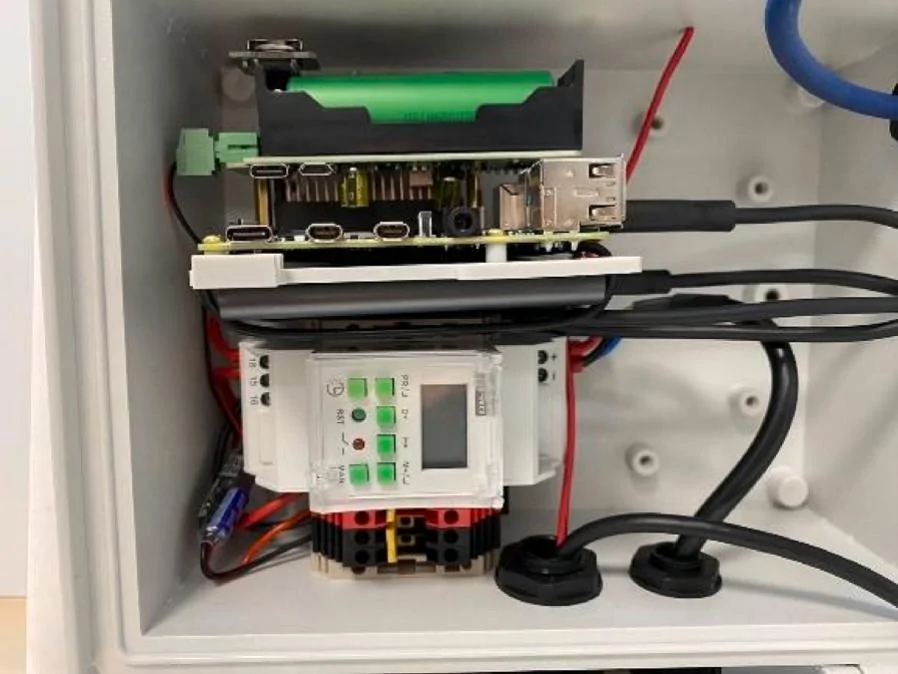
All components attached to the DIN rail (wiring described in later steps).
7. The camera and lighting cables come out of the end of the protective conduit from the back of the camera box and can be seen in Fig. 11. To thread and connect the cables into the electronics box unscrew and remove the thin black plastic conduit connector nut (step 1 in Fig. 11).

**Fig. 11:**
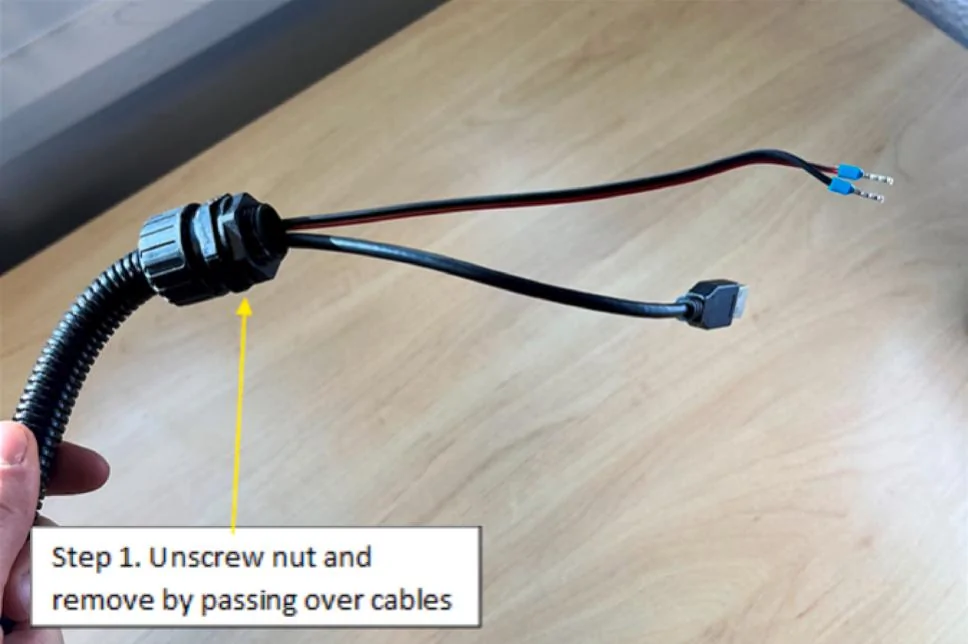
Conduit nut is first removed from cables extending from the back of the camera housing.
8. Thread the USB and power cables through the hole in the electronics box (step 2 in Fig. 12).

**Fig. 12:**
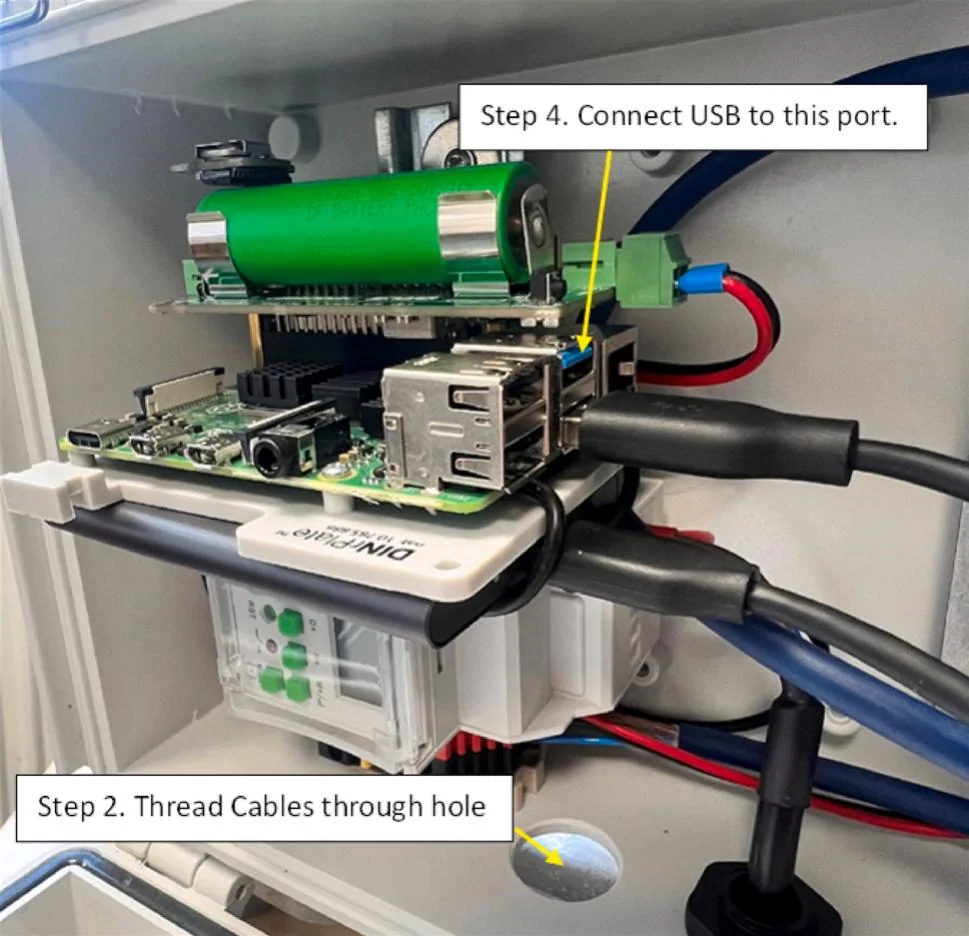
Wiring the camera and lighting ring into the electronics enclosure, steps 2 and 4. See Fig. 17 for overview of electronics enclosure.
9. Push the conduit and connector as far into the hole (step 2 in Fig. 12) as it will go, then replace the nut removed in step 1 (Fig. 11), passing it over the USB and power cables, then screwing it onto the conduit connector until tight, this should be slightly more than hand tight so use a spanner (step 3 in Fig. 13).

**Fig. 13:**
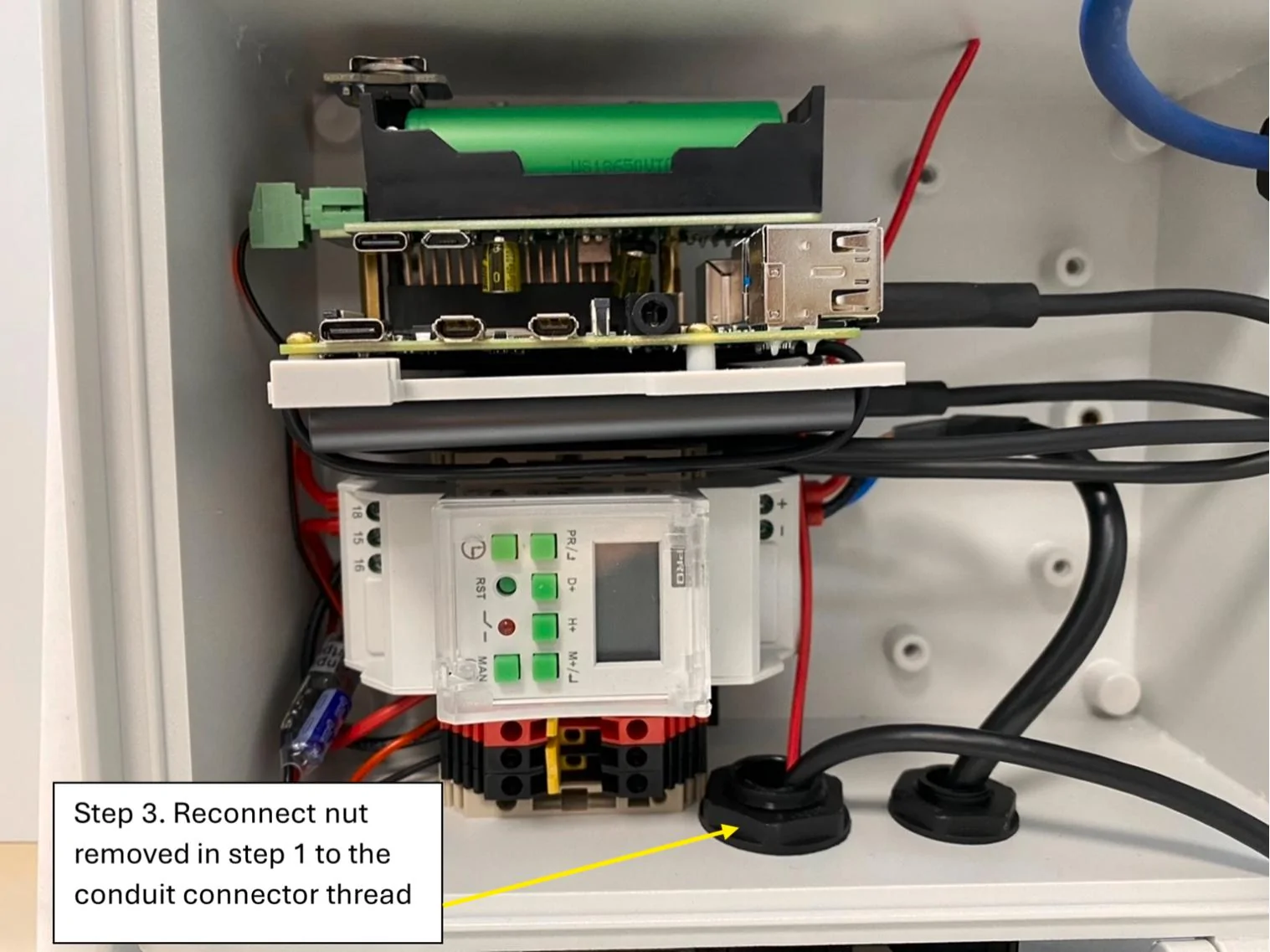
Wiring the camera and lighting ring into the electronics enclosure, step 3.
10. Connect the USB into the port shown in Fig. 12, step 4.
11. Connect the incoming 12 V supply to the positive and negative on the timer relay. Then link the positive to terminal 18 on the 12 V timer relay. Finally take a link wire from the negative on the timer relay to the black terminal block. Fig. 14 shows, in more detail how the timer relay should be wired up.

**Fig. 14:**
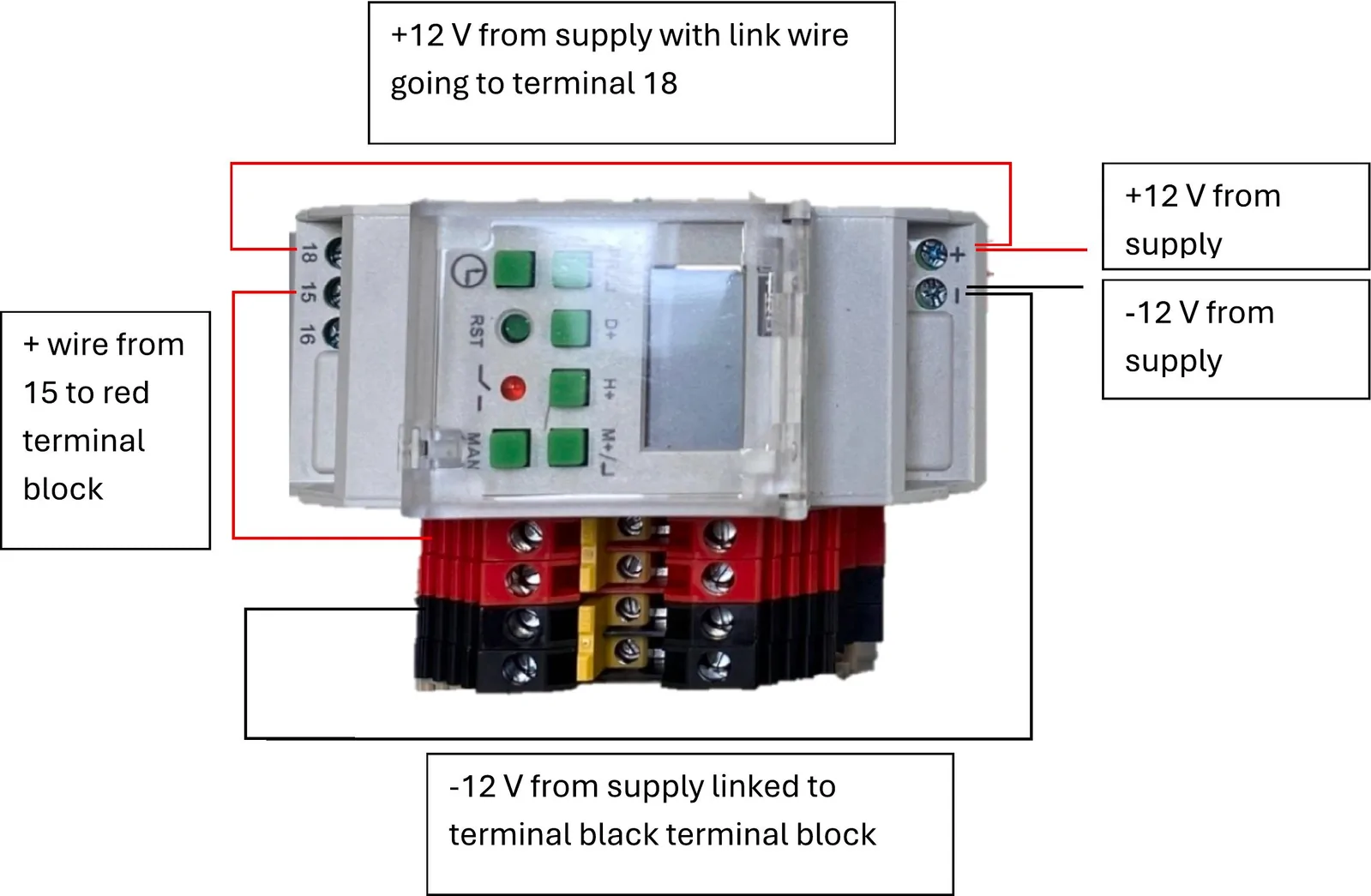
A schematic of the wiring of the timer relay, including connections from the 12 V power supply and connections to the terminal block.
12. Wire the other connections in the terminal block to the corresponding components as shown in Fig. 15.

**Fig. 15:**
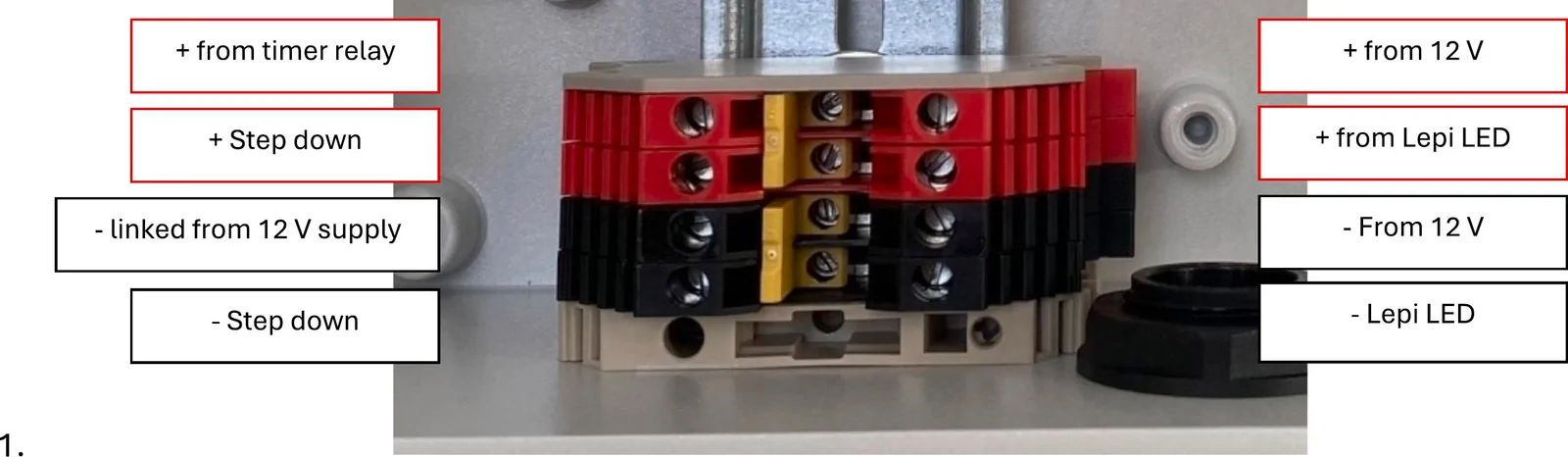
A schematic of the wiring into the terminal block.
13. Finally wire up the 12 V regulator as shown in Fig. 16.

**Fig. 16:**
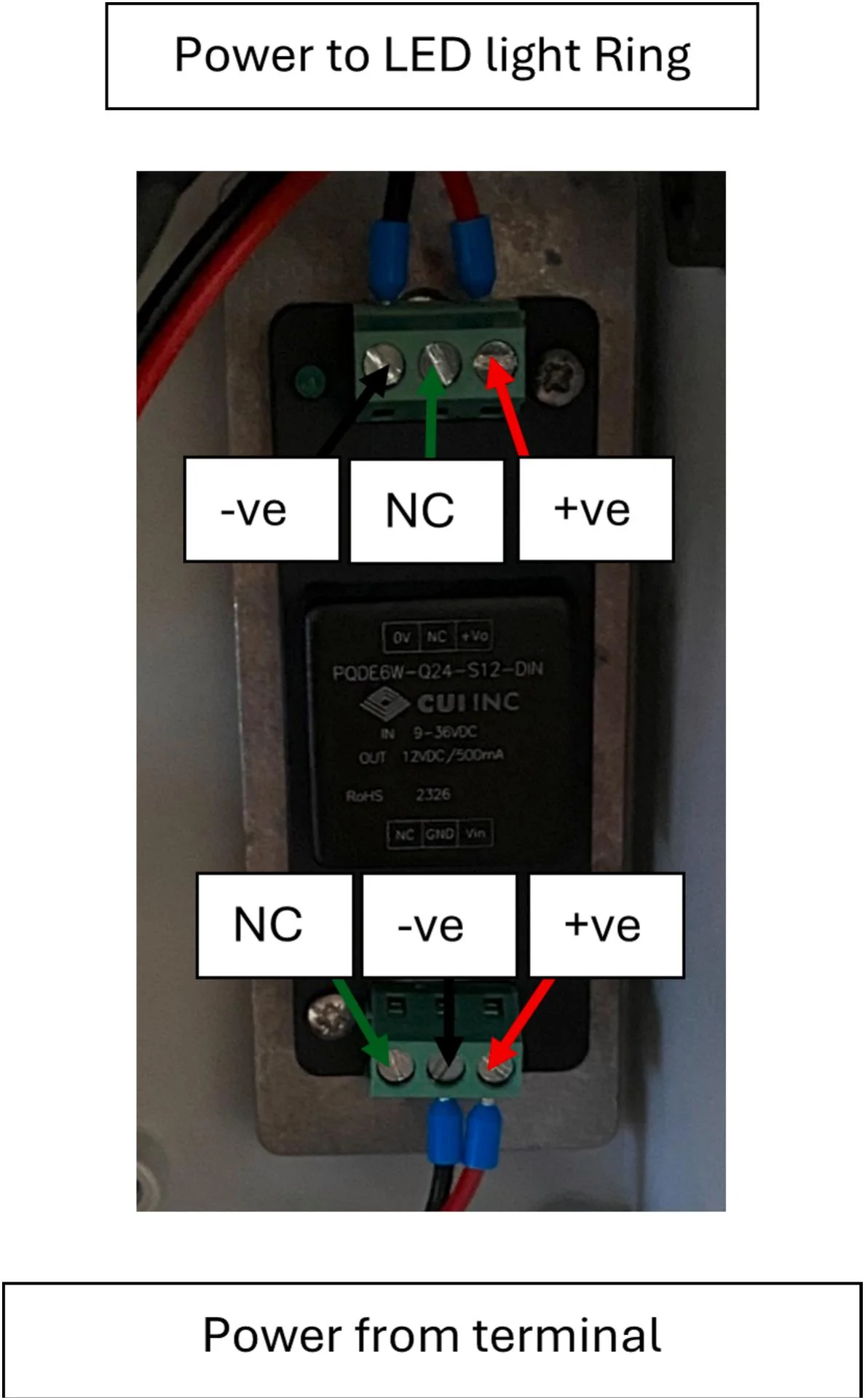
Wiring schematic for the 12 V regulator. See Fig. 17 for overview of electronics enclosure.

**Fig. 17:**
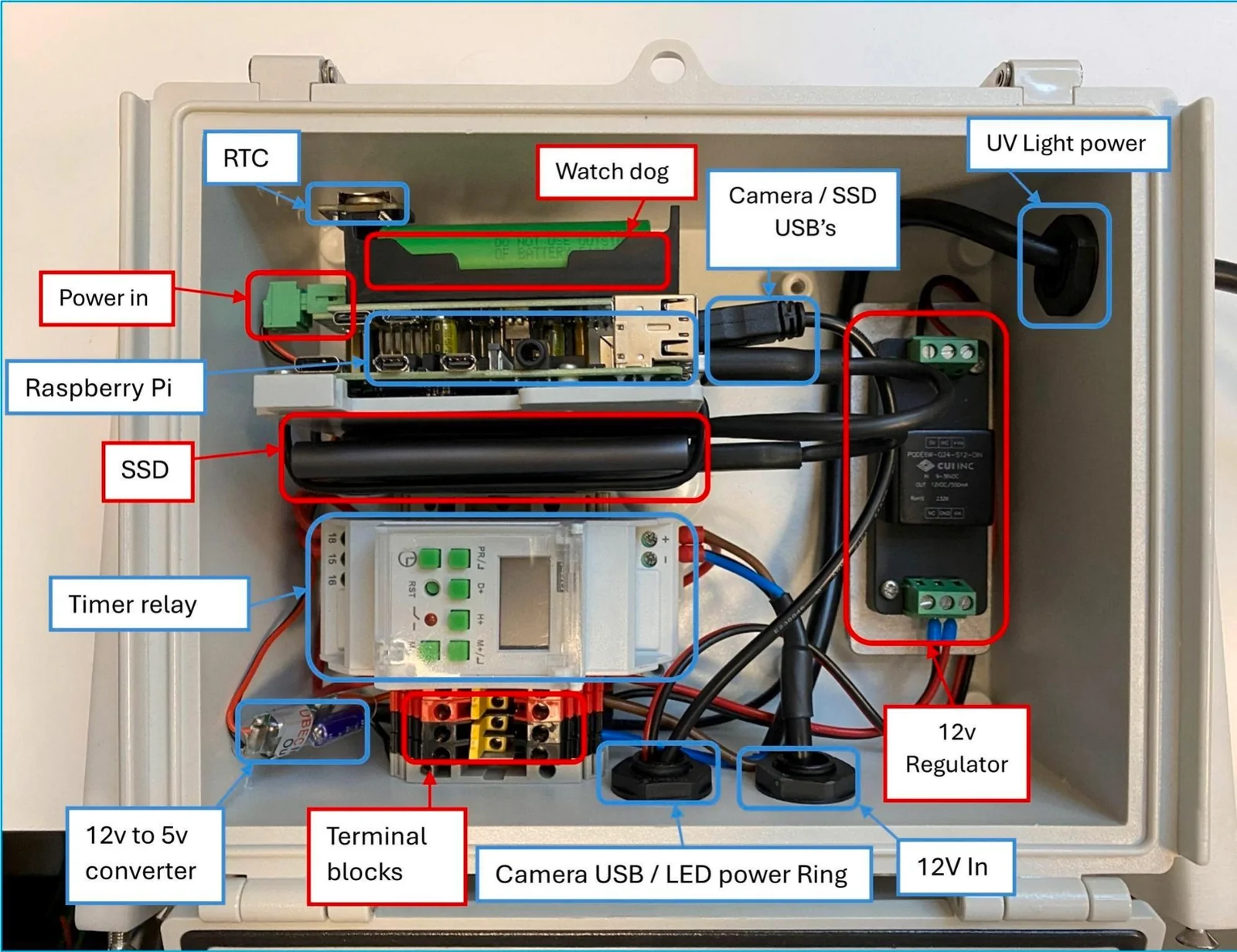
Fully assembled electronics enclosure.

The fully assembled electronics enclosure is shown in Fig. 17.

### 5.4 Assembling the backboard

**Fig. 18:**
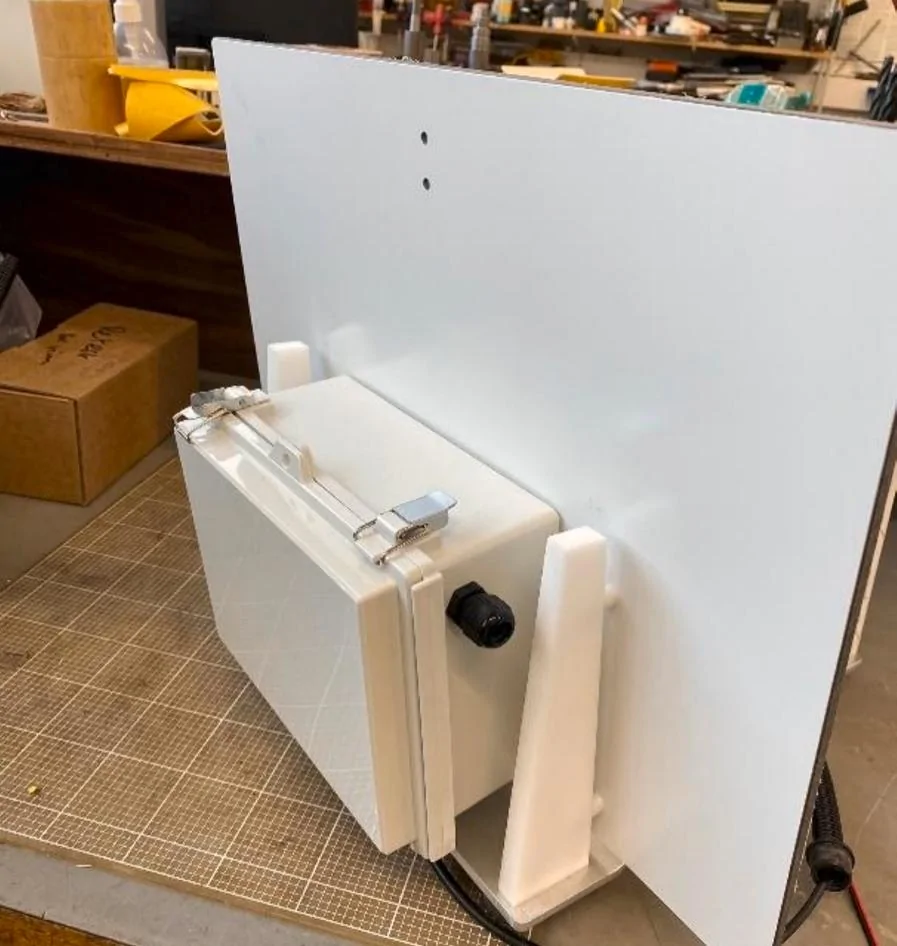
Back board attached to the electronics enclosure and uprights.

Mount the back board to the uprights using four 25 mm M5 hex countersunk bolts ensuring the spacers are between the upright and back board. Then mount the electronics enclosure to the back board using four poly fix 5 x 10 mm screws (Fig. 18).

### 5.5 Assembling the baseplate

**Fig. 19:**
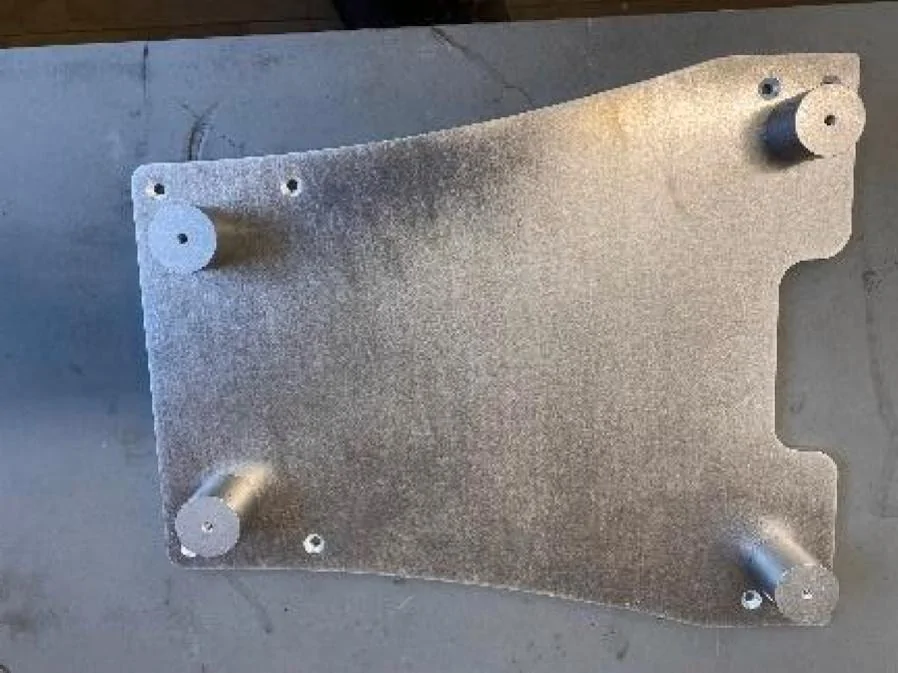
Standoffs attached to the underside of the baseplate.

1. Cut out and drill holes in the base plate as described in the drawing ‘DRAW3 Base plate V1.0.pdf’ [17].
2. Attach four standoffs (DRAW10 stand off V1.0.pdf) using four M5 x 16 mm countersunk socket screws (Fig. 19).

### 5.6 Assembling the system

1. Once all necessary parts are prepared, the backboard assembly and camera housing are mounted to the base plate as shown in Fig. 20.

**Fig. 20:**
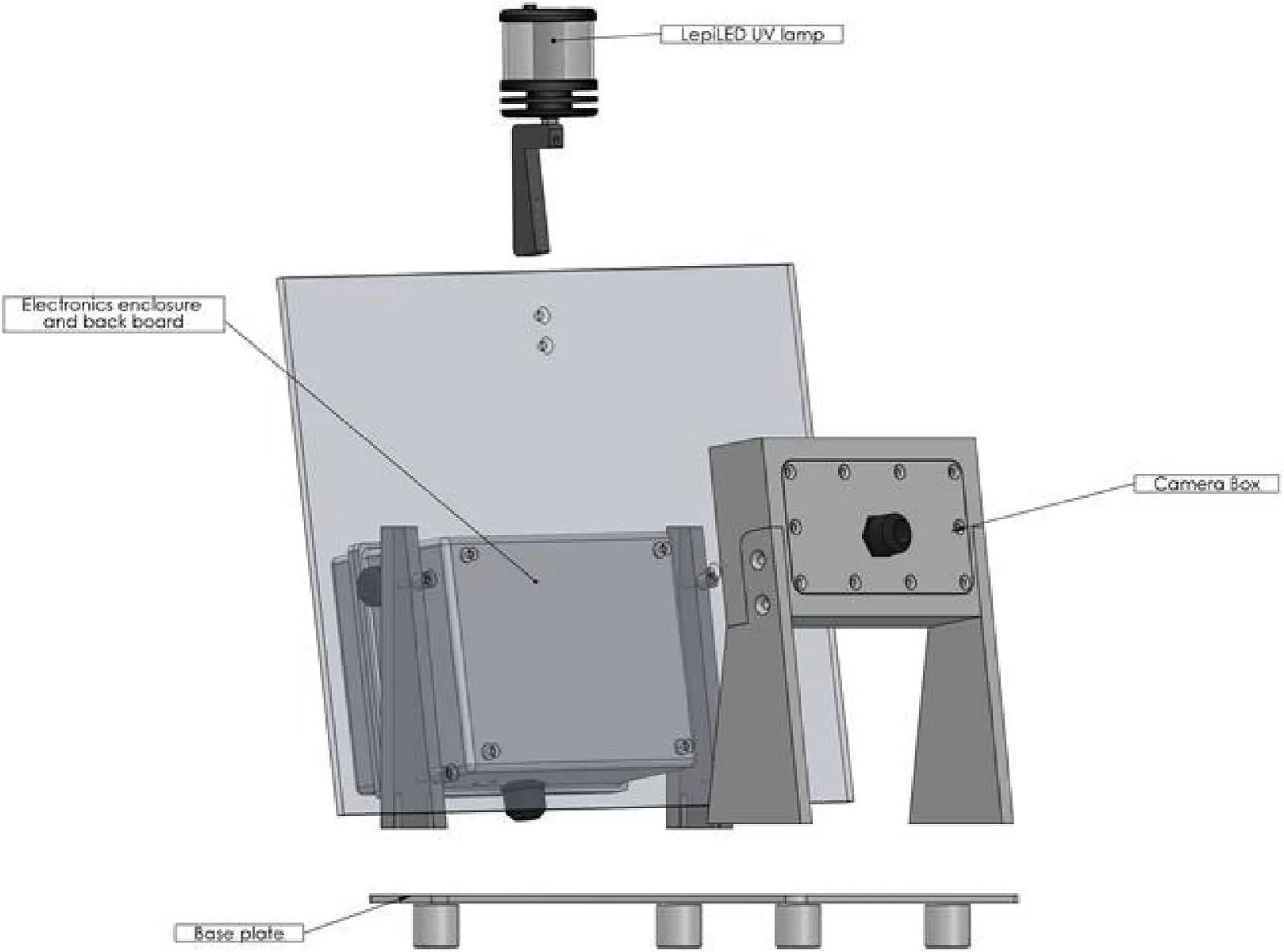
Assembling the UKCEH AMI-system.
2. The conduit carrying cables from the back of the camera enclosure to the electronics enclosure is routed under the baseplate, from front to back and between the standoffs (Fig. 21).

**Fig. 21:**
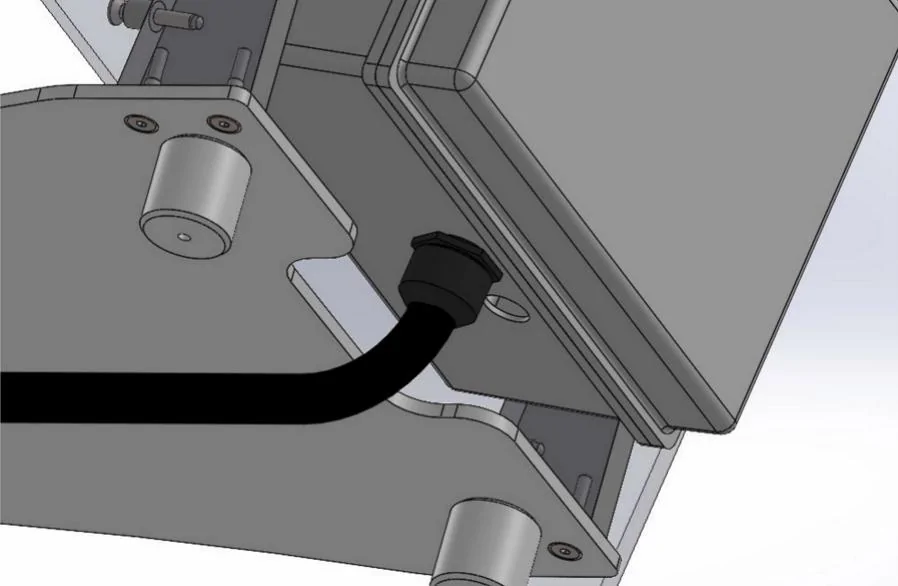
Cable routing from the camera housing to the electronics enclosure.
3. The Lepi LED is then mounted to the back board using the UV light bracket as shown in Fig. 22. Finally, mount the UKCEH AMI-system (Fig. 23) to the battery box lid.

**Fig. 22:**
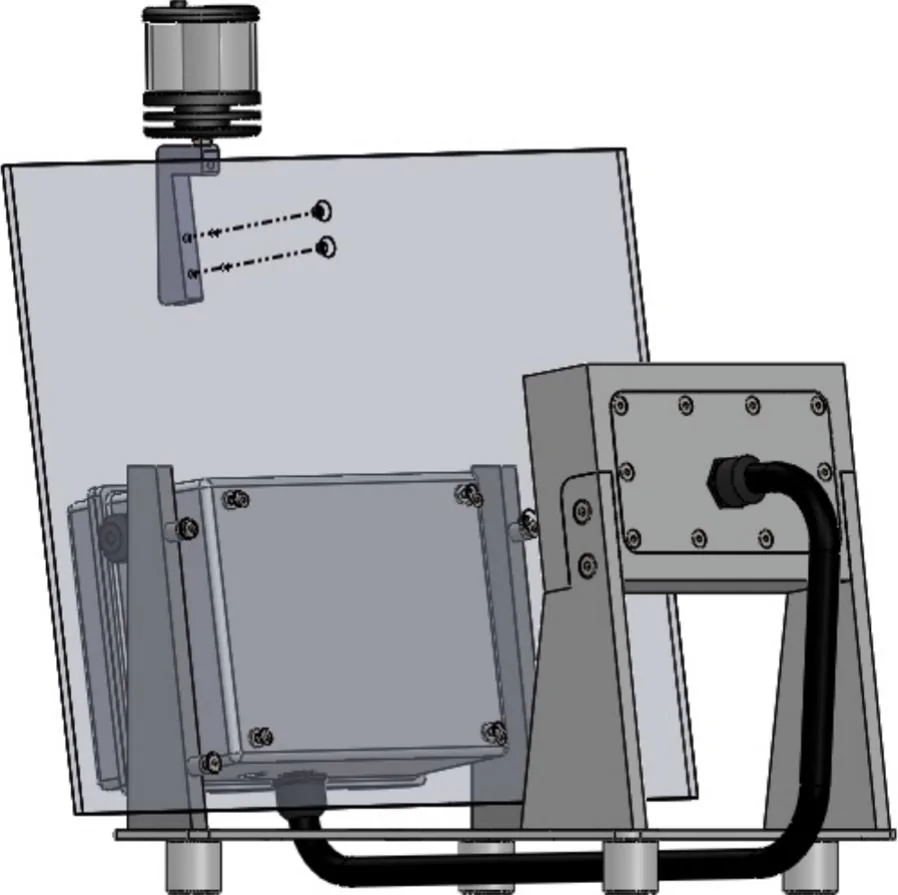
Attaching the Lepi LED.

**Fig. 23:**
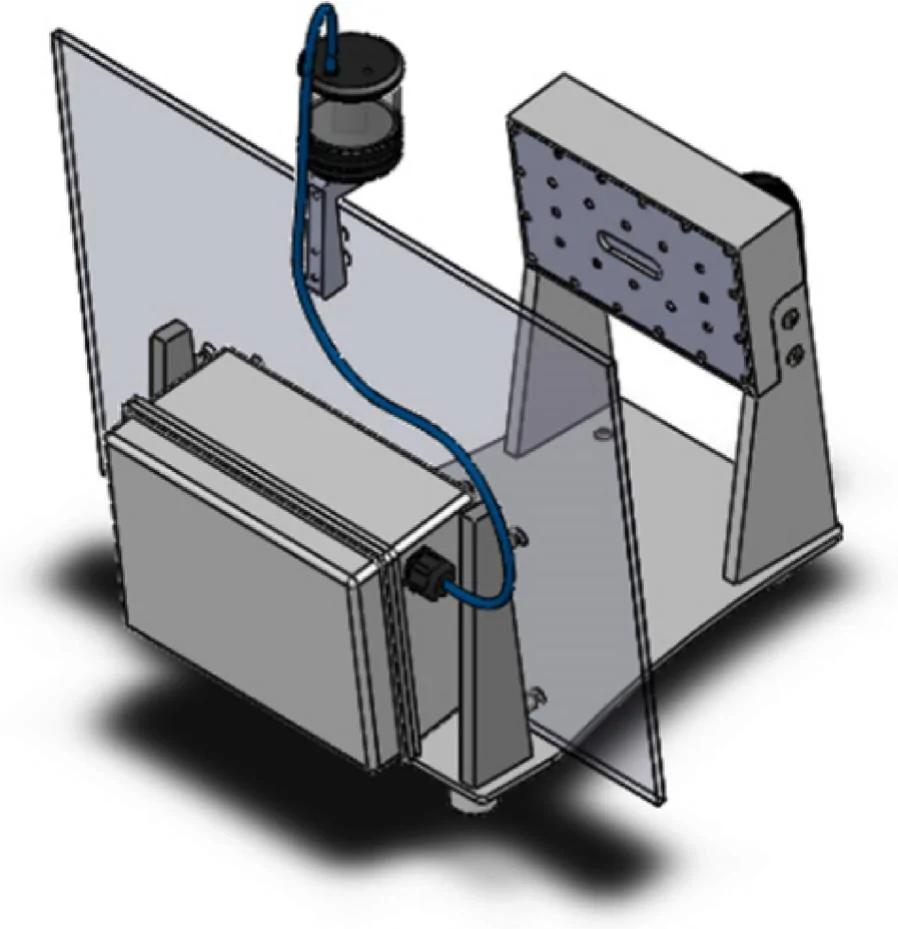
Final assembly view.

## 6 Operation instructions

Please be aware once the instrument is connected to a power supply, if the white lights come on this will mean the UV light is also on. This contains ultraviolet rays and can be harmful to look at. Please take extra caution, covering the lamp or wearing UV protective glasses. The light can be de-activated by disconnecting it, but in normal conditions the UV light will always be on when the system is on.

Prior to operation, the UKCEH AMI-system needs configurations and settings as below:

Flash the Raspberry Pi OS image [17] onto the 64 GB SD card. This image has the required software for operating the system installed. To connect to the systems, plug a computer into the RaspberryPi using an ethernet cable and use SSH. The address for the Raspberry Pi is pi@raspberrypi.local and the default password “Nature17”. Change the password after first connecting to ensure security.

### 6.1 Setting time on the real timeclock

The most effective way of setting time on the RTC is to connect the Pi to the internet, doing so will sync the Pi’s time to an NTP (Network Time Protocol) server automatically. Performing this step requires only the Raspberry Pi and all the attached hats, and can be done prior to installing these into the electronics enclosure. If an internet connection is not available the following method can be used.

1. Open the terminal on the Raspberry Pi and check the time with the command ‘timedatectl’
2. If necessary, update the time zone, for example ‘sudo timedatectl set-timezone Europe/London’.
  a. You can see a list of timezones using ‘sudo timedatectl list-timezones’.
3. Check the timezone has been updated by running ‘timedatectl’.
4. Update the date and time to the current time e.g. ‘sudo date −s “2025–12-30 14:52”.
5. Verify the date and time by running ‘date’.
6. Then write the time into the RTC battery with the following command ‘sudo hwclock −w’.
7. Confirm the RTC battery time is correct with ‘sudo hwclock −r’.

### 6.2 Testing the UKCEH AMI-system

1. Set up and configure the timer relay following the manufacturer’s instructions. The 12 V timer relay is procured directly from RS Components, part number 8966891.
2. Using the timer relay, turn the system ON.
3. Cover the UV light/LepiLED to protect yourself from the UV light. This is a precautionary measure but is recommended if working close to the light for extended periods of time.
4. Once the AMI is on it will begin to take one image every ten seconds.
5. After a minute, use the timer relay to turn off the system.
6. Once the AMI is off, wait until the watchdog finishes switching off the Raspberry Pi, this will be apparent when all the LEDs on the Raspberry Pi turn off.
7. Take the SSD out and connect it to your computer to see if the images have been taken properly and the filenames have the correct time.

### 6.3 Solar panel and battery recommendations

For field deployments requiring long-term autonomous operation, we recommend the following solar panels and batteries, to be used with a Sunsaver 20 L solar charge controller:

• 2x 100 W solar panels in parallel (12 V panels)
  o Max open circuit voltage (Voc): 25 V.
  o Max short circuit current (Isc): 25 A.
• 2 x AGM lead acid deep cycle / marine batteries in parallel (12 V)
  o 120 AH 12 V Absorbed Glass Mat (AGM) or Marine Lead Acid Battery.

This would support an operational program turning the instrument on for 10 h a night every 3rd night (tested at latitude 52 degrees North), however additional batteries and panels will be required in areas that are shaded, or experience limited sunlight, such as in high latitudes.

To help calculate a solar and battery system for different conditions we have included a table (Table 5) which shows the power consumption of each part.

**Table 5:** Power consumption breakdown.

**Component**	**Power consumption**
Raspberry pi (including connected camera and SSD)	c.a 1.7w
Lepi LED	c.a. 8 W
Light ring PCB v2	c.a 4.4 W
Timer relay	Negligible

## 7 Validation and characterization

**Fig. 24:**
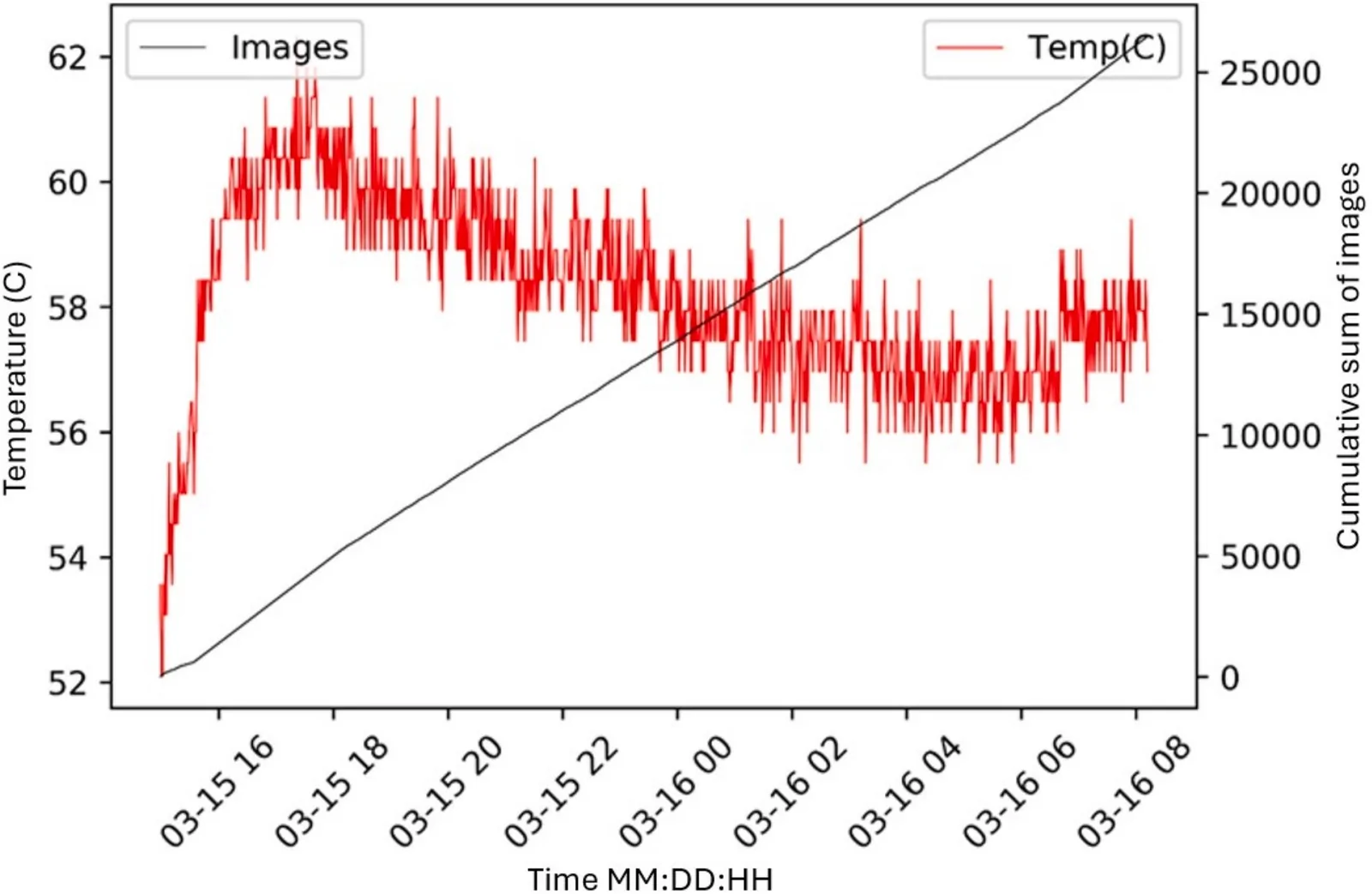
Results of running the UKCEH AMI-system through a heat test shows that the system continued to capture images at a resolution of 4096 x 2160 continuously in temperatures of 59°C +/-3 over a period of 16 h.

The UKCEH AMI system is engineered to operate reliably in both tropical and temperate climates, meaning it must withstand high humidity, heavy rainfall, and elevated temperatures. To assess its heat resistance, the camera housing and electronics enclosure were tested in a climate–controlled chamber. The environment was heated to 59 °C (±3 °C) while the system remained fully operational. During the test, the system was positioned in front of an object that triggered image capture once per second, ensuring continuous activation and photo storage throughout the heat–stress trial (Fig. 24).

To ensure the camera housing was watertight we replicated an IPx7 waterproof test. This involved submerging the housing in water for 30 min at a depth of one meter, the housing was then removed and visually inspected for any signs of water ingress. During this test no water entered the camera housing.

Whilst all the components used in the UKCEH AMI-system are electrical safety compliant, we additionally tested the system as a whole to ensure the electrical safety of the device. Continuity testing of the circuitry and EMC testing through an electrical compliance test house identified no electronics issues (Table 6).

**Table 6:** Summary of electrical testing.

**Test**	**Basic Standard**	**Test Level / Frequency Range**	**Mod.**	**Result**
DC Port Conducted Emissions	EN 61000–6-4:2019	Class A / 150 kHz to 30 MHz	0	Pass
Radiated Emissions up to 1 GHz	EN 55016–2-3:2010 + A1:2010 + A2:2014	Class A / 30 MHz to 1 GHz	0	Pass
Radiated Emissions > 1 GHz	EN 55016–2-3:2010 + A1:2010 + A2:2014	Class A / 1 GHz to 6 GHz	0	Pass
Electrostatic Discharge	EN 61000–4-2:2009	±2 kV, ±4 kV Contact ± 2 kV, ±4 kV, ±8 kV Air	0	Pass
Radiated Field Immunity	EN 61000–4-3:2006 + A1:2008 + A2:2010	3 V/m, 80% 1 kHz AM 80 MHz to 2.7 GHz	0	Pass
Radiated Field Immunity	EN 61000–4-3:2006 + A1:2008 + A2:2010	3 V/m, 80% 1 kHz AM 1.8 GHz, 2.6 GHz, 3.5 GHz, 5.0 GHz Spot Frequencies	0	Pass
EFT / Bursts	EN 61000–4-4:2012	±0.5 kV DC Power	0	Pass
Surge Immunity	EN 61000–4-5:2014	±0.5 kV DC	0	Pass
Conducted RF Immunity	EN 61000–4-6:2014	3 V, 80% 1 kHz AM 150 kHz to 80 MHz	0	Pass
Magnetic Field Immunity	EN 61000–4-8:2010	1 A/m, 50 Hz	0	Pass

**Fig. 25:**
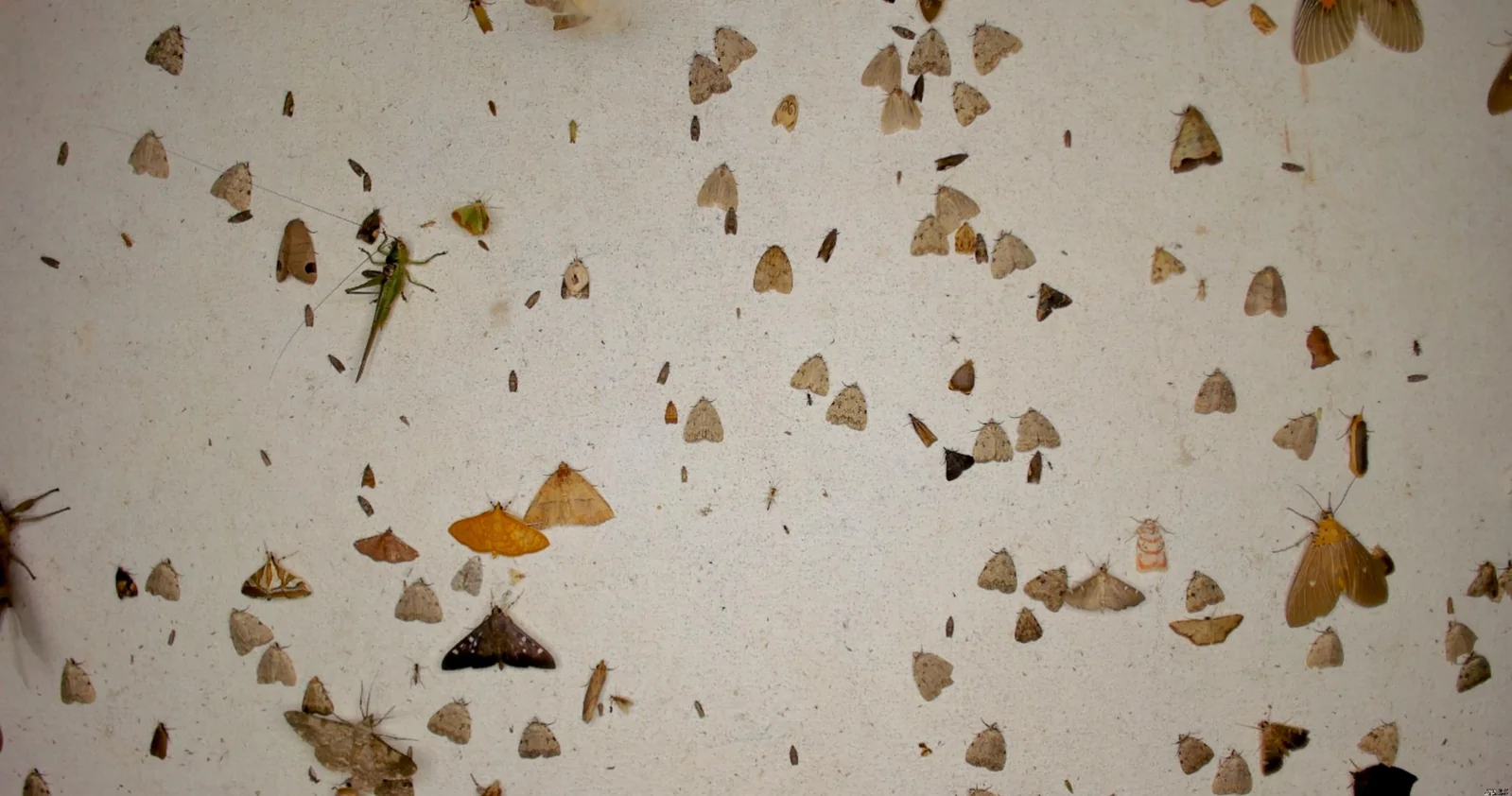
Example image from an UKCEH AMI-system deployed in Thailand. Images have a resolution of 4096 x 2160 pixels.

As previously mentioned, nearly 200 UKCEH AMI-systems are deployed globally. These systems have collected many thousands of pictures an example of which is shown in Fig. 25. In the United Kingdom we run the systems for one night in every three, with three batteries and two 100 W solar panels. This setup is enough to power the system from early May, through to late September. With a 500 GB SSD we find this is able to hold approximately 340,000 pictures. Our standard schedule is to take one image every 60 s between sunset and one hour before sunrise, meaning that one SSD can last an entire field campaign (May-September). Given the number of units in use we have also been able to identify common issues that users should be aware of. After prolonged periods in storage we recommend checking and replacing the Lithium 18650 battery and the RTC CR 1220 battery. In some cases these have lost charge and caused problems with subsequent deployments. When closing the electronics enclosure, for example after swapping the SSD, it is important to check that cables are not trapped in the seal as this can allow water to enter the electronics enclosure. We recommend testing each system at the office/lab before deployment so that any issues can be identified before going to the field.

**Fig. 26:**
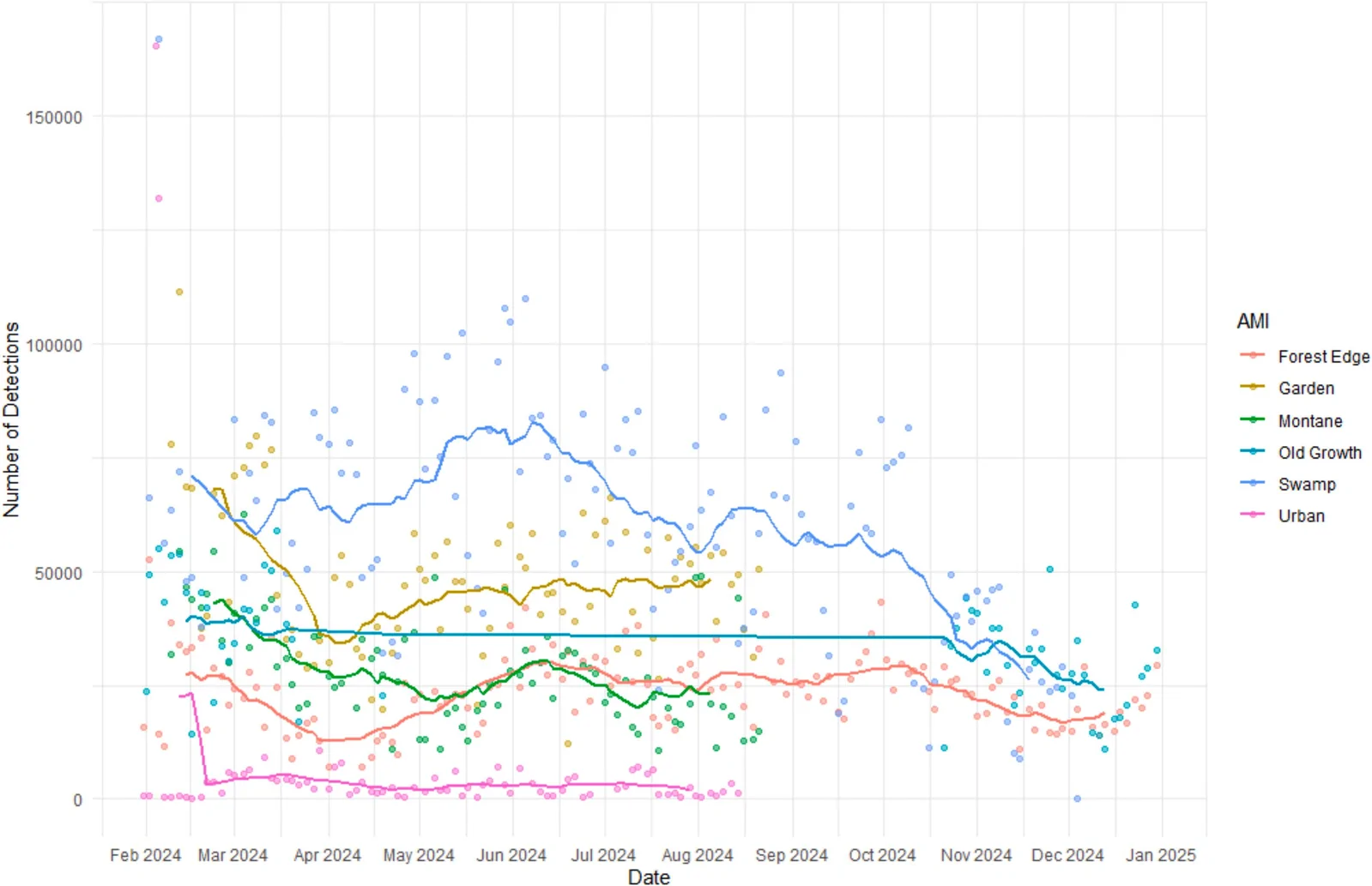
Plot of the number of moths detected each night across 6 UKCEH AMI-systems deployed in Costa Rica. Points give raw numbers per night and lines represent a 14-day moving average. In this analysis the same individual moth in multiple images will be counted as multiple detections. An AI classifier has been used to remove non-moth species from the data. The graphs demonstrate the variability we observe in the number of moths that come to the trap across nights, seasons and locations, offering the possibility to explore drivers of these trends, such as habitat, weather and climate. In this example it is notable that the urban site has very low number, as would be expected.

As evidence of the AMI functionality, we here show data from a network of systems that have been located at a number of sites in Costa Rica. The images from these systems have been analysed with computer vision tools to count all instances of insects on the backboard. The analysis of these data (Fig. 26) shows the variability in the number of detections made between days, sites, and across the seasons. For this analysis we used a generic detector to find insects within images, and a moth/non-moth classifier to present data only on moths. These models were created by Jain *et al* 2024 [22] whose training data [23] and code [24] are openly available. An additional example of the use of the UKCEH AMI-systems can be found in Bett *et al.* 2025 [25], where the authors deploy an AMI system in Kenya, reporting an average of 10 insects per night over two months.

Artificial intelligence models have been developed for detecting and identifying insects in images from UKCEH AMI systems and analogous devices. These include accurate detection algorithms (F1 score 94%) that locate insects within the images, enabling them to be isolated for downstream classification [26]. Individual insects on the landing board can also be tracked, ensuring that the same individual present across multiple images is only counted once [7]. However, this cannot account for individuals that leave the board and return later. Classifiers are being developed that can identify not only species, but also higher taxonomic levels, allowing for identification to ‘roll-up’ to higher taxonomic levels (e.g. Genus), when the species is uncertain [27]. Investigation into time-lapse vs motion triggered recordings also suggests that time lapse intervals of 2 min – 10 min show comparable patterns to motion triggered data [28]. Already papers are emerging that are using the high temporal resolution data from automated insect camera traps to generate new ecological insights [29], [30].

## 8 Human and Animal Rights

This study involved non-lethal light trapping of invertebrates only. No regulated animal experiments were conducted, so ARRIVE guidelines and animal legislation do not apply. Landowner permission was obtained for all deployments.

## CRediT authorship contribution statement

**William Lord:** Writing – review & editing, Writing – original draft, Methodology, Investigation, Conceptualization. **Simon Teagle:** Writing – review & editing, Writing – original draft, Methodology, Investigation, Conceptualization. **Joshua Alton:** Methodology, Investigation. **Gabriel Bannister:** Methodology, Investigation. **Jonas Beuchert:** Writing – review & editing, Validation, Methodology. **Kim Bjerge:** Writing – review & editing, Software, Methodology, Investigation, Conceptualization. **Dylan Carbone:** Validation, Investigation, Formal analysis. **Alba Gomez Segura:** Validation, Software, Methodology, Investigation. **Tim Howson:** Methodology, Investigation. **Toke Thomas Høye:** Conceptualization, Funding acquisition, Methodology, Writing – review & editing. **Jenna Lawson:** Validation, Investigation, Formal analysis. **Abhi Ravivarma:** Writing – review & editing, Writing – original draft, Validation, Methodology, Investigation. **David B. Roy:** Funding acquisition, Validation, Writing – review & editing. **Dan Rylett:** Writing – review & editing, Methodology, Conceptualization. **Grace Skinner:** Validation, Investigation, Formal analysis. **Alan Warwick:** Methodology. **Tom August:** Writing – review & editing, Writing – original draft, Validation, Investigation, Funding acquisition, Conceptualization.

## Declaration of competing interest

The authors declare that they have no known competing financial interests or personal relationships that could have appeared to influence the work reported in this paper.
