# Supplementary material for: Automated monitoring of insects system: building a non-lethal and scalable solution for monitoring of nocturnal insects
Source: HardwareX. 2026 Aug 6;27:e00822. doi: 10.1016/j.ohx.2026.e00822 (PMC13499100; doi:10.1016/j.ohx.2026.e00822)

UKCEH AMI-System Build Manual

The image below shows an assembled AMI system with the main components labelled. This can be used to refer to throughout the manual to see where parts are located in the system.

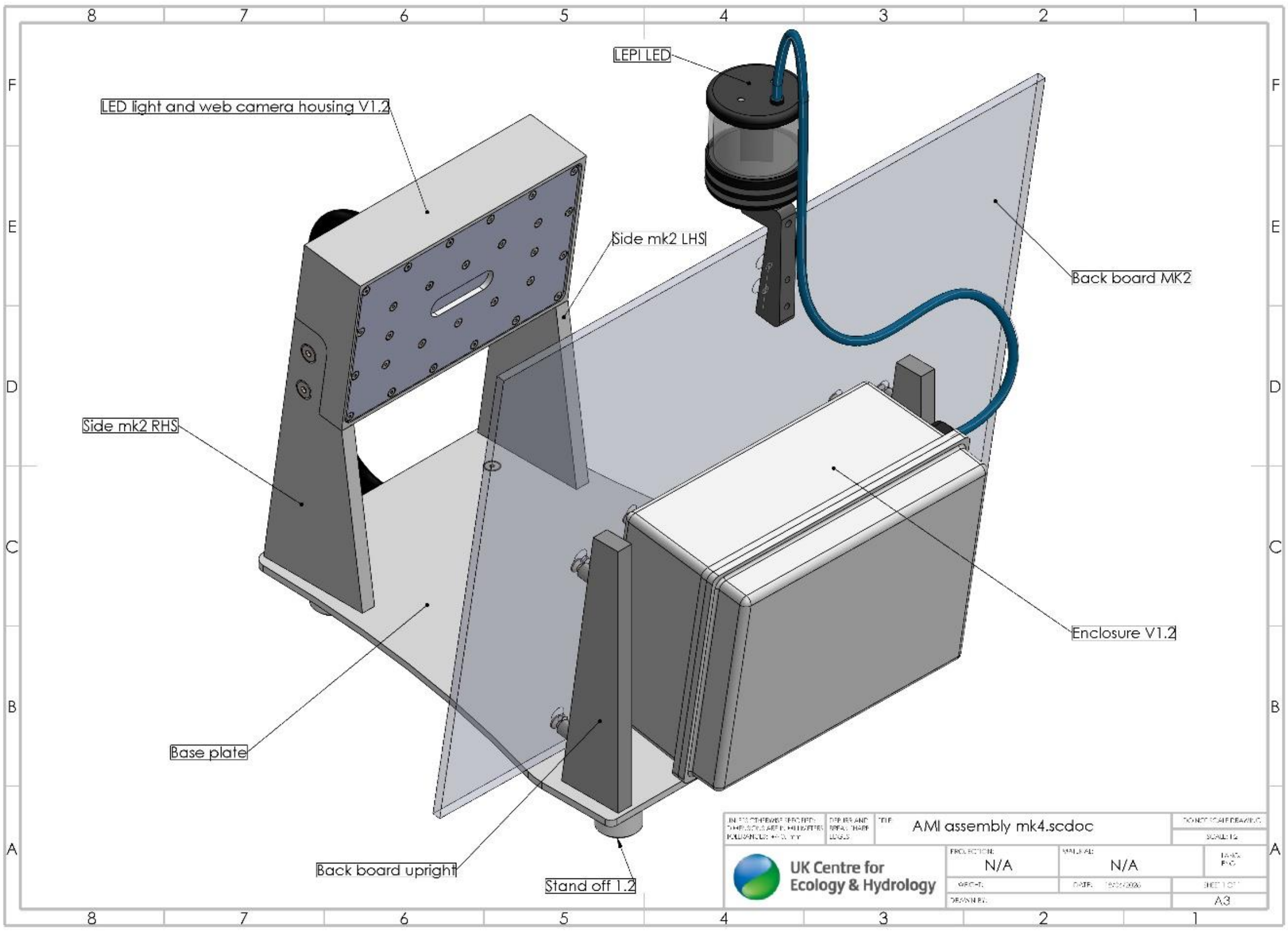

Base plate

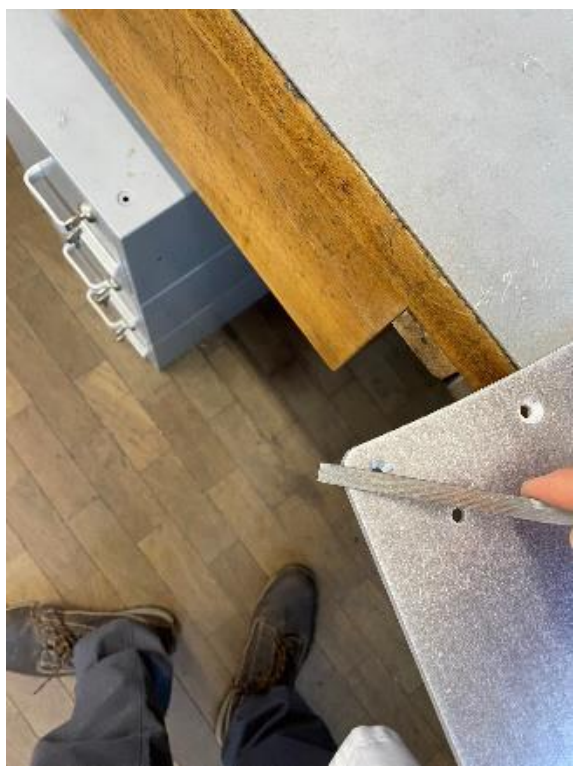 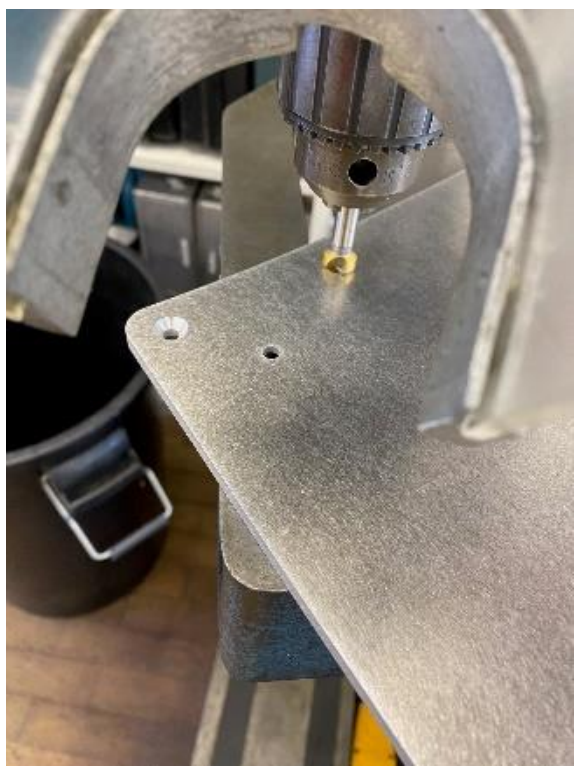 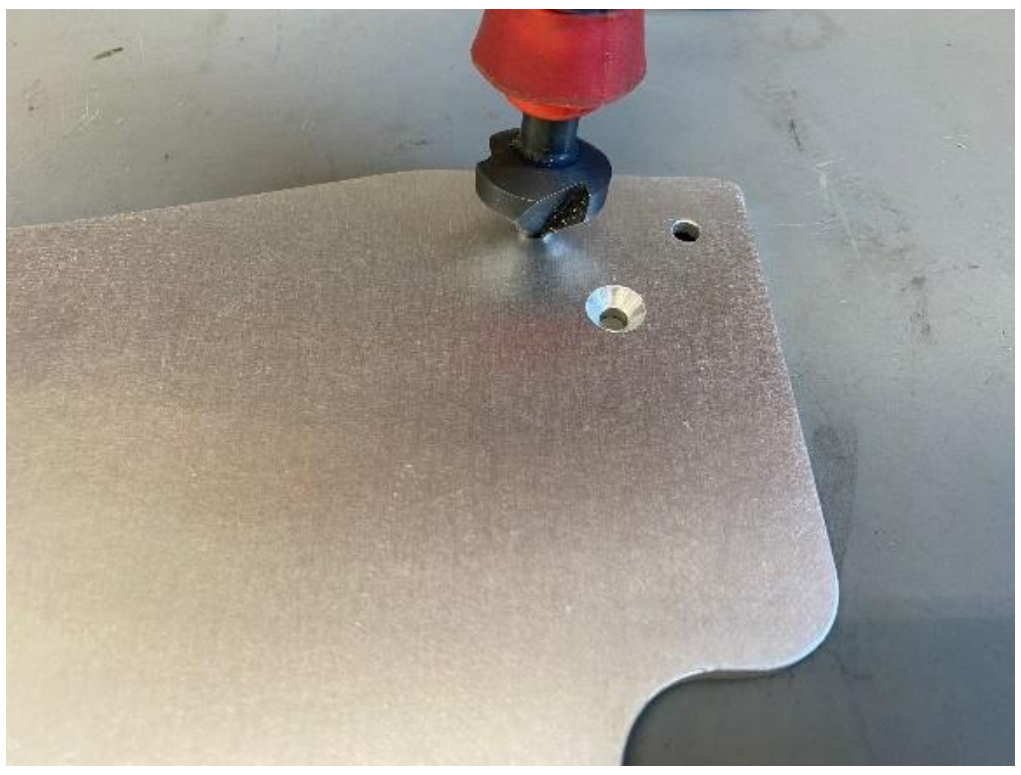

Cut and drill the base plate using the plans in ‘DRAW3 Base plate V1.0’. Deburr all sharp edges and

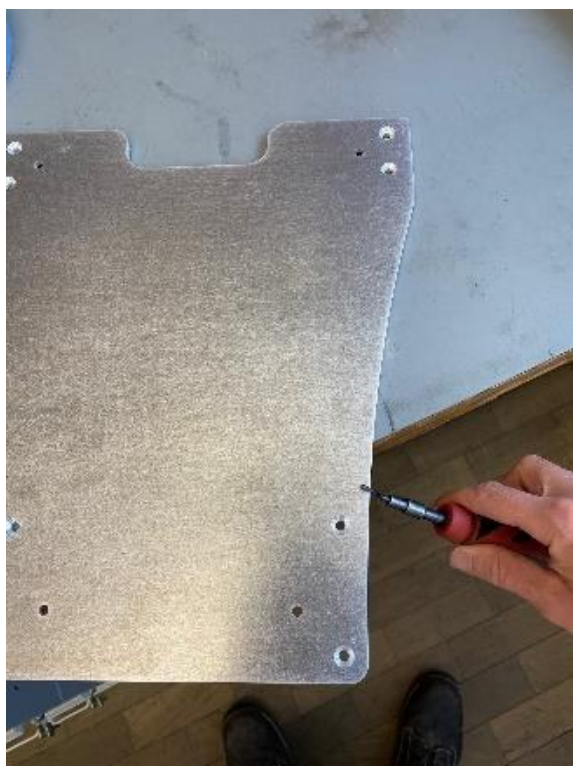

counter sink holes as per drawing.

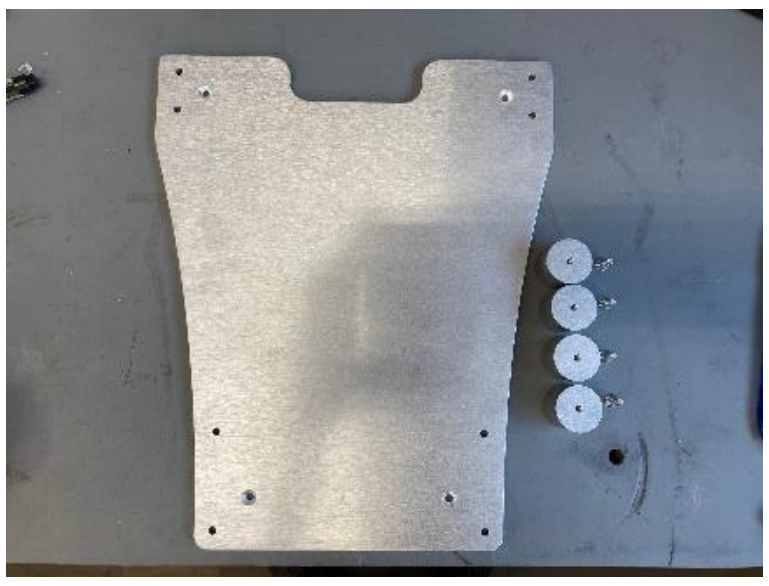 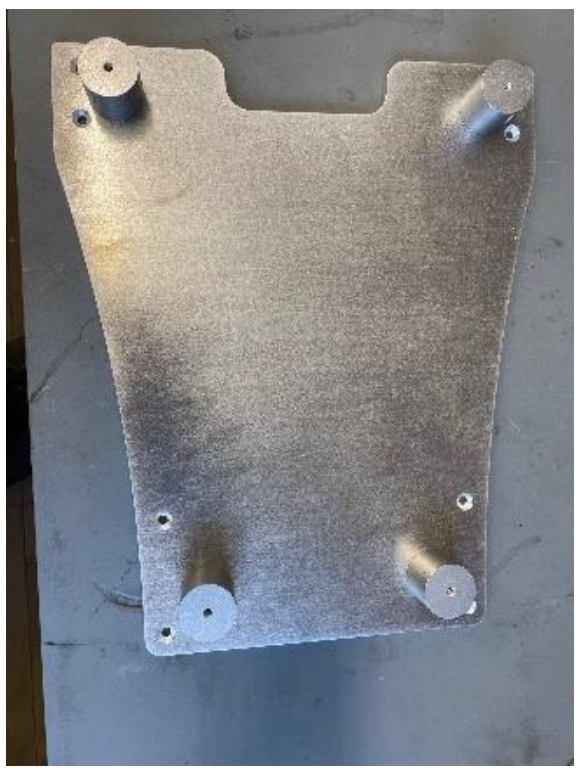 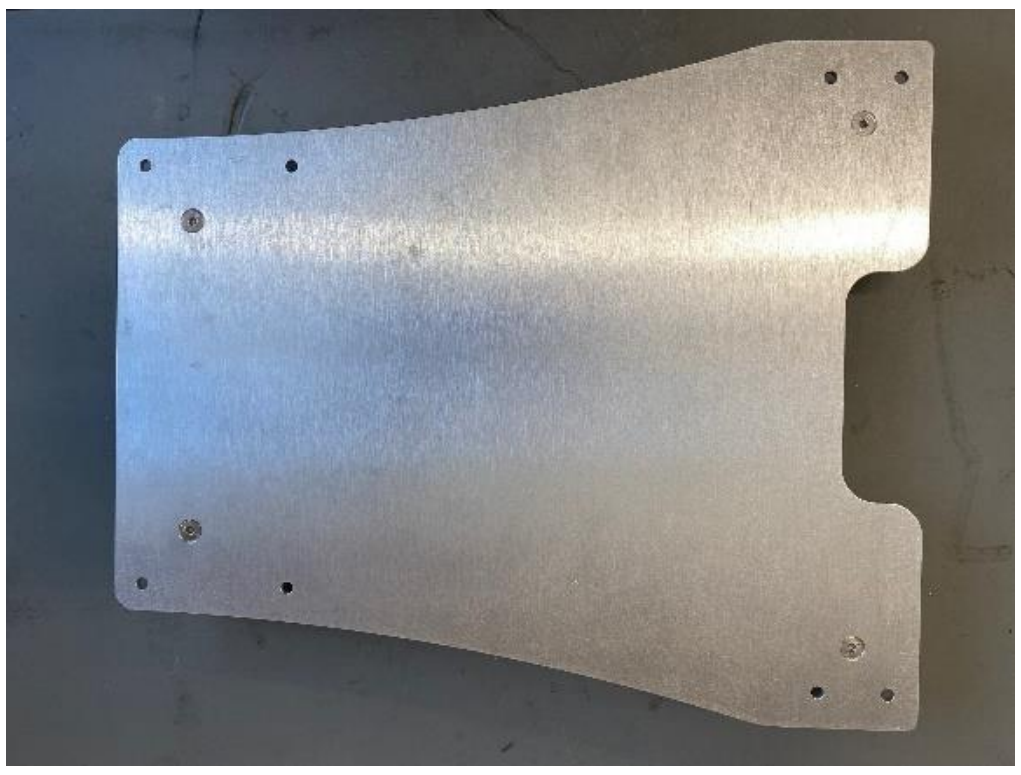

Assemble the base plate using 4 x standoffs (see drawing ‘DRAW10 stand off V1.0’) and 4 - m5x 16mm countersunk socket screws.

Back board

Cut and drill the backboard using the plan in 'DRAW1 back board MK2 V1.0'.

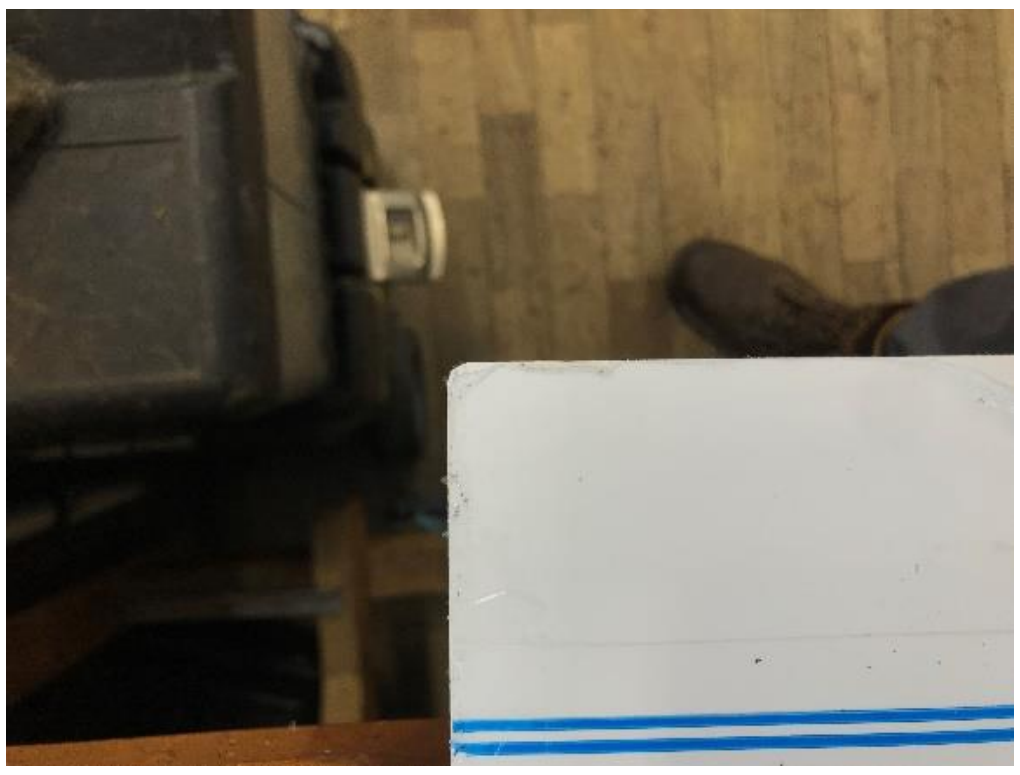

Put radius on corners with file.

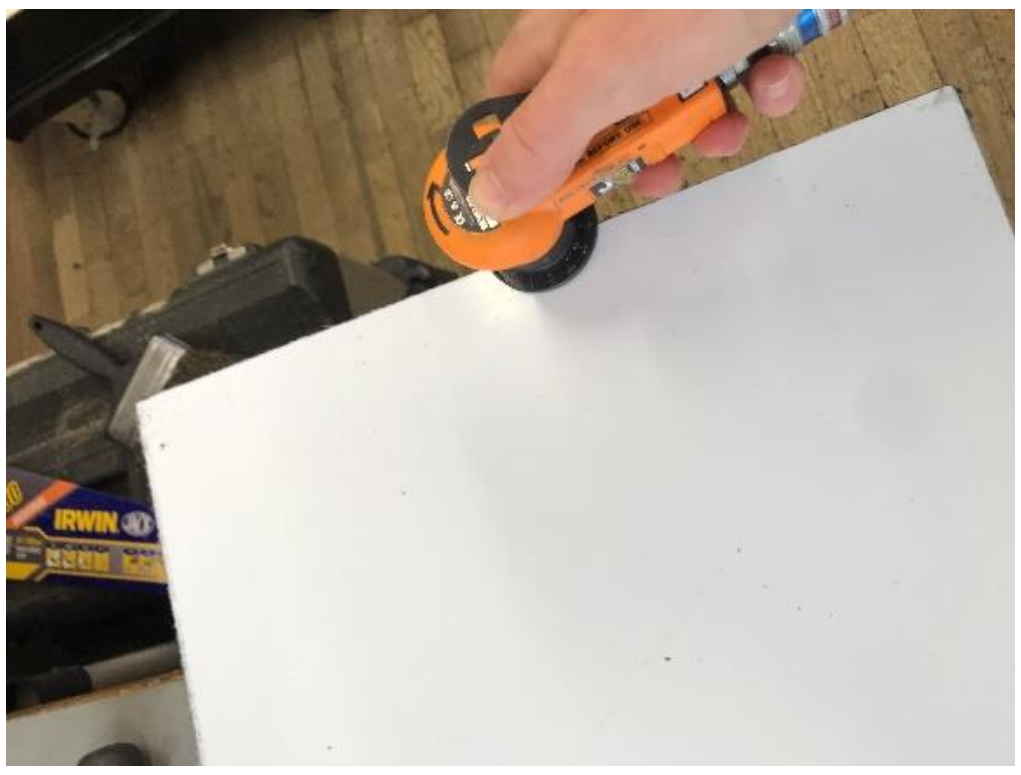

Deburr edges using deburring tool.

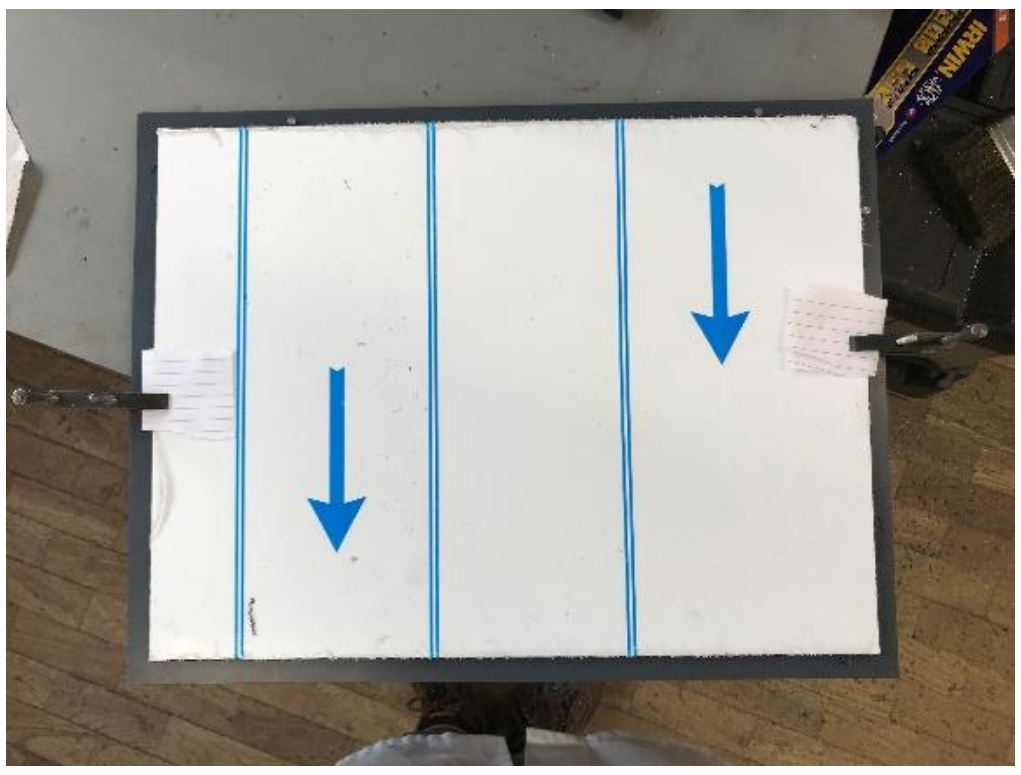

Clamp board to jig using tool makers clamps.

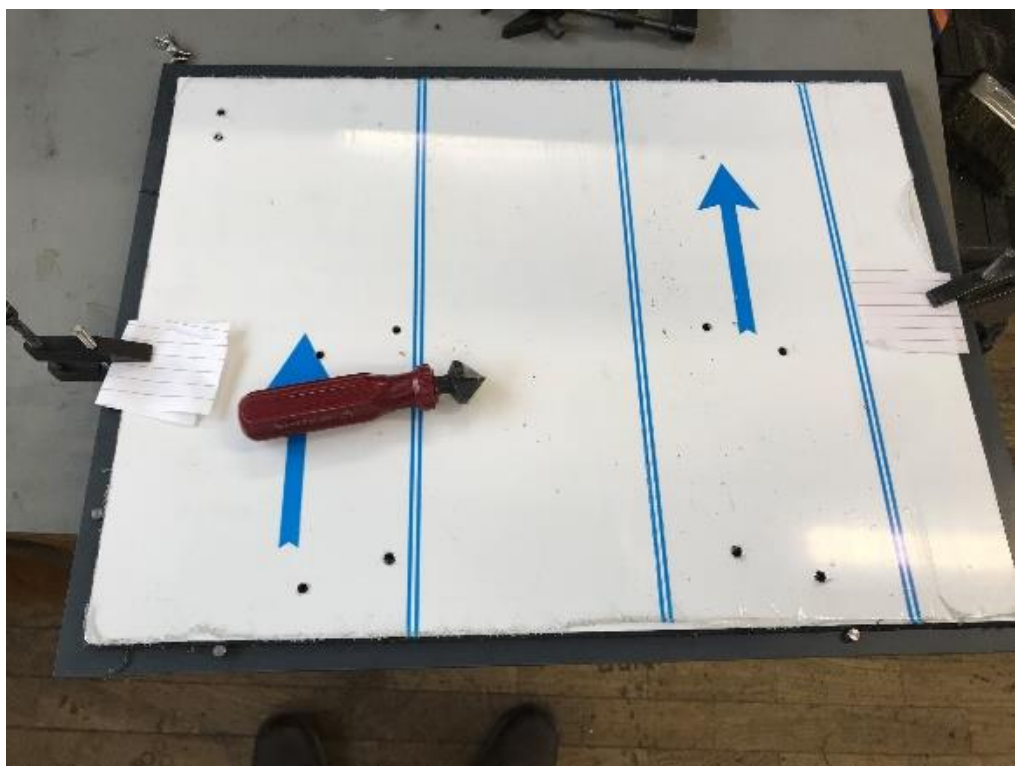

drill holes 5.3mm using pillar drill and countersink to remove burrs.

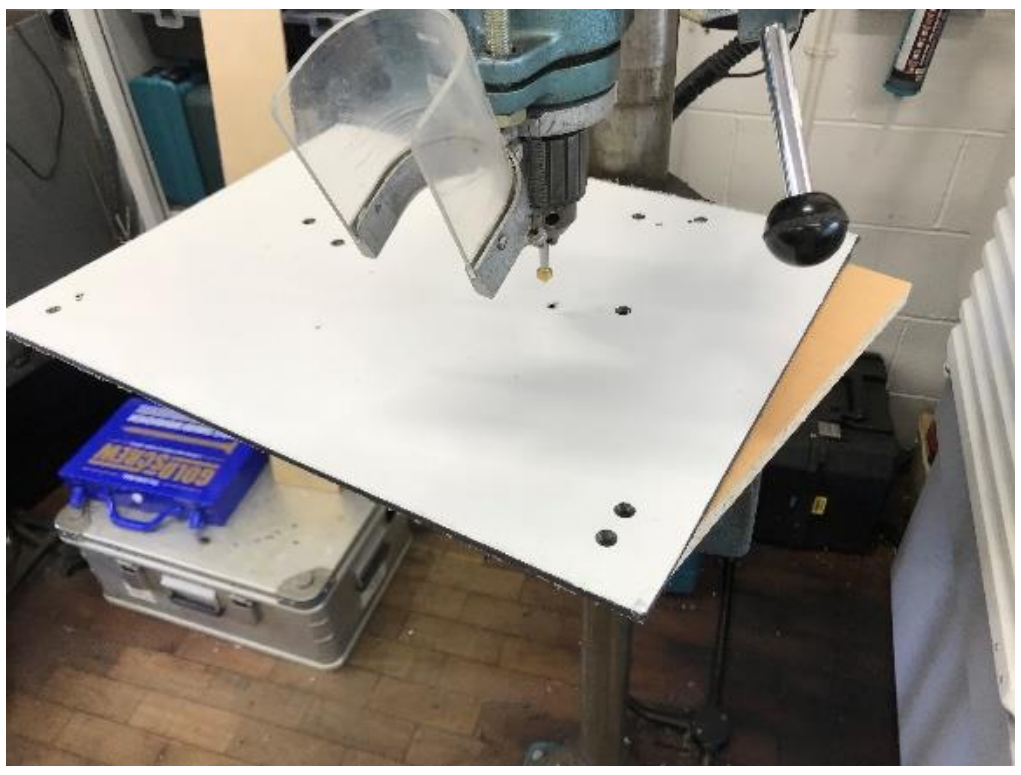

Countersink holes as per drawing.

Back board assembly

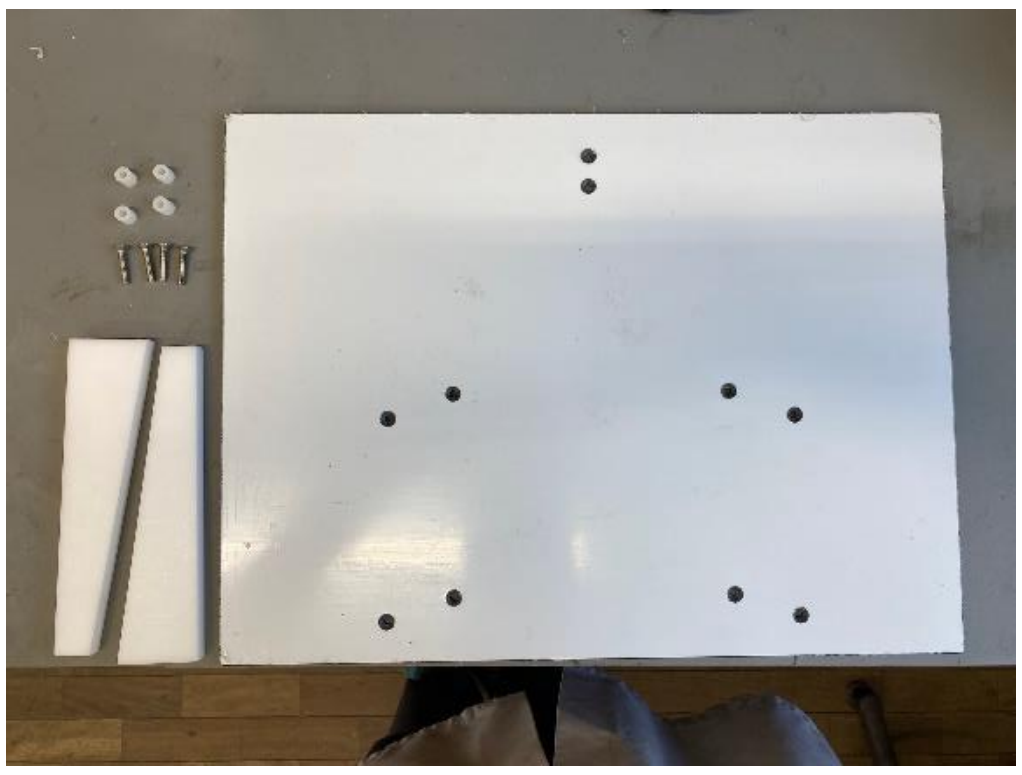

Parts : 1 x back board
2 x uprights
4 x m5 x 25 socket counter sunk screw
4 x nylon spacers.

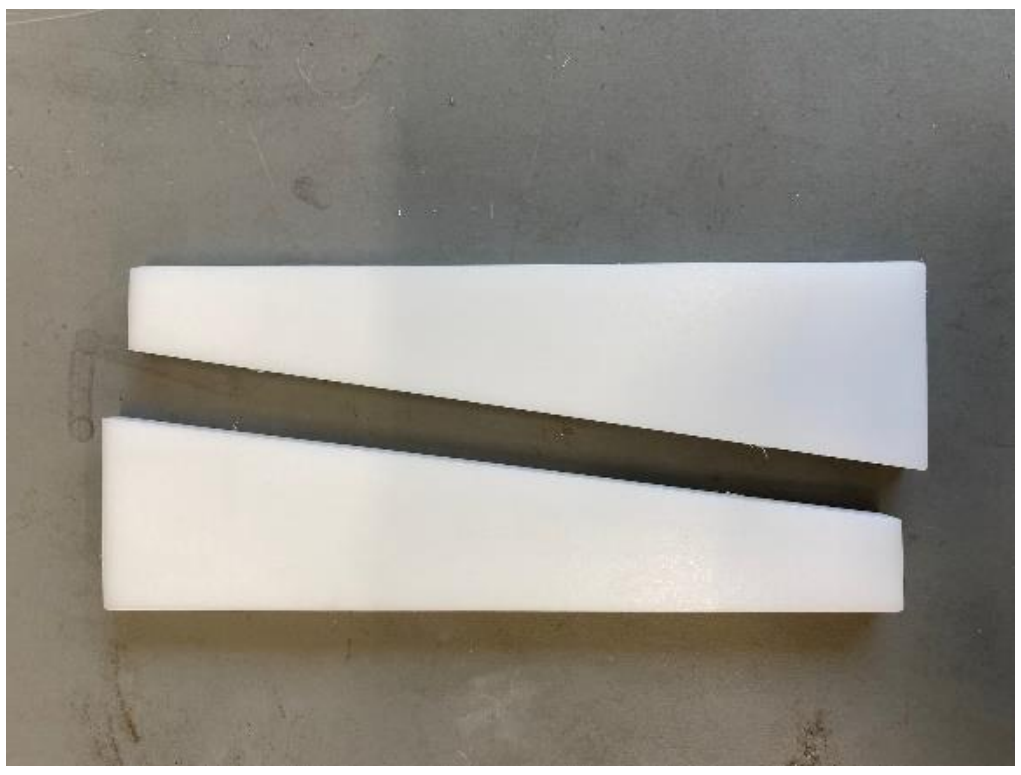

Drill and tap uprights as per drawing ' DRAW2 back board upright V1.0.'

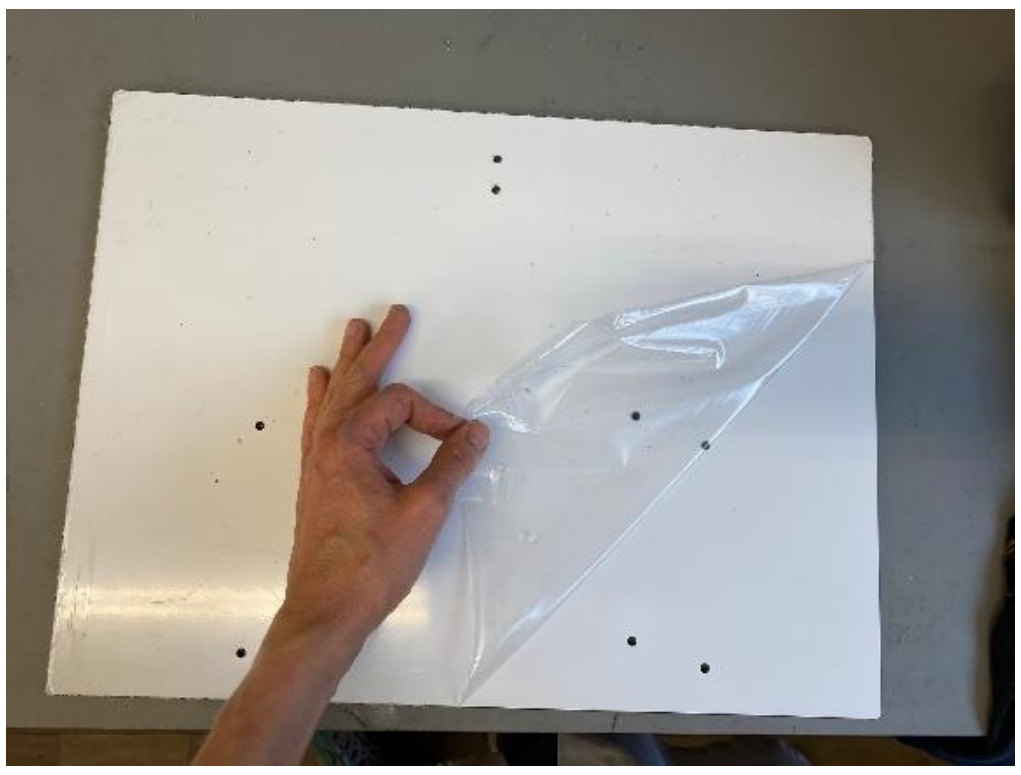

Remove protective film on back of board

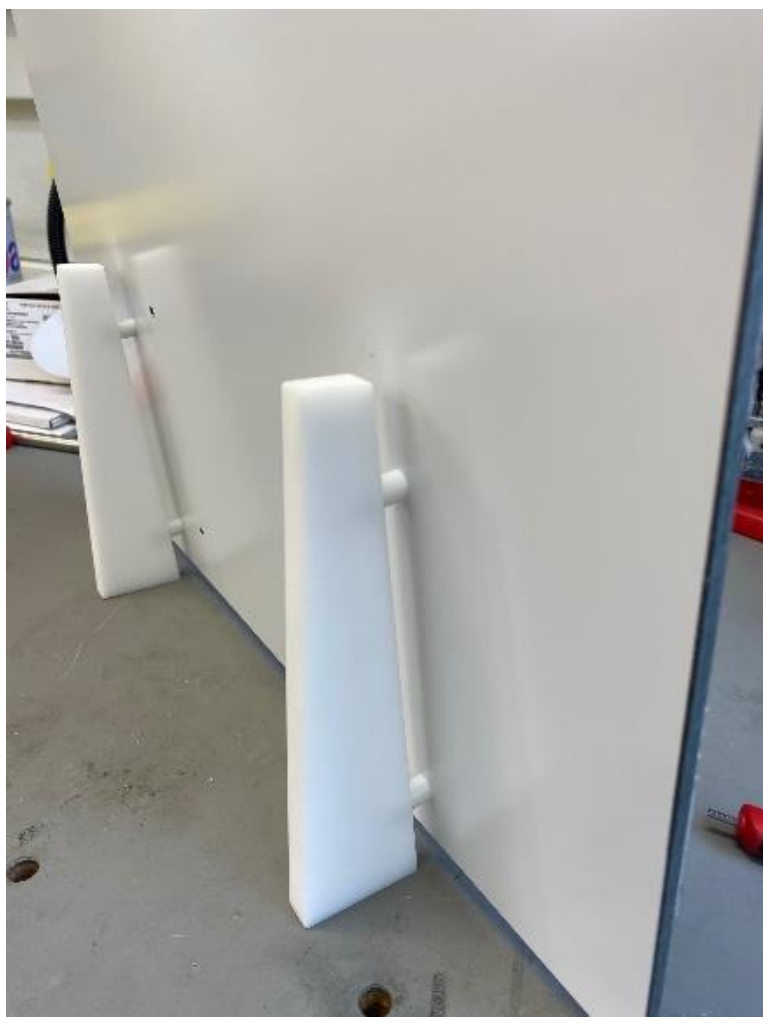

Attach back board to upright.

.

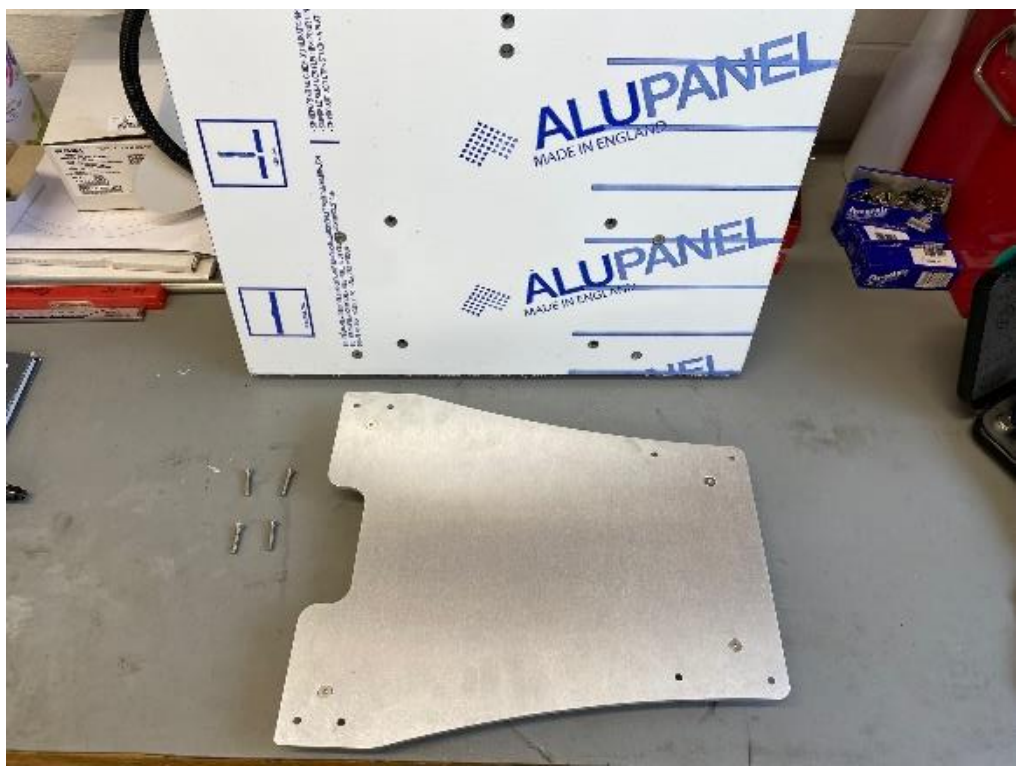

Attach base plate assembly to back board assembly.

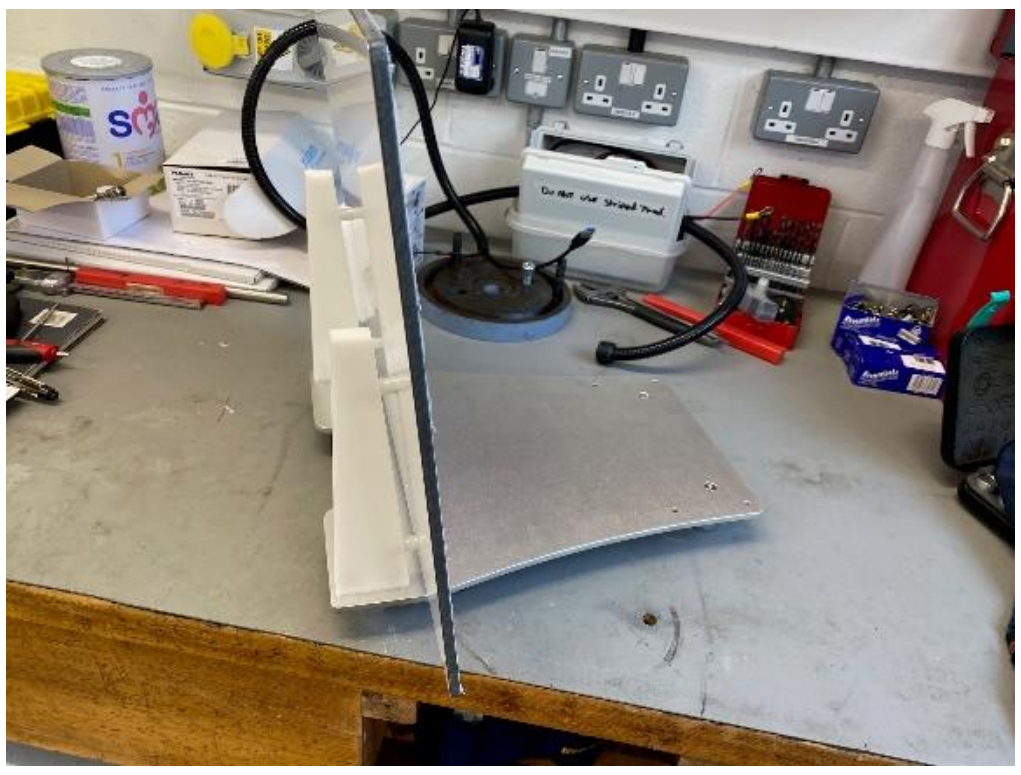

Assembly complete.

Electronics enclosure assembly

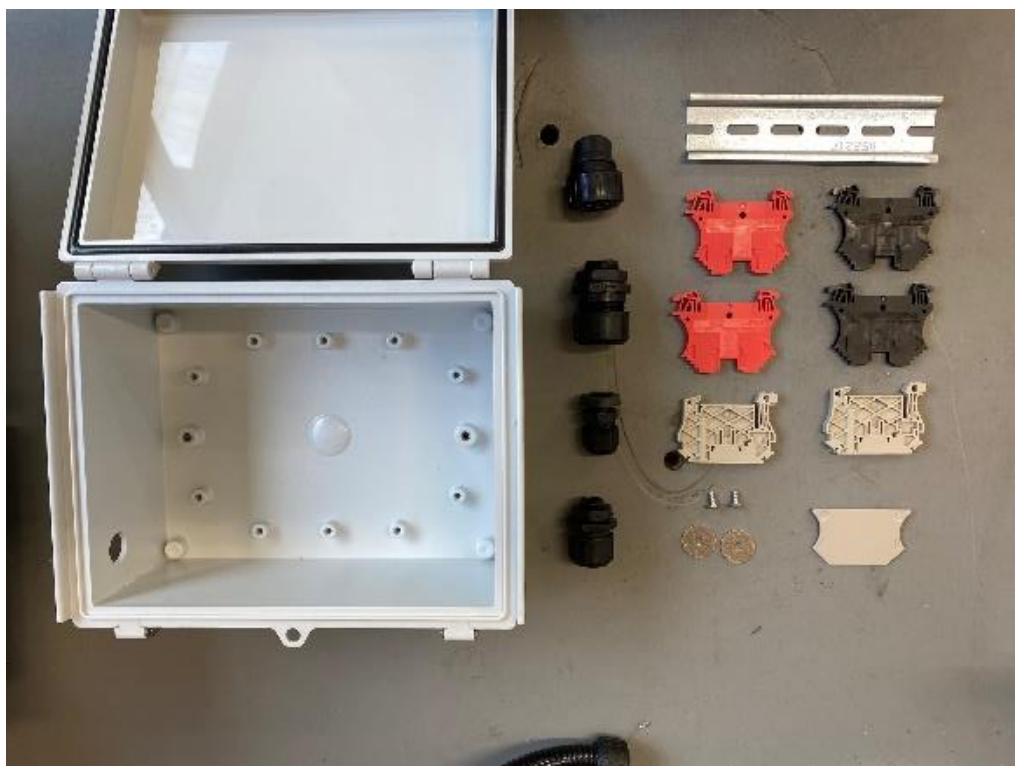

parts: 1 x drilled enclosure

1 x DIN rail
1 x 16 mm cable gland
1 x 21mm cable gland
1 x 21 – 16 mm reducer
1 x M16 skintop cable gland
2 x black terminal block

2 - 8 mm polyfix screw
2 – M4 penny washer
1 x end plate
2 x end stop
2 x red terminal block

Not shown

2 x jumper bars
1 x cable A
2 x no.4 pozi self-tapping screw
1 x 12v regulator

1 x timer relay
1 x cable B
2 x polyfix 4x10mm

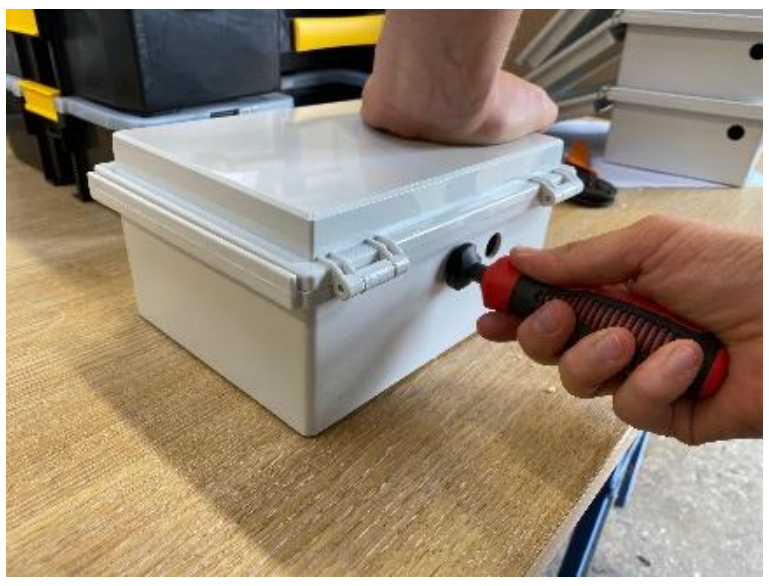

1- Deburr inside and outside of enclosure.

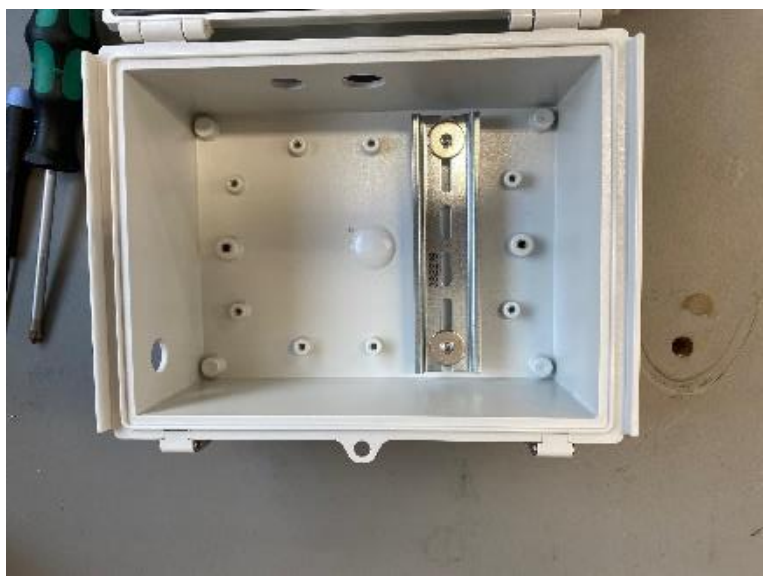

2- Install DIN rail using 2 x polyfix screws and washers.

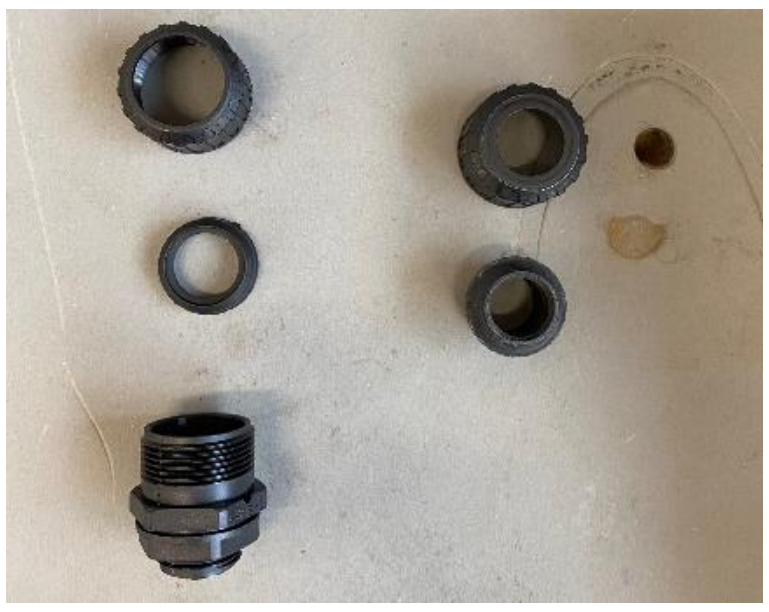

3 - Install the reducing gland to the 21mm gland.

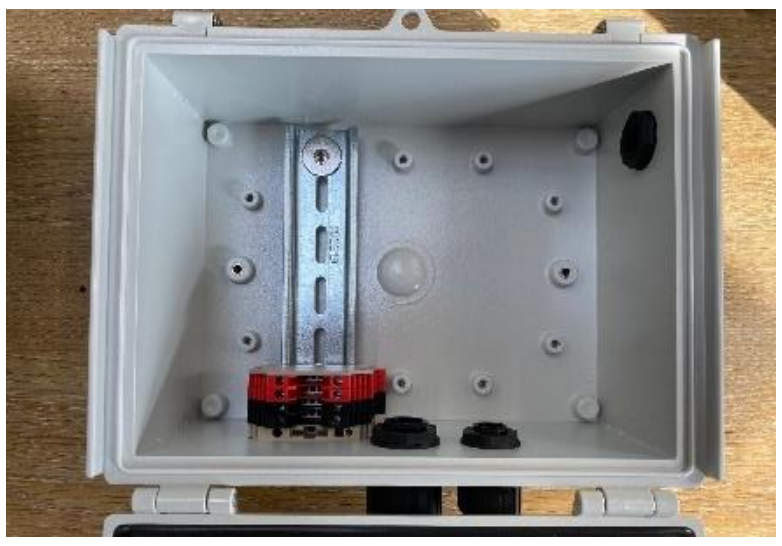

4 - Install cable glands, terminal bocks and end stops. Torque cable glands up, see *Figure 1*.

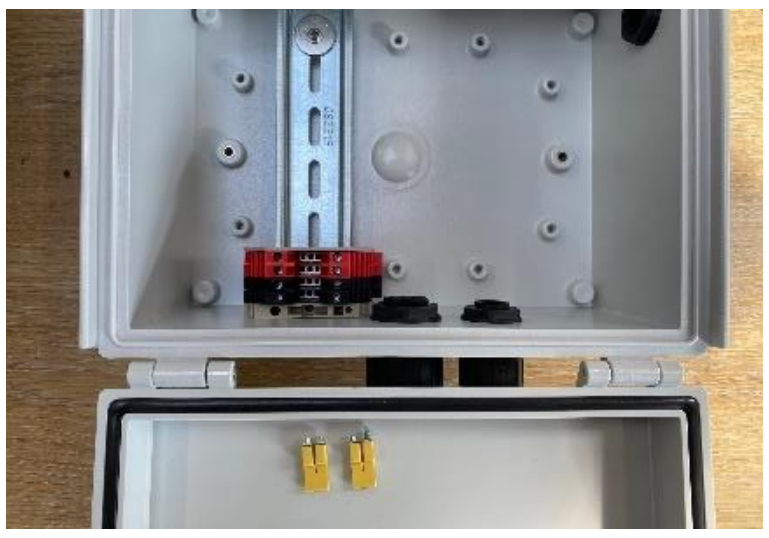

5 - Install 2 x jumper bars to the terminals

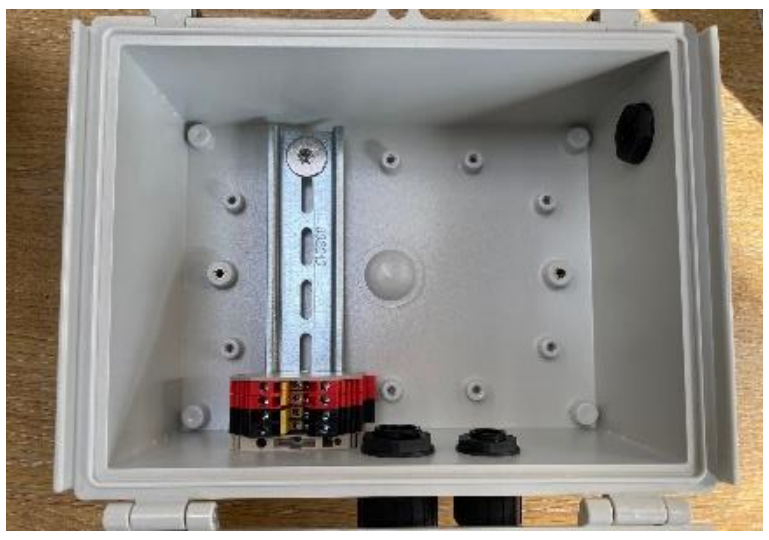

6 - Jumper bars installed.

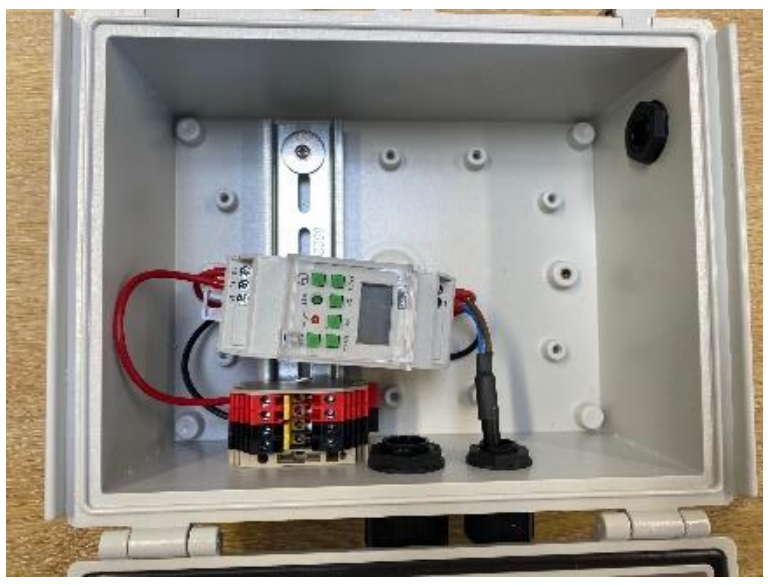

7 - Wire up the timer relay with cable A&B ensuring the cables are feed under the din rail rather than over.

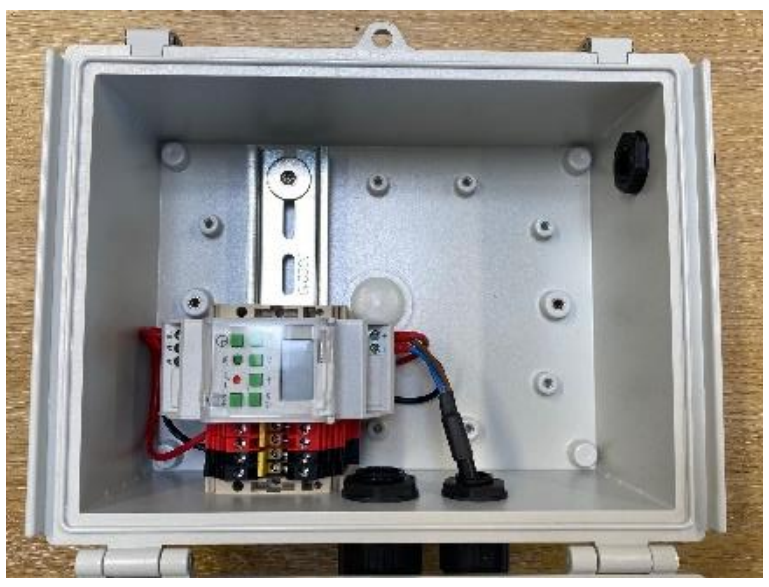

8 - Clip timer relay on to din rail ensuring same orientation as shown in picture.

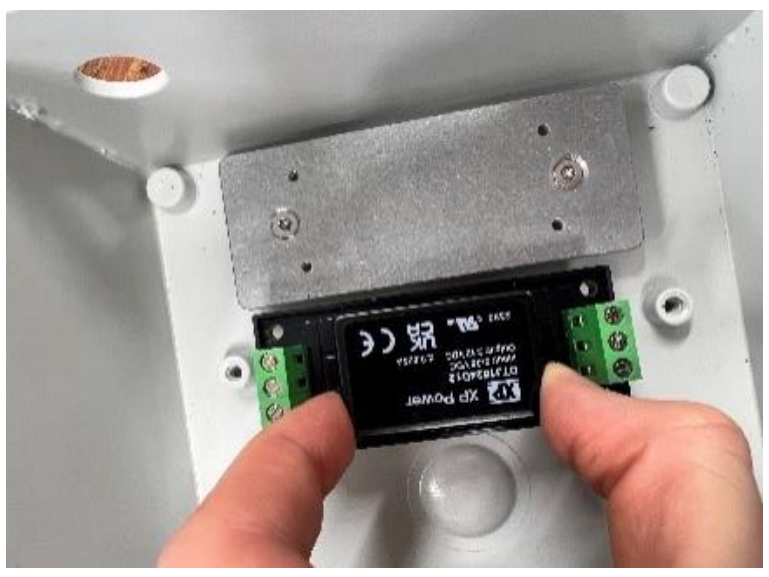

9 - Screw down the regulator mounting plate using 2 x polyfix 4x10mm

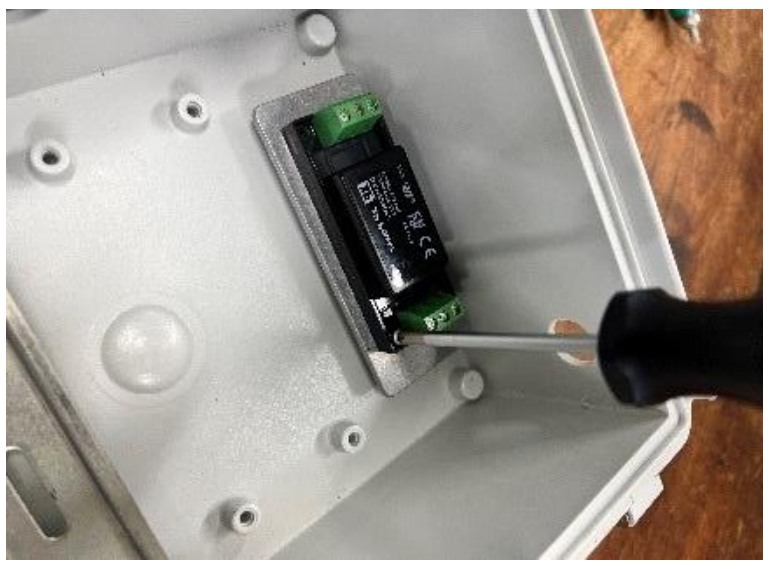

10 - Secure regulator plate using 2 x no.4 self tapping screw. Ensuring the power out is at the top of the box

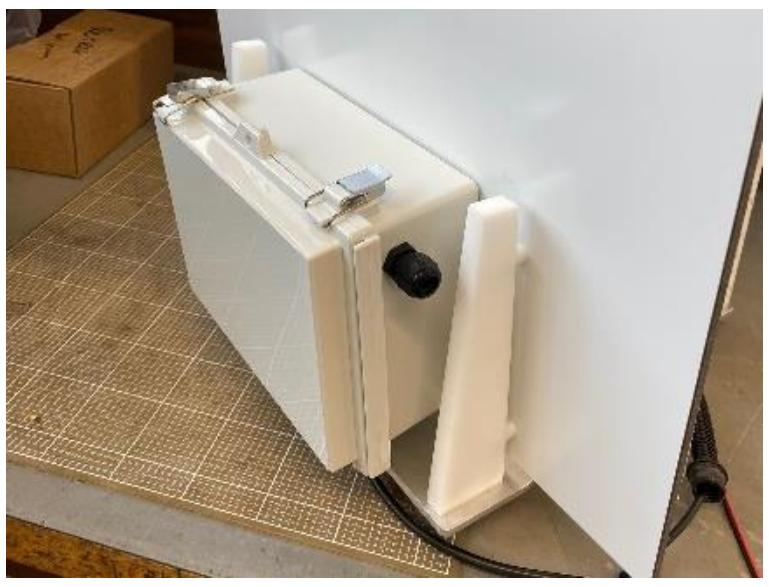

11 - Mount the electronics box using the 4 countersunk polyfix screws

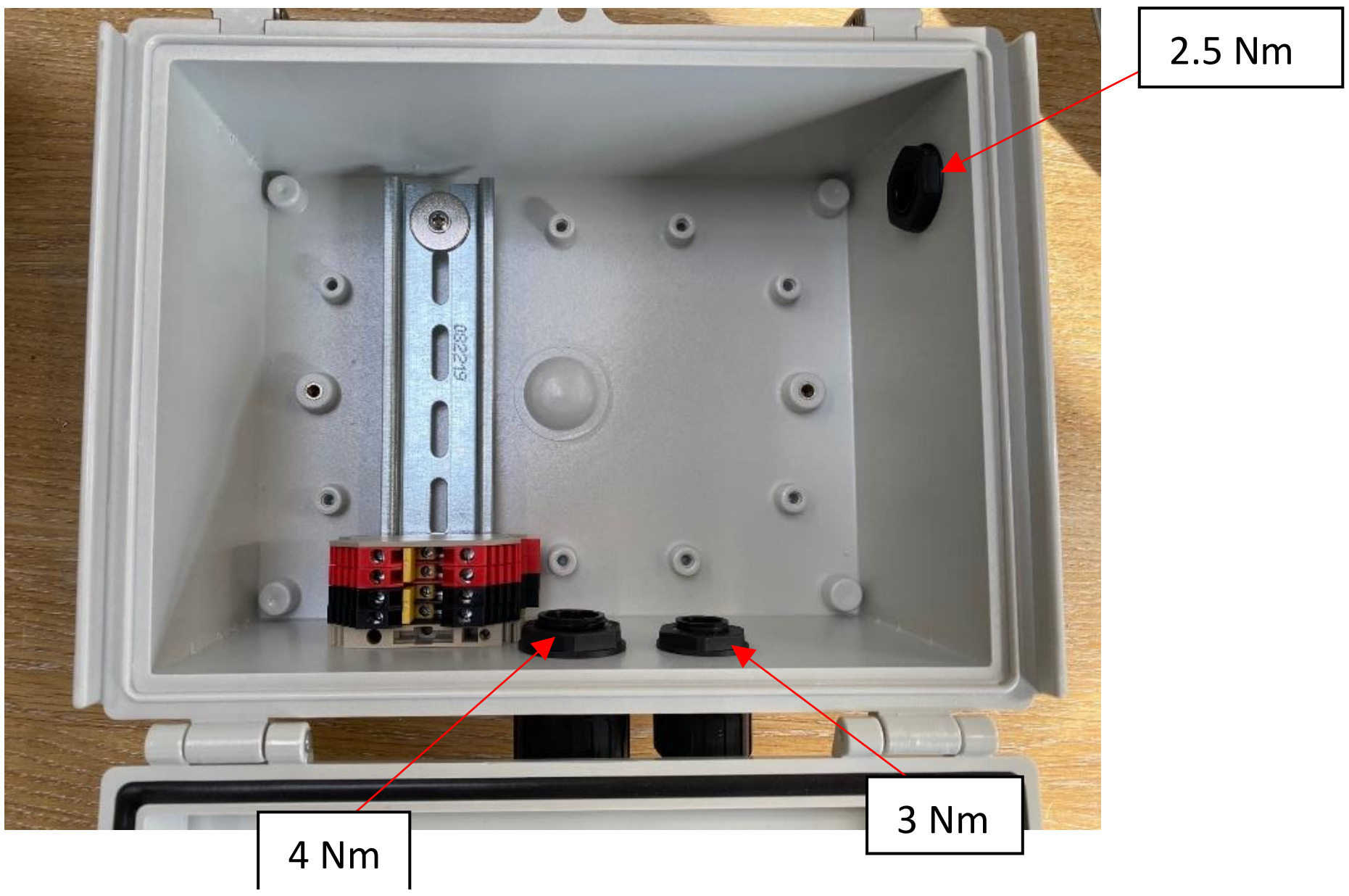

Figure 1 Torque settings

Light and web camera housing assembly and mounting

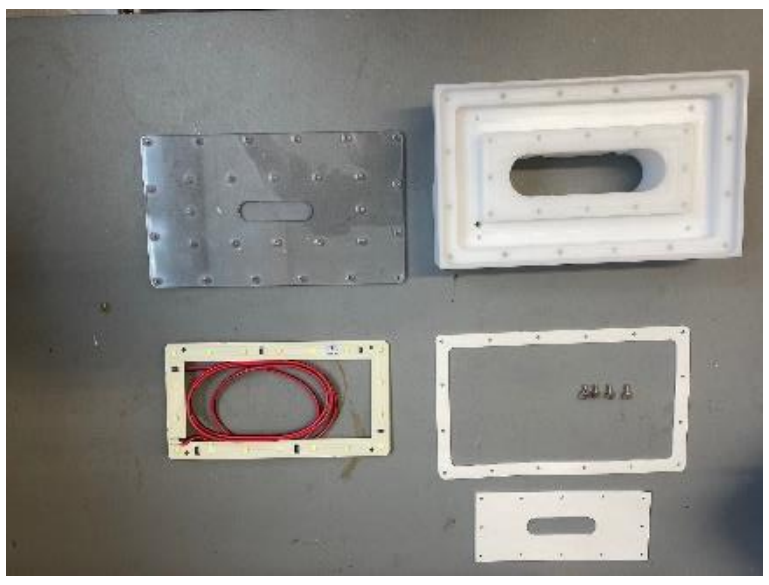

Not all parts shown

Parts :

- 1 x Front cover
- 1 x LED light
- 1 x Light &camera housing
- 1 x Outer gasket
- 1 x Inner gasket
- 4 x M3 standoff
- 4 x M3 nylon screws
- 1 x LHS leg
- 1 x RHS leg
- 1 x 21 – 16 reducing gland
- 1 x USB-C – USB-A cable
- 1 x 90 degree conduit gland
- 28 x M3 socket countersunk screws
- 1 x Camera
- 2 x Camera bracket fixing
- 4 x M4 socket pan head screws
- 1 x Back plate
- 1 x Back plate gasket
- 10 x M5 socket countersunk screws
- 4 x M6 socket countersunk screw
- 1 x M21 gland
- 750mm 16mm conduit
- 1 x 280mm conduit

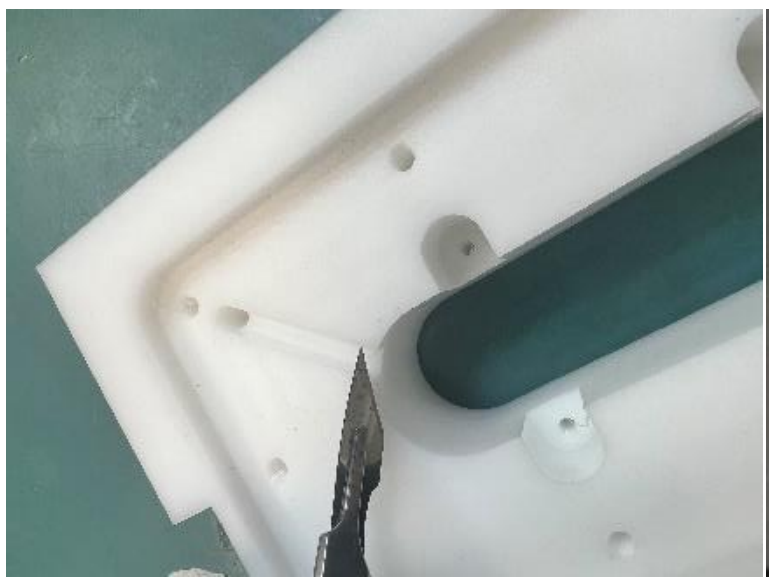

Deburr camera housing taking sharp edges off especially the cable channel.

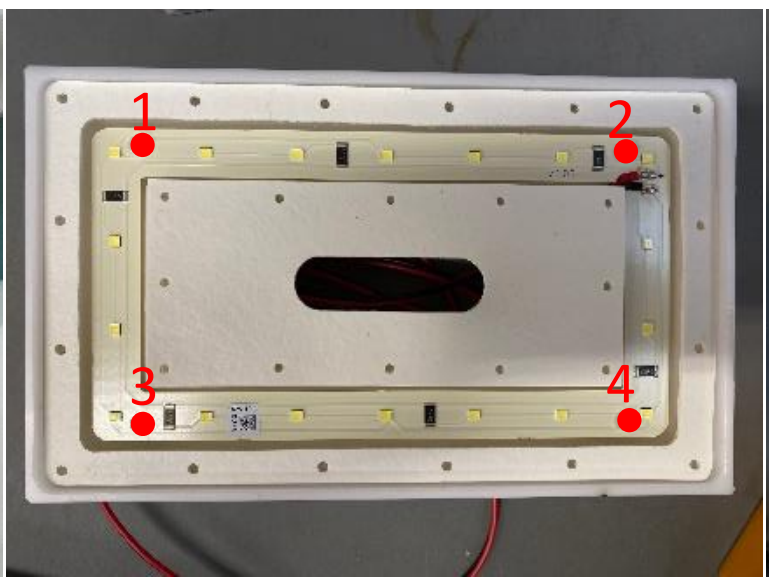

Install 4 x M3 stand off and secure PCB using the 4 x M3 nylon screws.

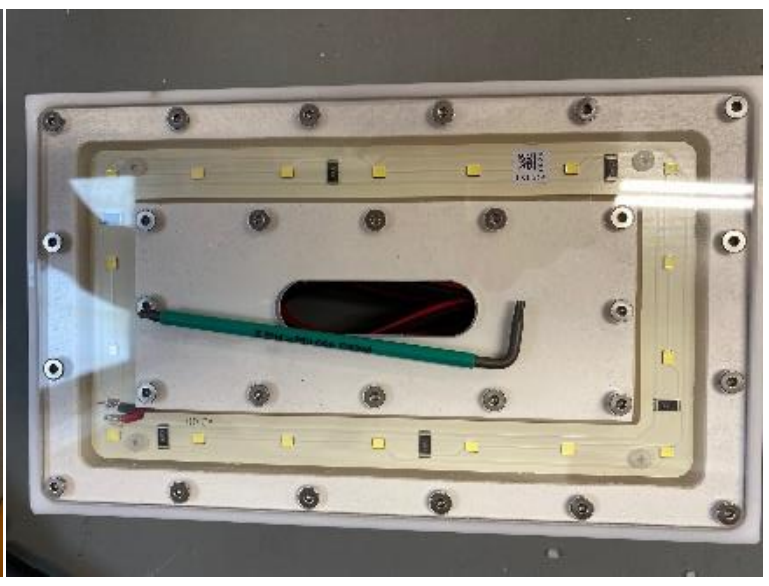

Place the inner and outer gasket then secure this using the front cover and 28 x M3 screws.

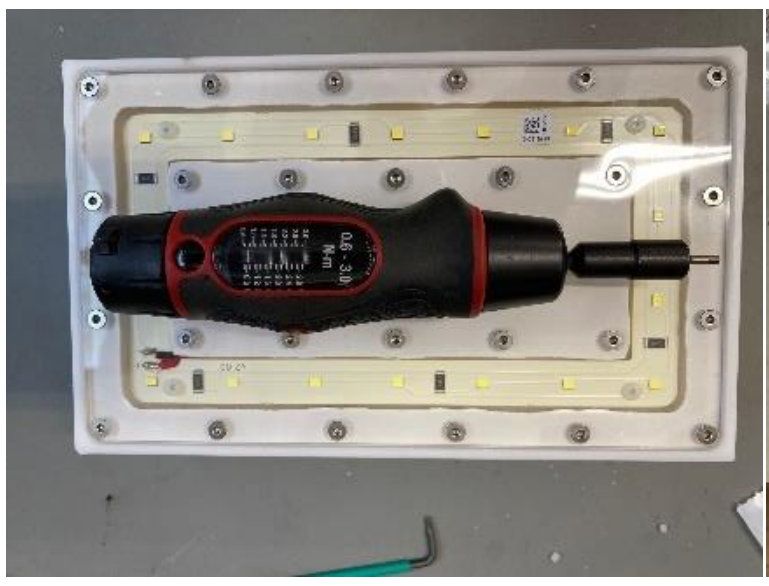

Torque the front cover screws up to 1 n/m.

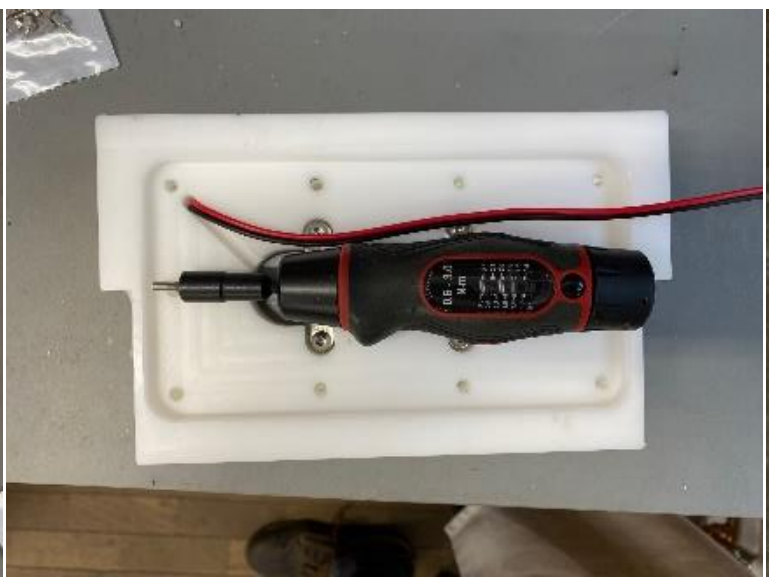

Place the camera in ensuring the USB is at the bottom and secure with 2 x camera brackets, 4 x M4 panhead screws torqued to 0.6 N/m.

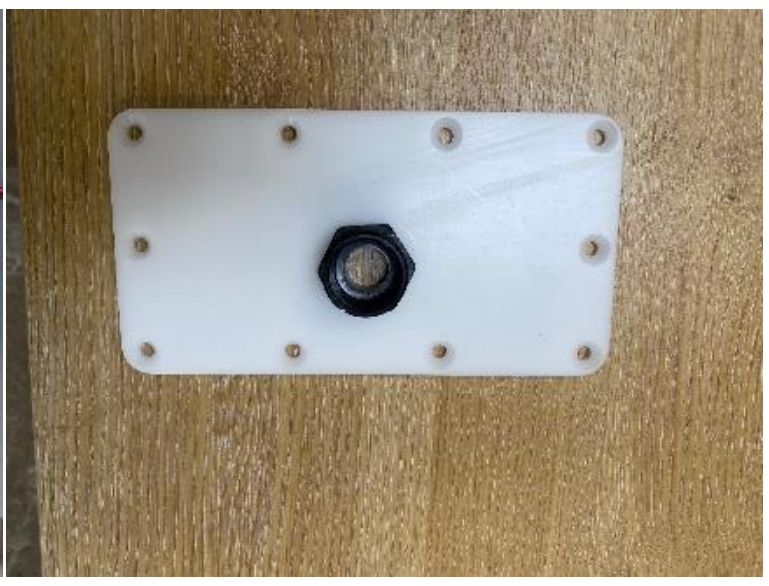

Install M21 gland torqued to 4 N/m.

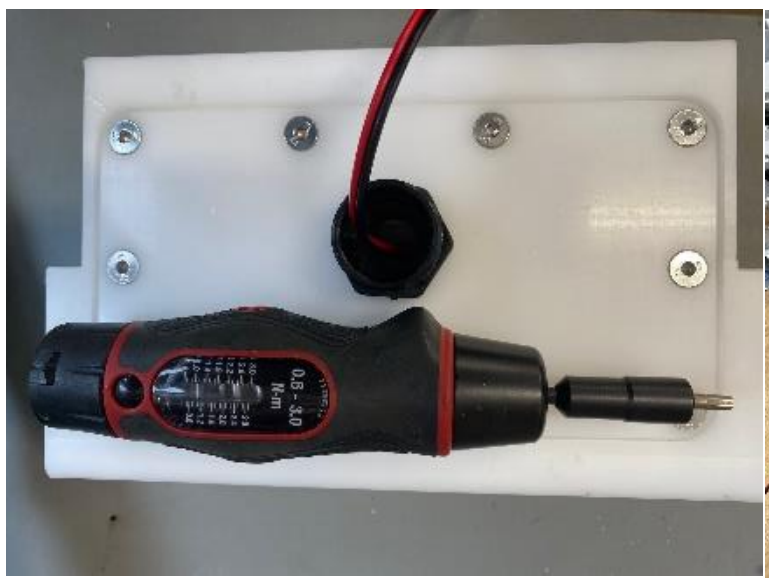

Place the backplate gasket and backplate on securing with 10 x M10 countersunk screws (torqued to 1 N/m) ensuring the cable is not trapped.

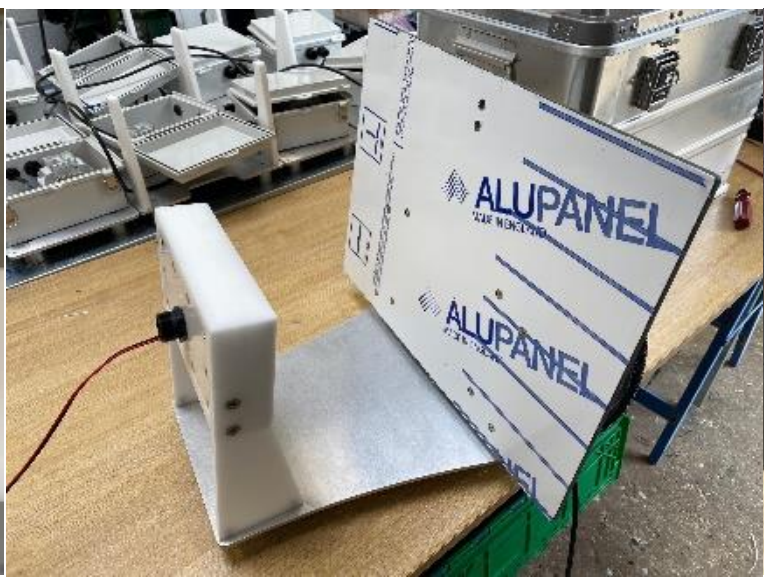

Mount the 2 x side legs using the 4 x M6 screws then mount to the base plate using 4 x M5 screws.

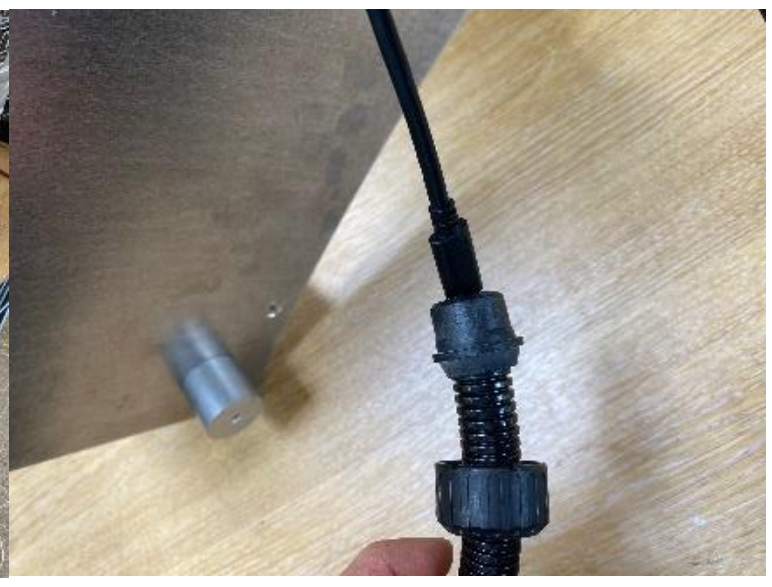

Feed the cable through to the conduit

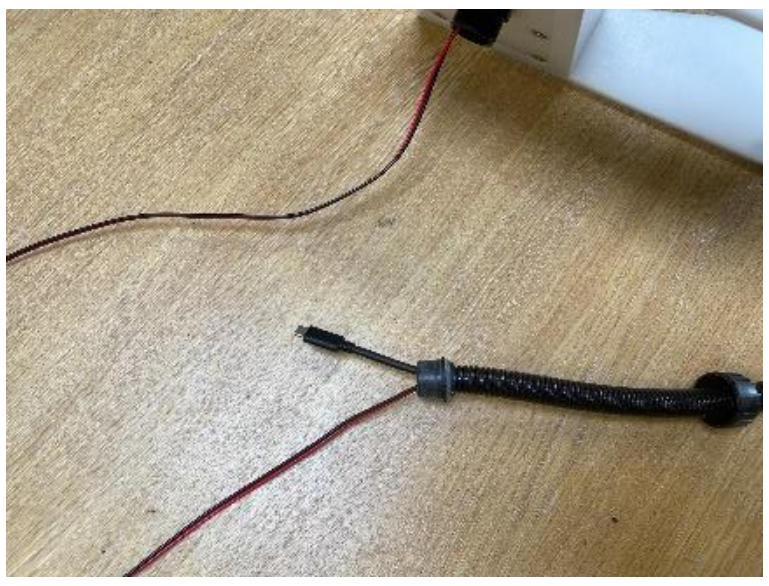
Then feed the power cable from the camera housing to the electronics box.

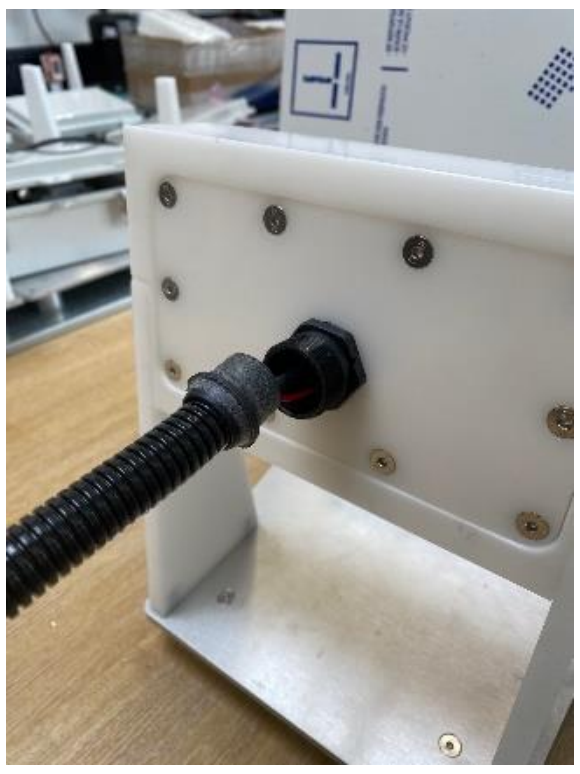
Insert the USB-C cable into the camera and tighten the gland hand tight.

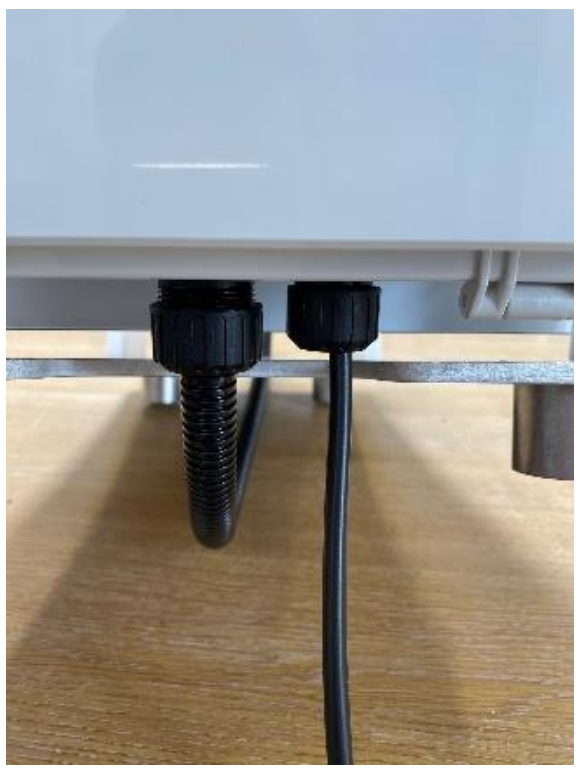
Tighten the large gland on the electronics enclosure hand tight.

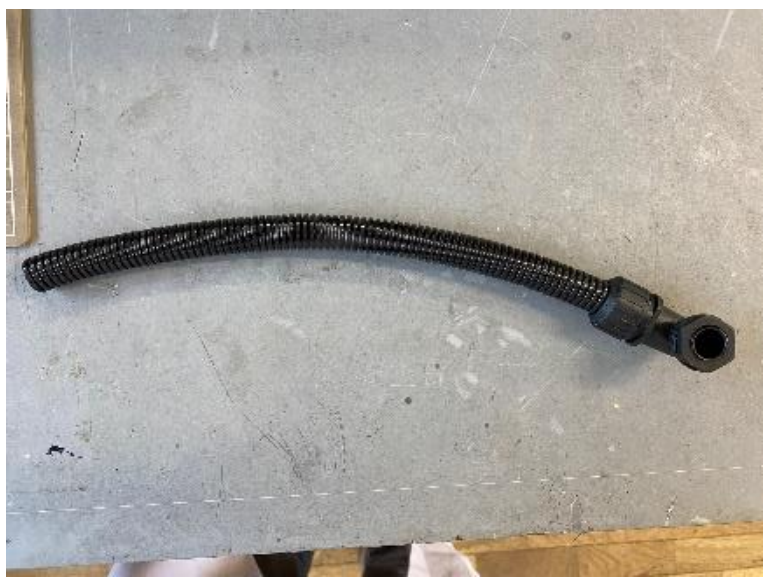
Attach the 90-degree conduit gland to the 280mm conduit.

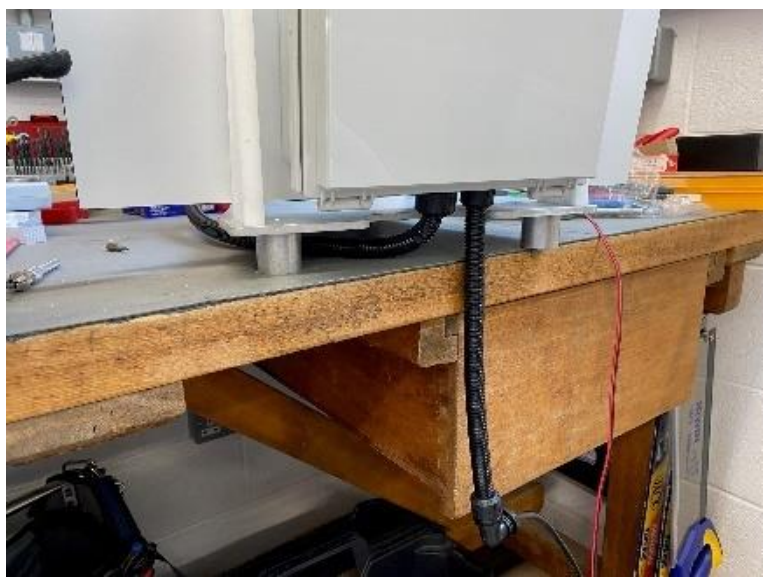
Attach the conduit to the electronics Enclosure.

Lepi LED

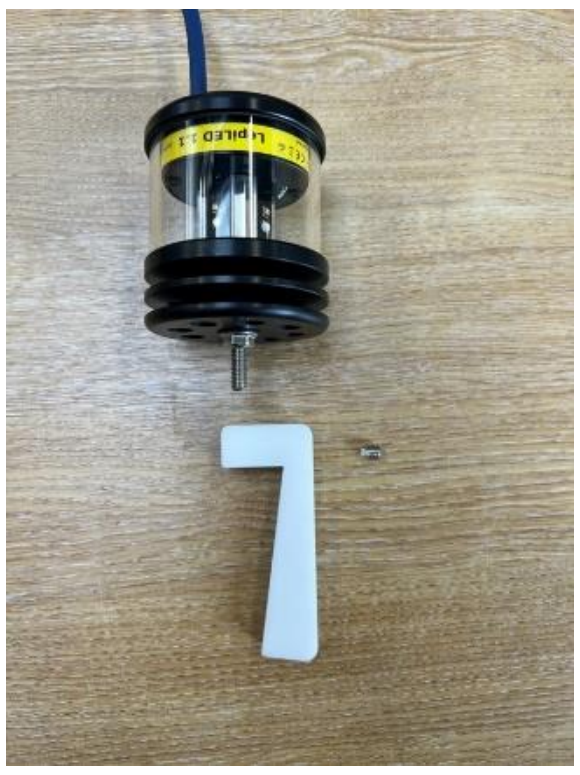

Parts: 1 x Lepiled (thread cut down)
1 x M6 grub screw
1 x Lepi led mount (see DRAW6 LEPI LED mount V1.0)

Not shown:
2 x M5 x 20 countersunk socket screws
1 x P clip (fixing hole opened out to 5mm)
1 x M5 panhead socket screw
1 x M5 washer

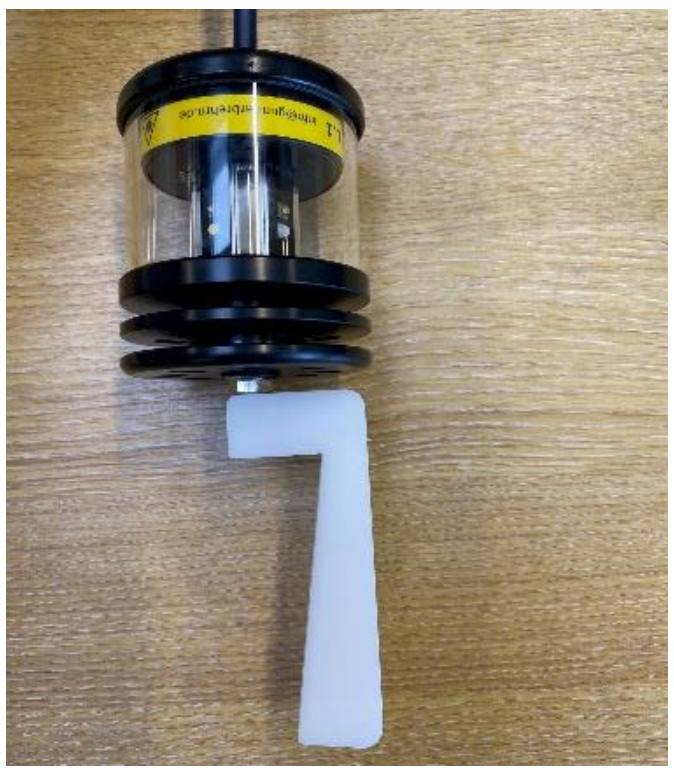

Mount the Lepi LED to the bracket and secure with grubscrew.

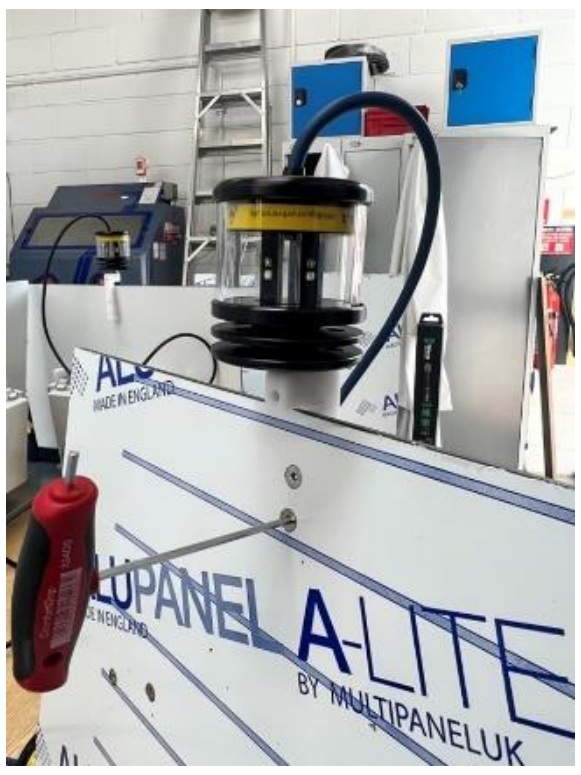

Mount the Lepi LED to the back board using 2 x M5 x 20mm screws.

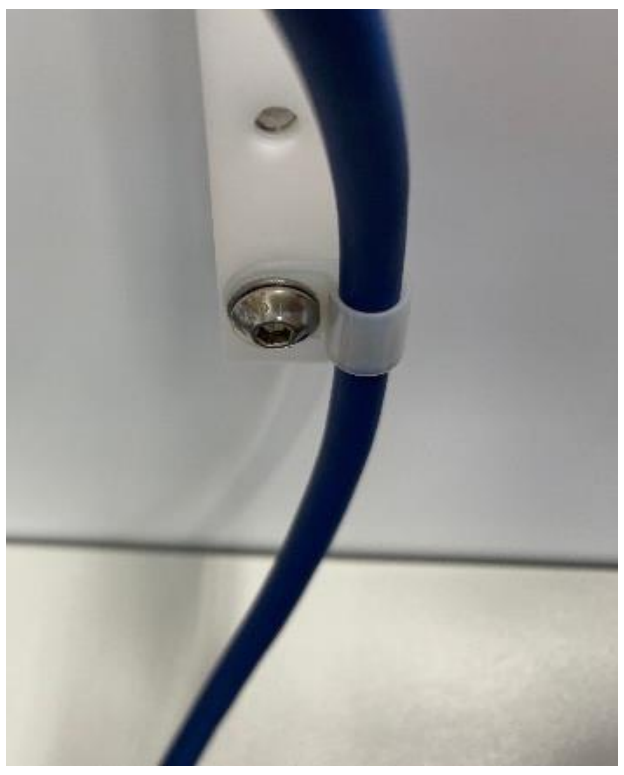

Secure the cable using the P-clip, m5 washer and M countersunk button screw.

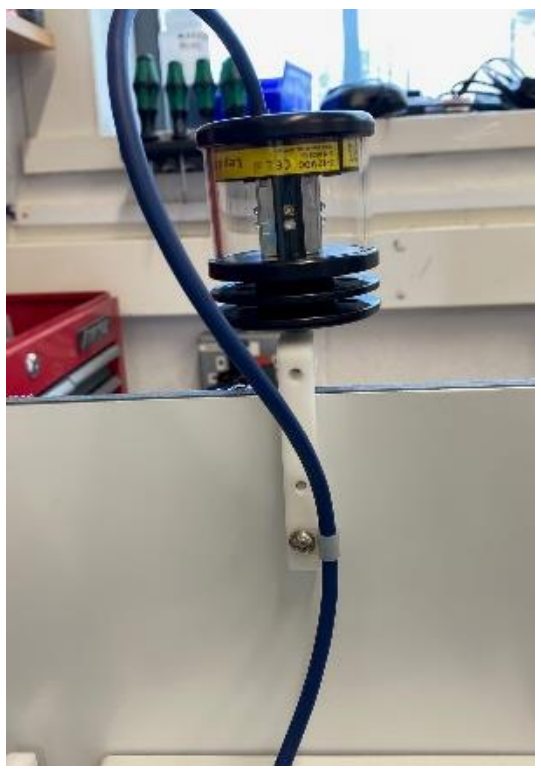

Lepi LED bracket mounted.

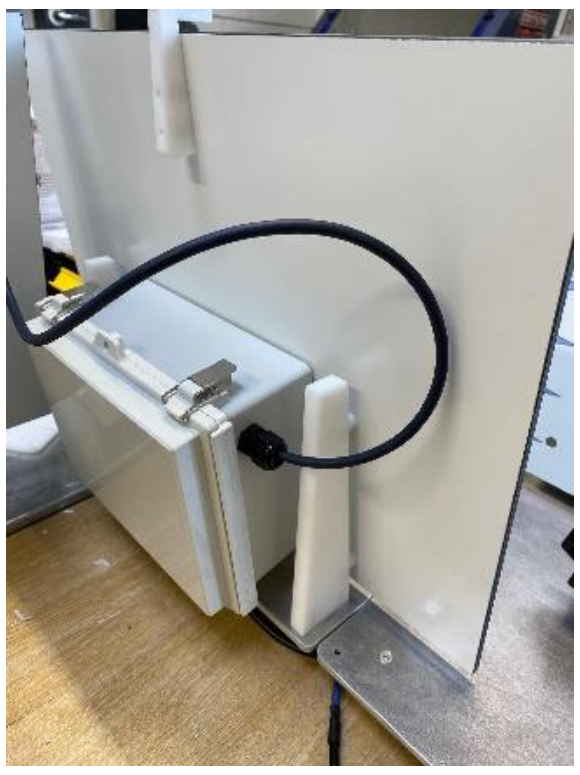

Feed the cable trough the gland on the electronics enclosure and tighten.
Wire up the cable according to electronics enclosure wiring.

12V – 5V stepdown

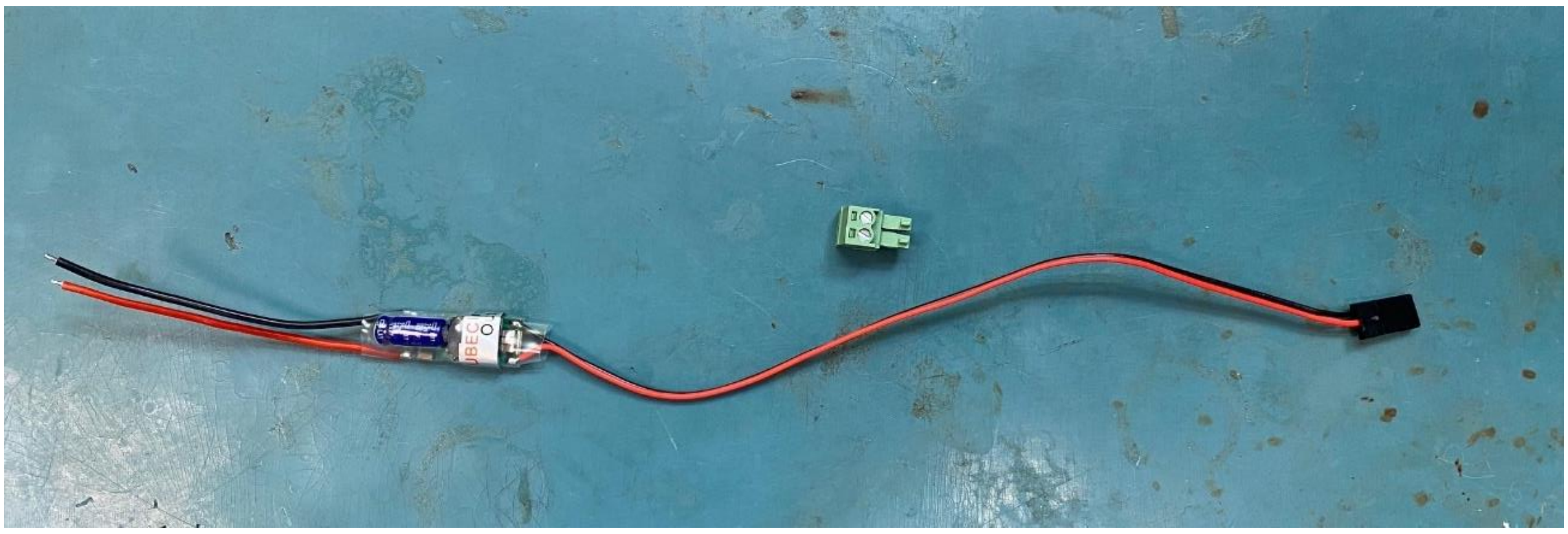

Parts required: 1 x Step down
1 x Terminal block (supplied with watchdog)

70mm long from end to PCB
Strip 10mm and tin ends

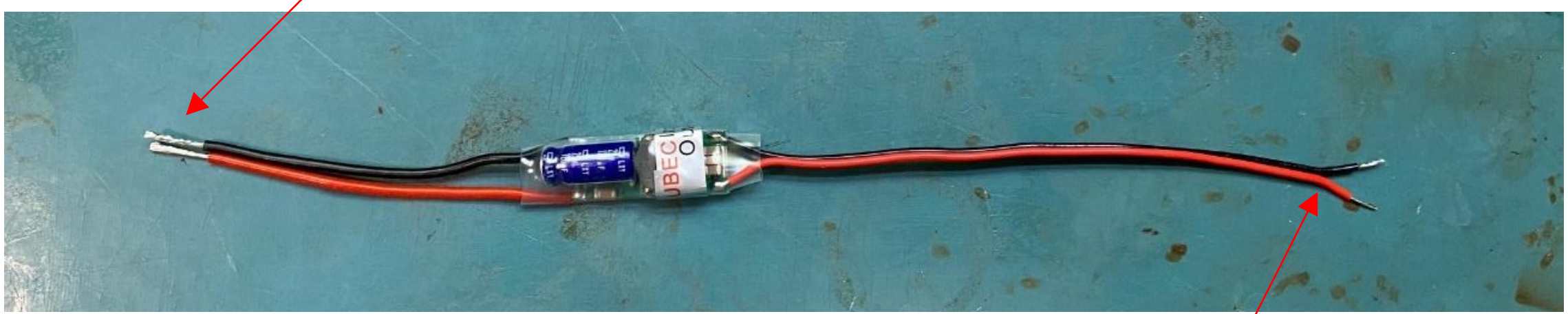

115mm long from end to PCB
strip 5mm and tin ends

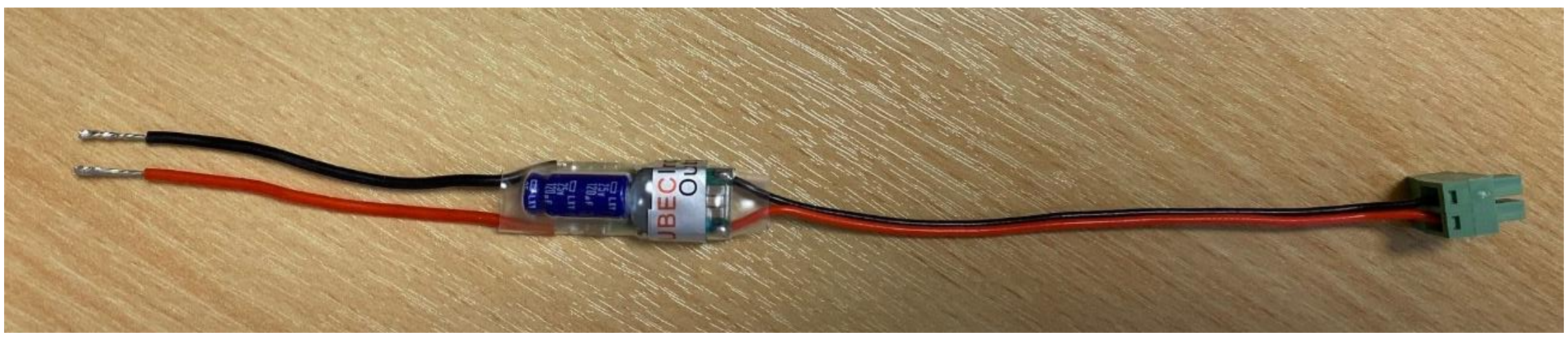

Screw end terminal on ensuring the connector is wired correctly for the watchdog.

Raspberry Pi installation

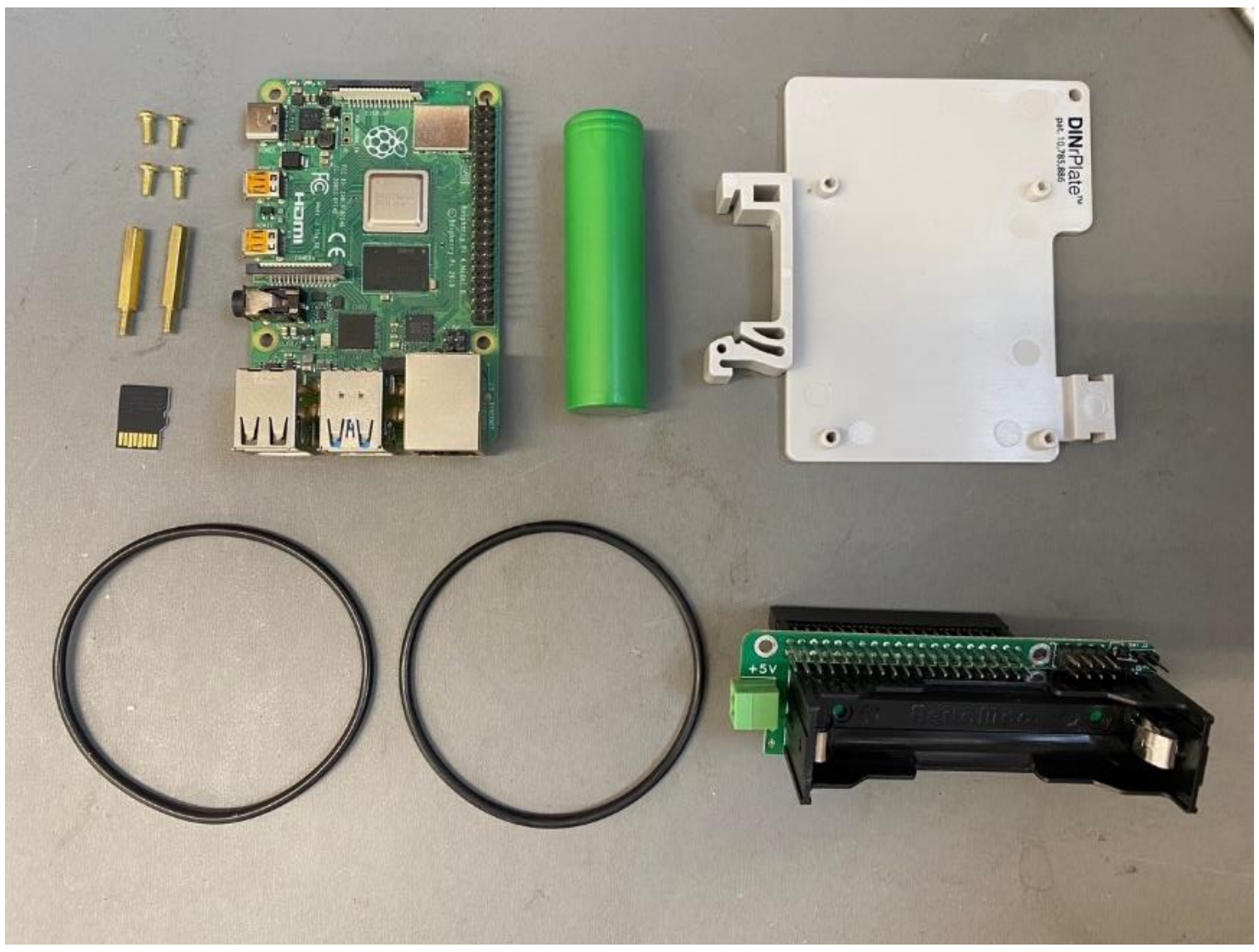

Parts:

1 x Raspberry Pi	4 x M2.5 x 5 pan head screw
1 x DINrPlate	2 x M2.5 x 25mm standoff male – female 20mm body length
2 x O-ring	1 x Super watchdog
1 x SD card	1 x Battery (18650 Lithium-ion Rechargeable Cell - 2500mAh 3.7V)

Not shown

1 x USB-C to USB-A 3.1	1 x Header pin extender
1 x RTC	2 x Bootlace ferrules
1 x SSD	1 x Heat sinks

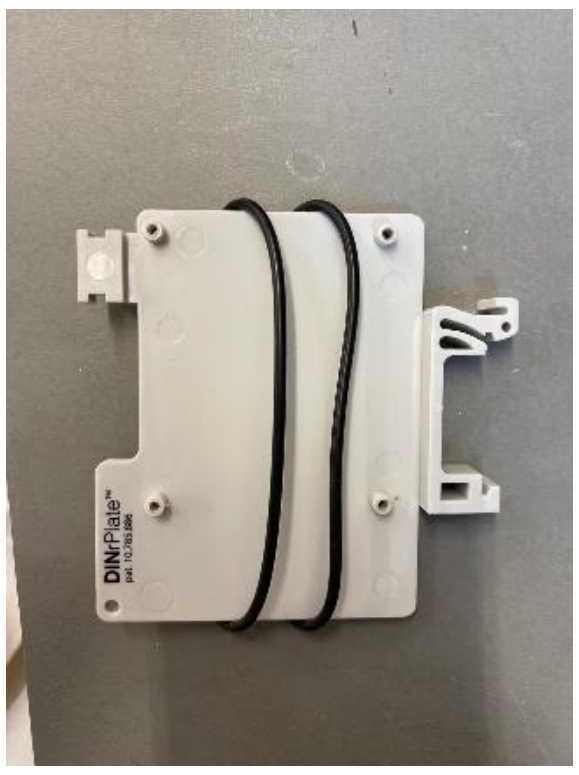

Place two O-rings around DINr Plate.

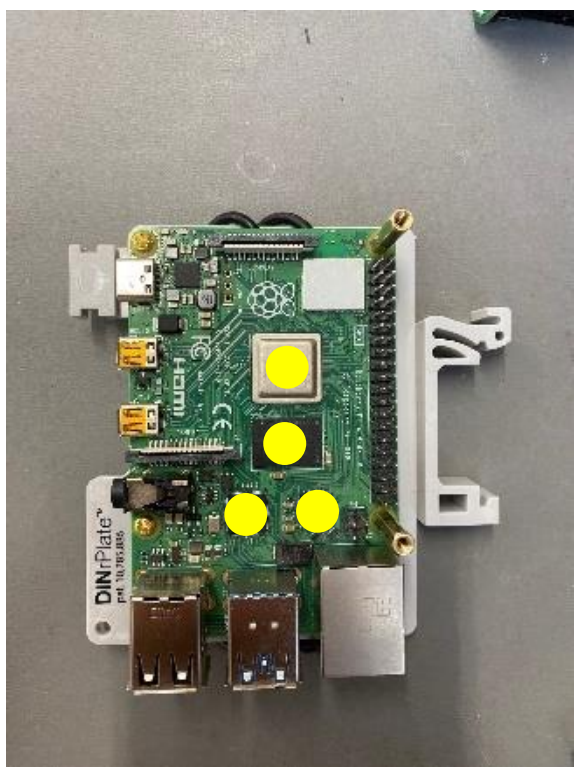

Mount the Pi using 2 x stand offs and 2 x M2.5 screws.
Then attach the heat sinks.
the position of the heat sinks are shown in yellow.

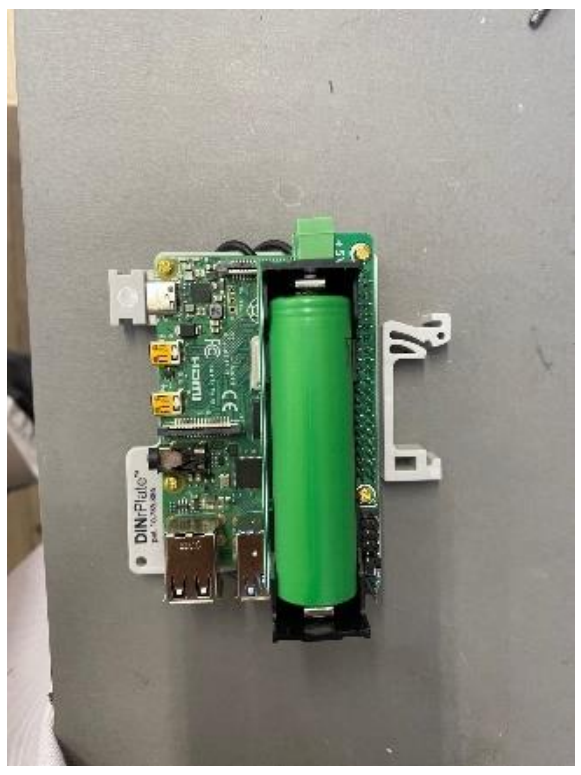

Mount the watch dog and secure with 2 x M2.5 screws install the battery ensuring the correct orientation.

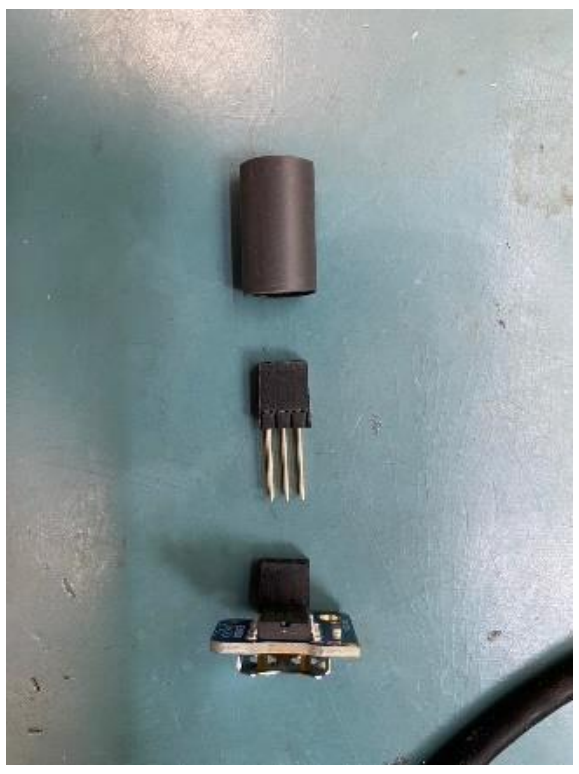

Mount the extender to the RTC and then use heat shrink to hold place.

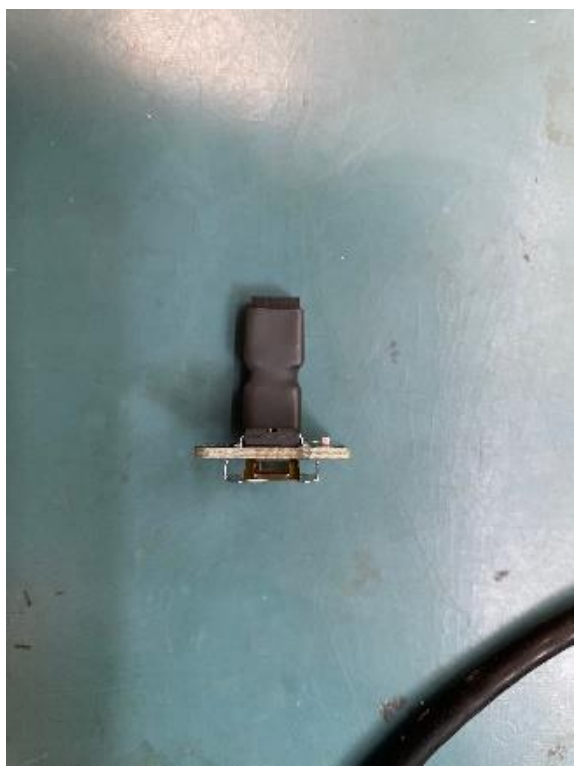

Heat shrink securing extender to RTC.

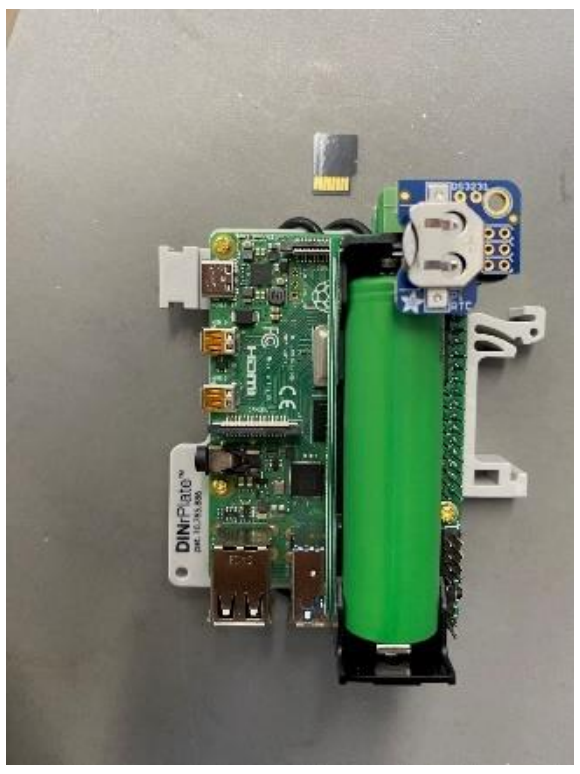

Mount RTC to GPIO Pins 1-6, install batteries and insert SD card with OS image loaded

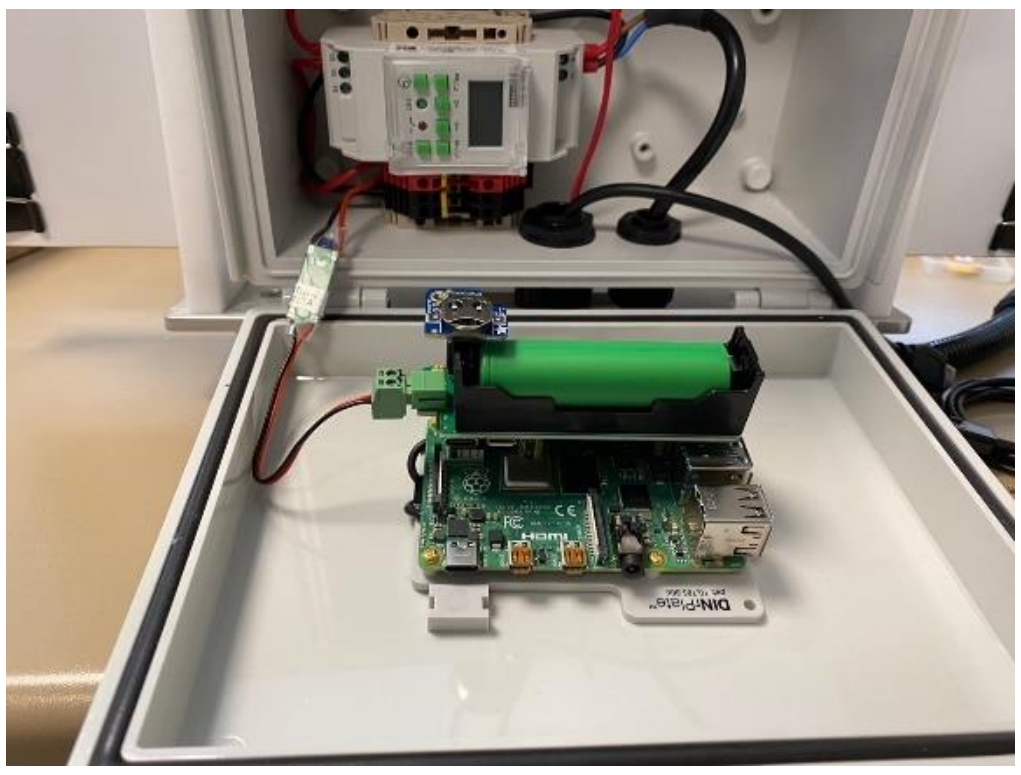

Plug watch dog into step down.

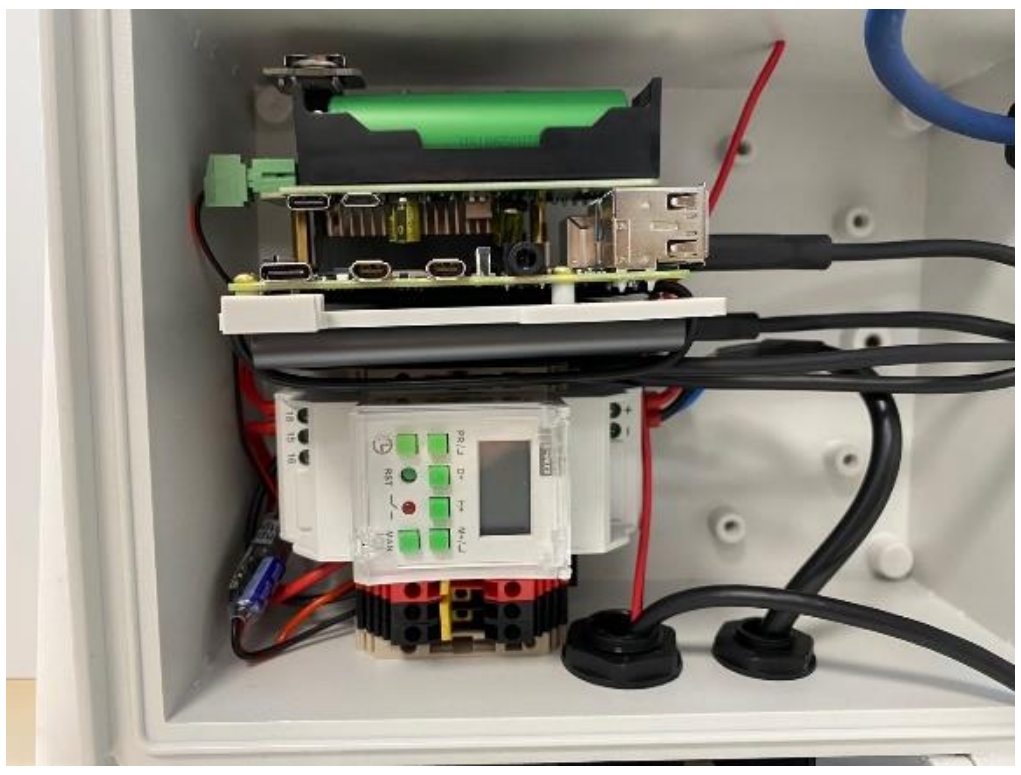

Secure the DINr plate to din rail. Then secure SSD in place with O-rings. Plug the SSD into the bottom port of the raspberry Pi

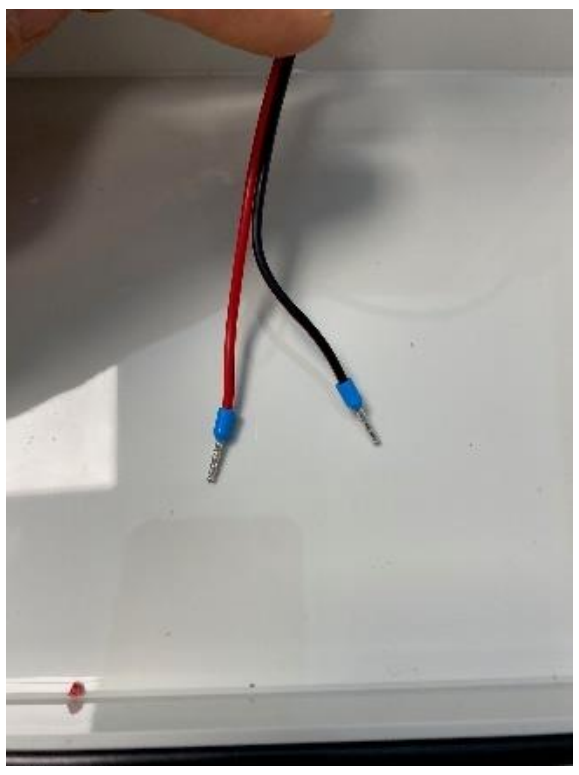

Strip the LED light ring cable and crimp 2 bootlace ferrules onto the end of the wire. ensuring the wire is flush with the end of the ferrule.

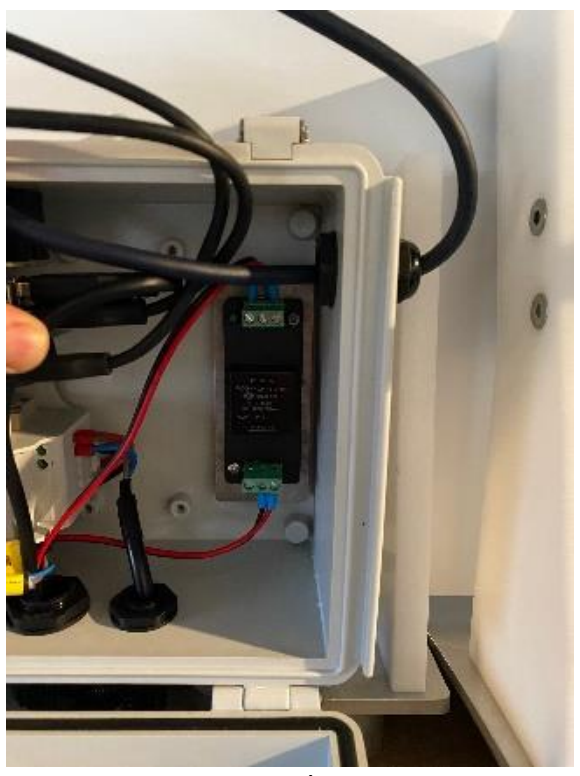

wire up regulator as per figure 2

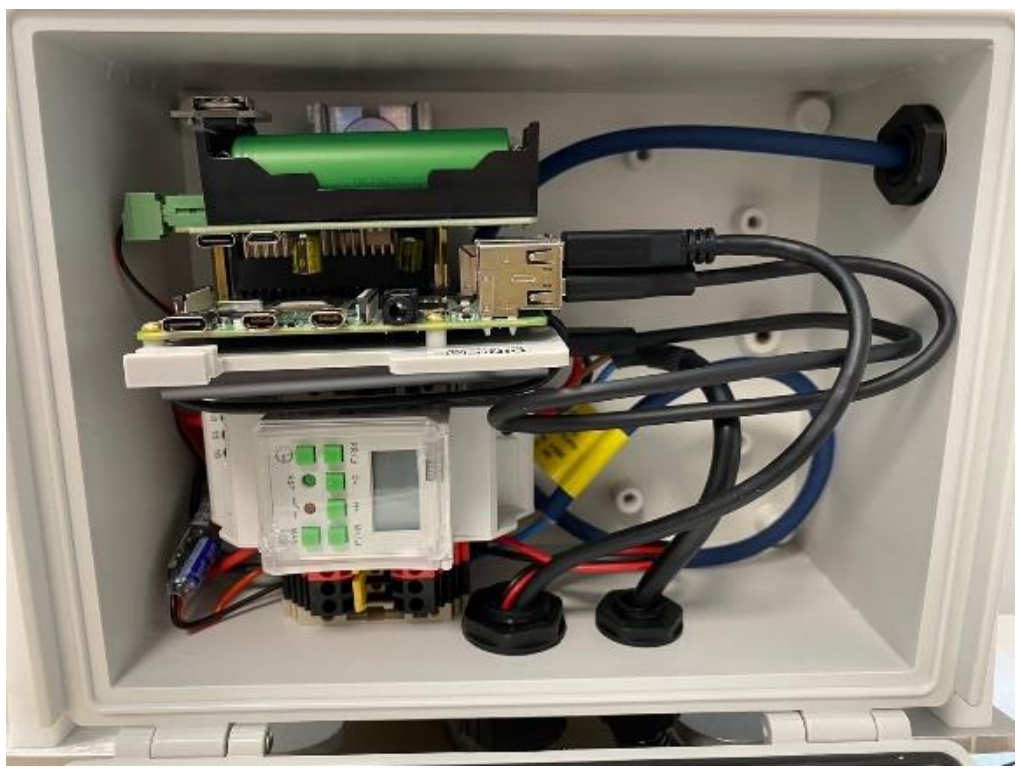

Wire up enclosure are per figure 3 and connect camera USB to top port on raspberry Pi.

Cables

Cable A

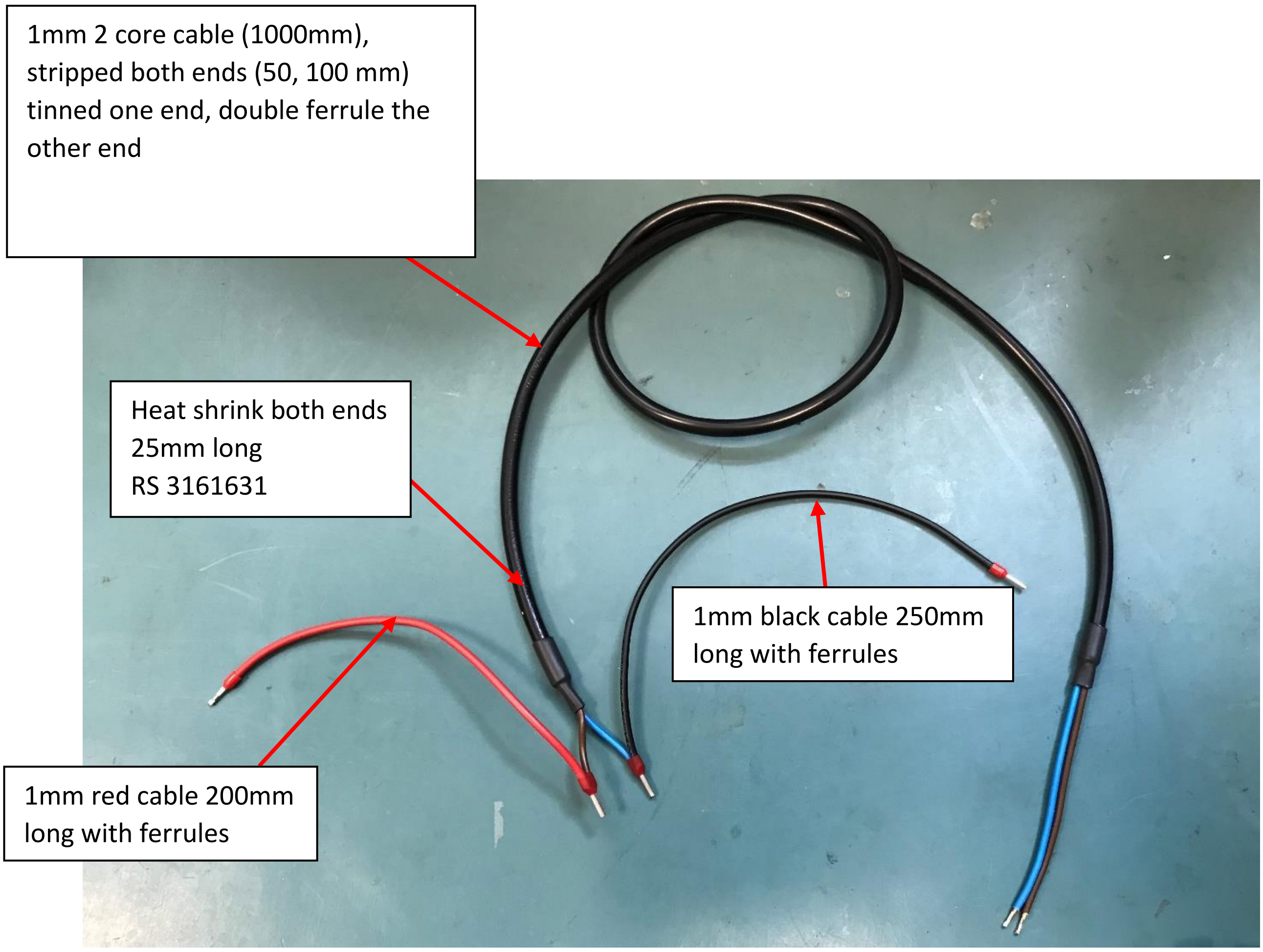

Ensure cables are flush with end of ferrules and cables

Cable B

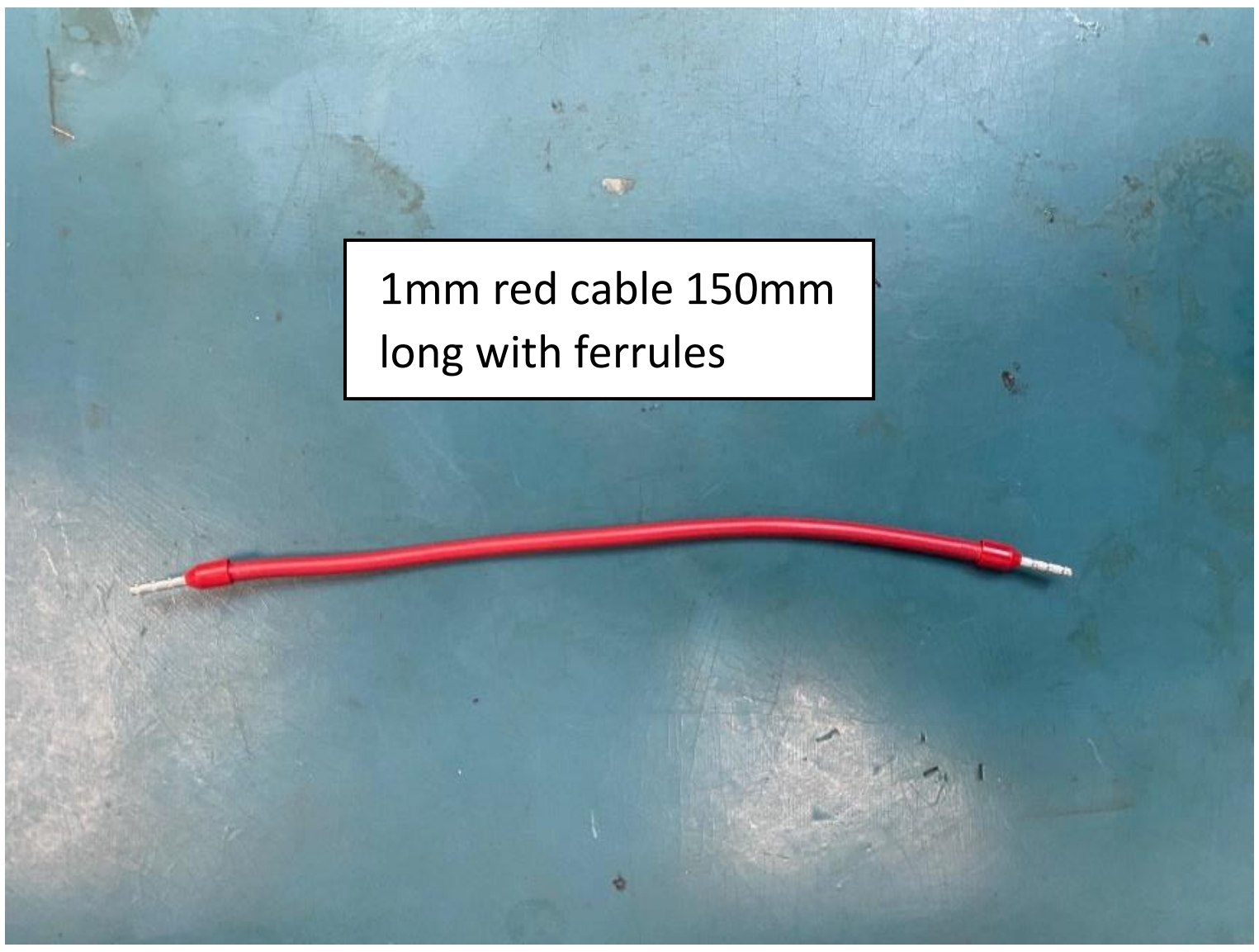

Ensure cables are flush with end of ferrules and cables

Cable C

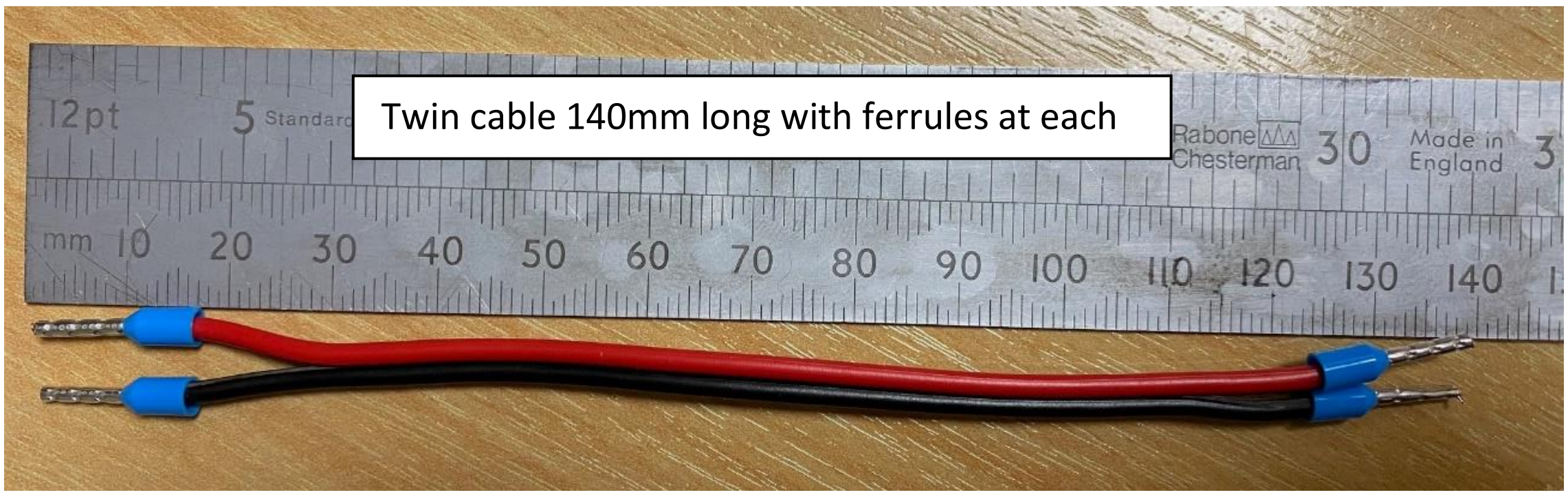

Electronics enclosure connections

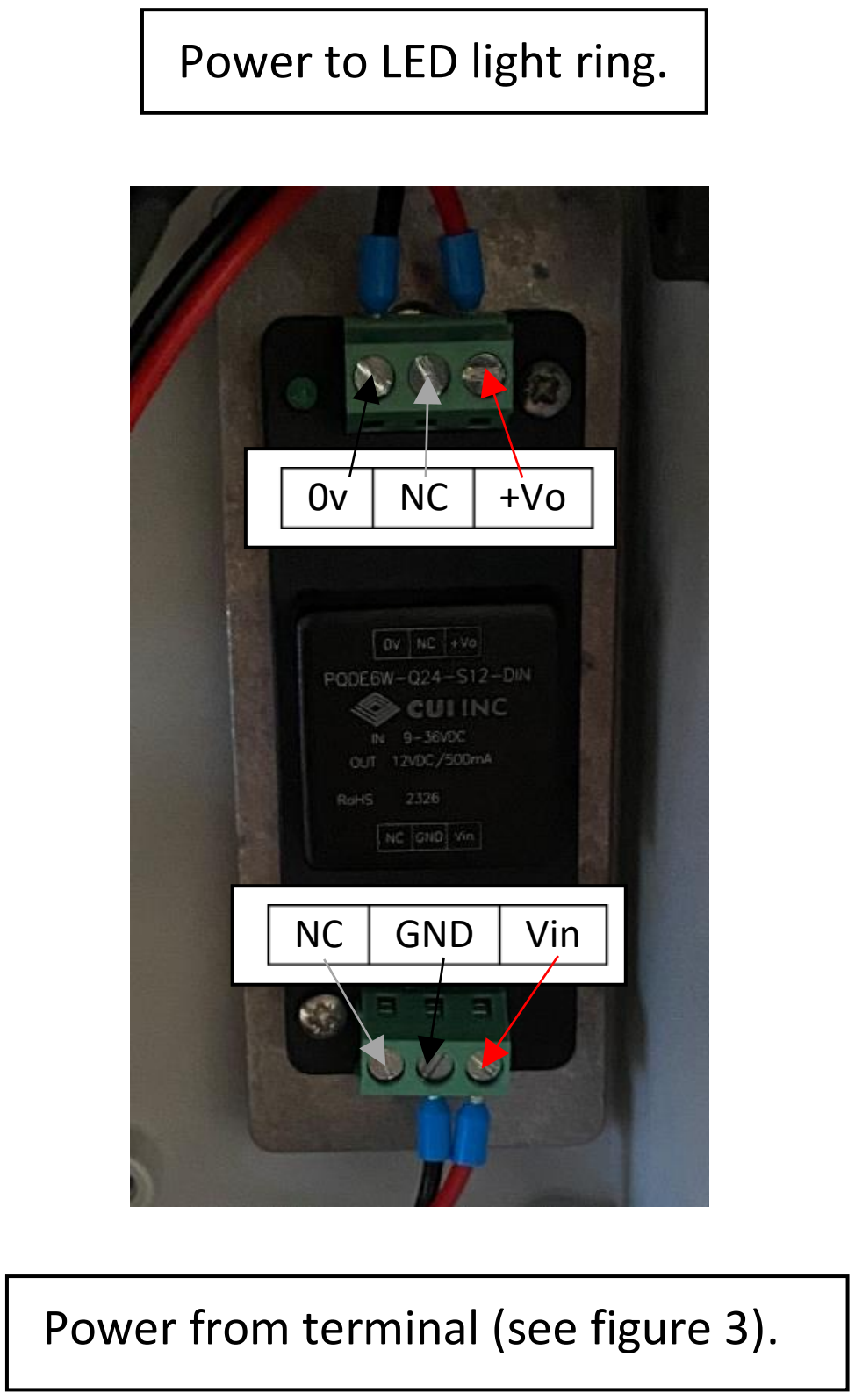

Figure 2. 12v regulator connections

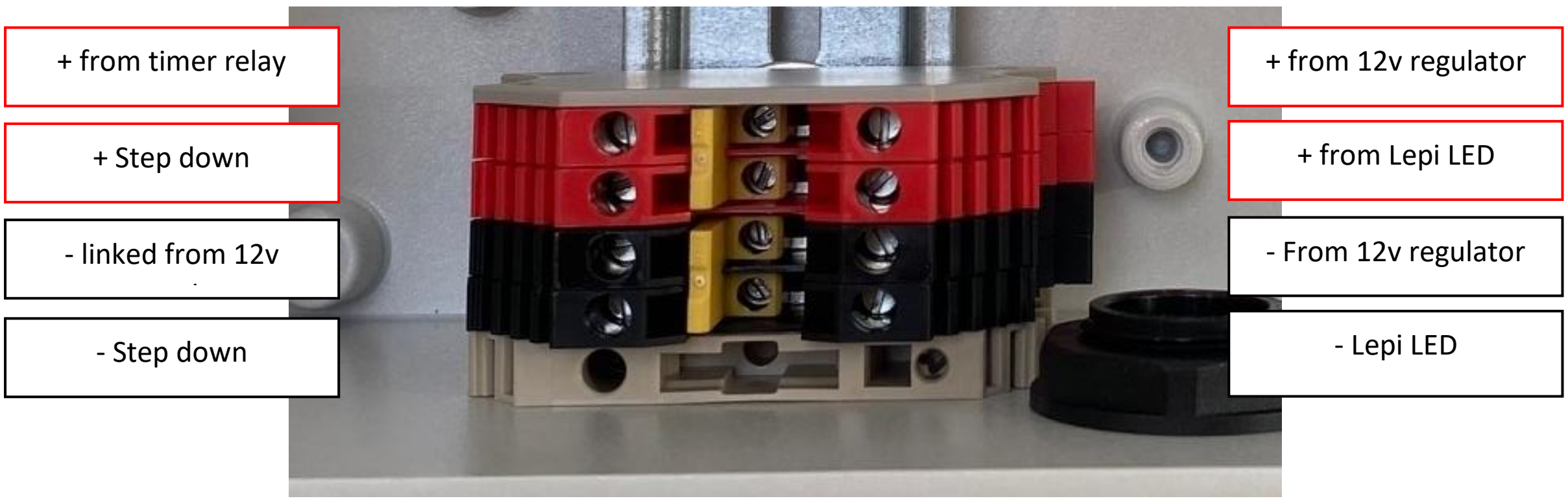

Figure 3 electronic enclosure connection

Battery box

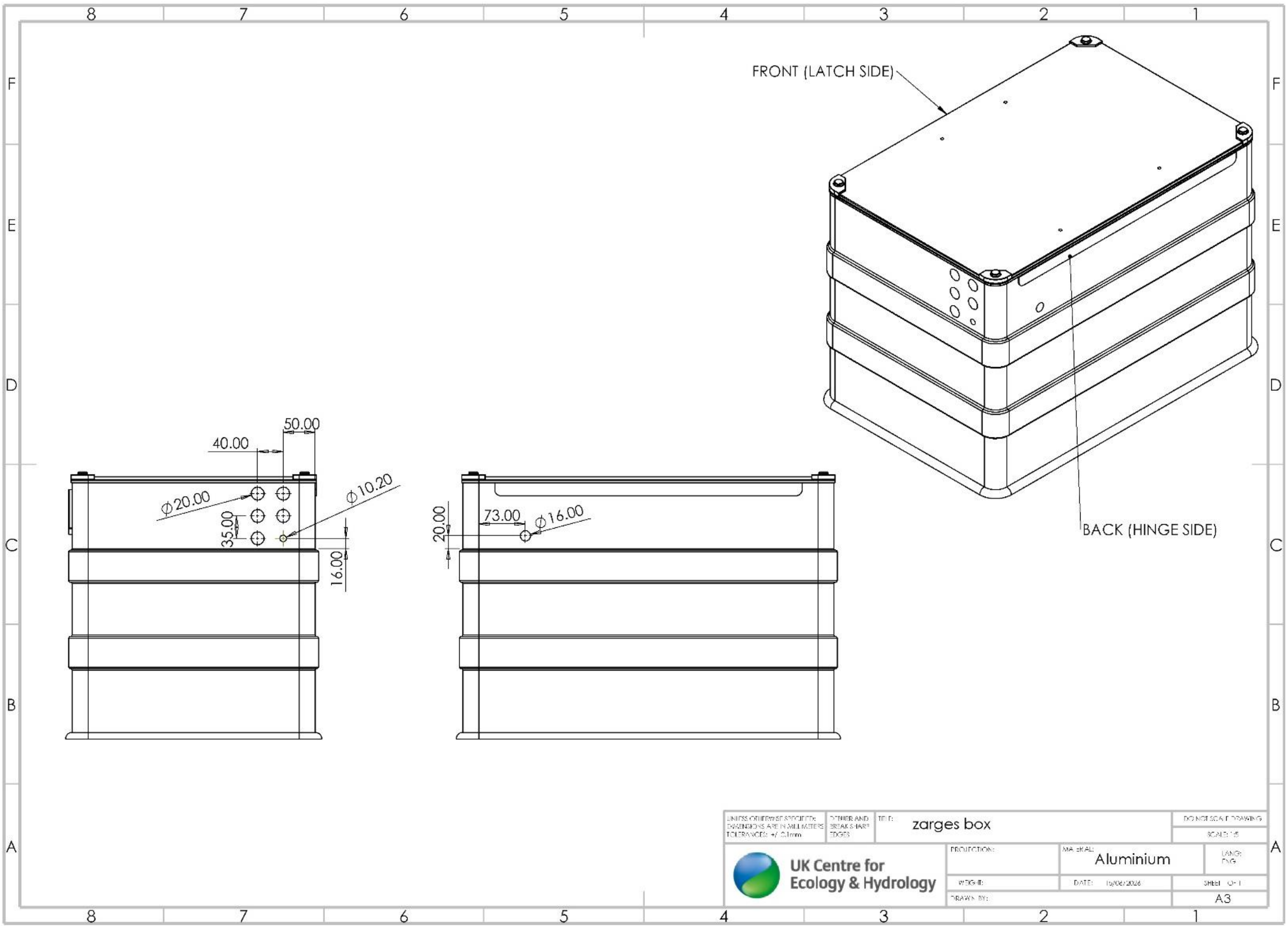
FRONT (LATCH SIDE)
BACK (HINGE SIDE)
50.00
40.00
Ø20.00
Ø10.20
35.00
16.00
20.00
73.00
Ø16.00
UNLESS OTHERWISE SPECIFIED: DIMENSIONS ARE IN MILLIMETERS TOLERANCES: +/- 0.1mm
DEBURR AND BREAK SHARP EDGES
TITLE:
zarges box
DO NOT SCALE DRAWING
SCALE: 1:5
UK Centre for Ecology & Hydrology
PROJECTION:
MATERIAL:
Aluminium
LANG: ENG
WEIGHT:
DATE:
SHEET 1 OF 1
DRAWN BY:
A3

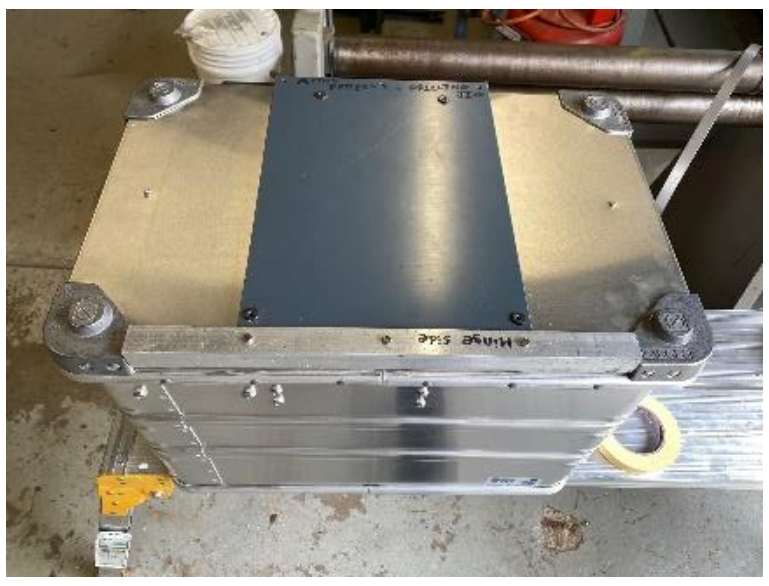

Use jig to drill the top

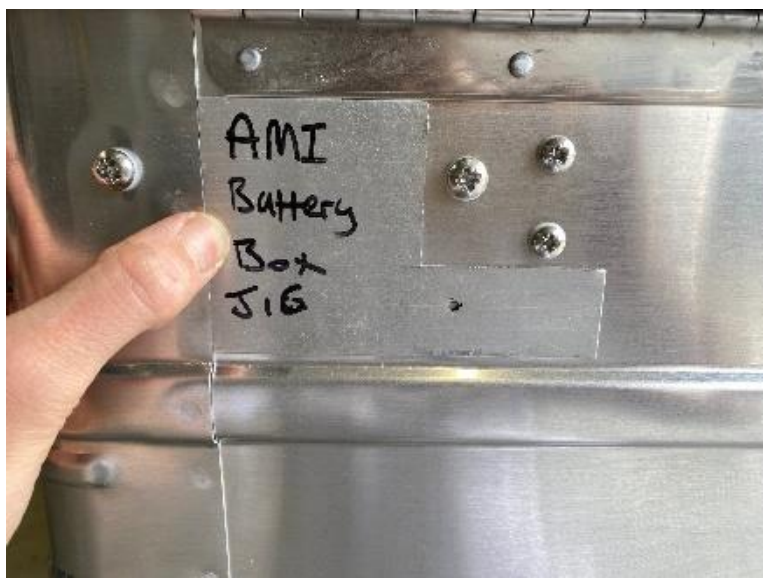

Mark back of box using jig and marker

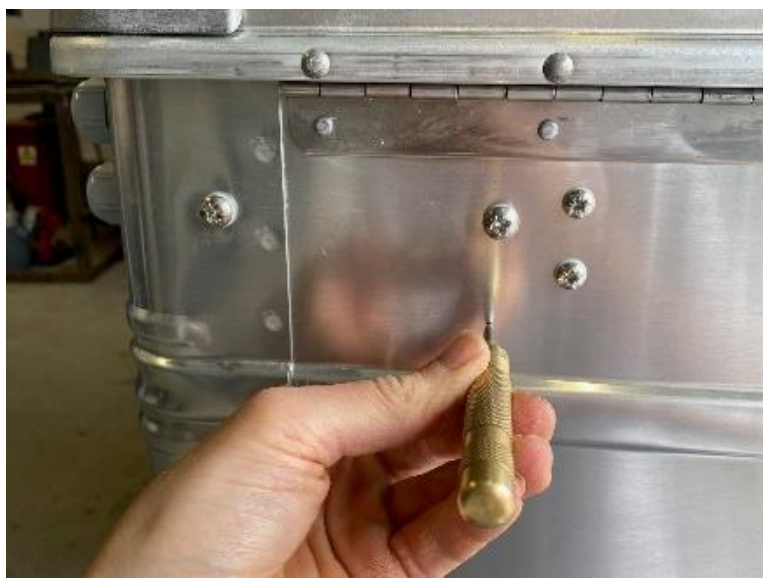

centre punch mark

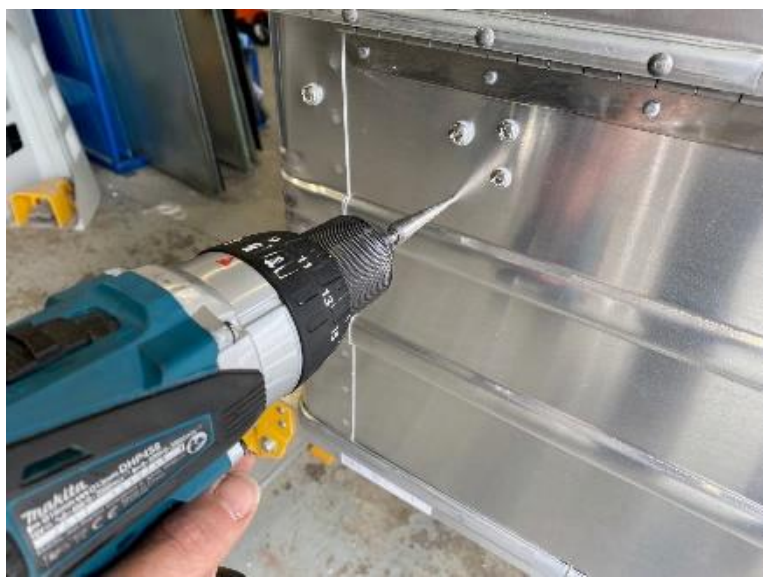

Drill out with step drill to 8mm

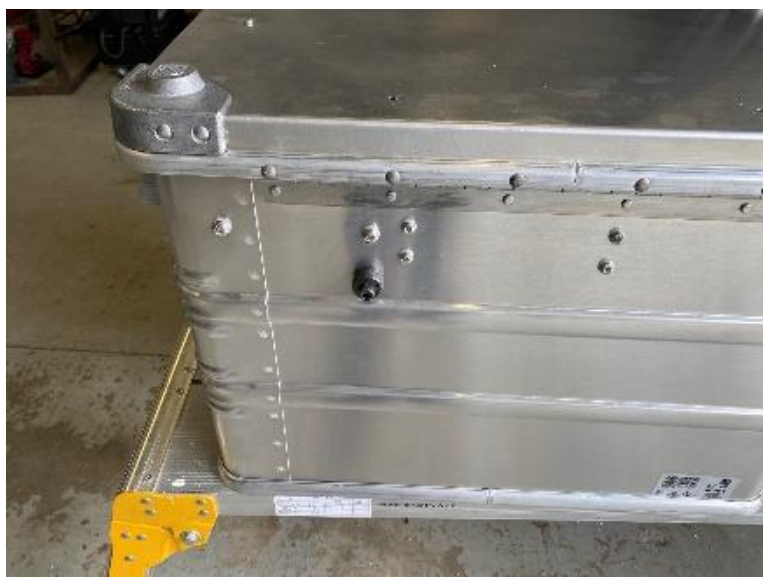

use q max cutter for making 16_{mm} hole

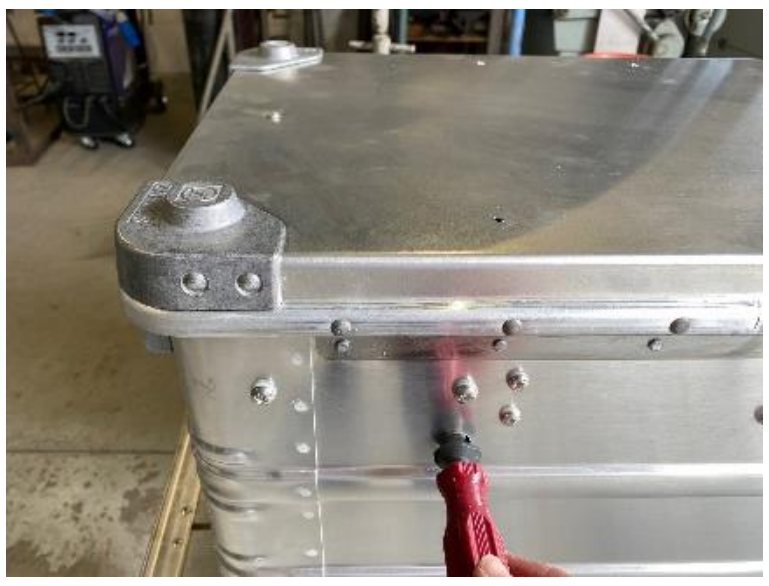

deburr all holes

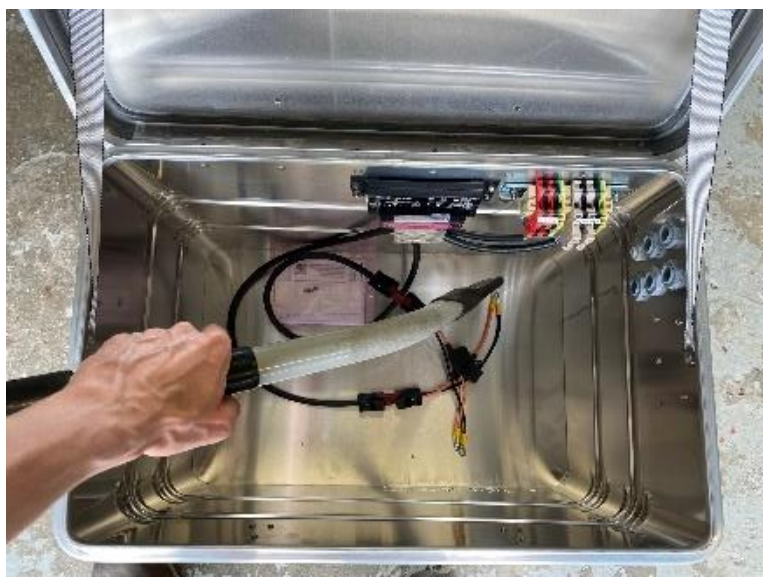

vacuume out any debri

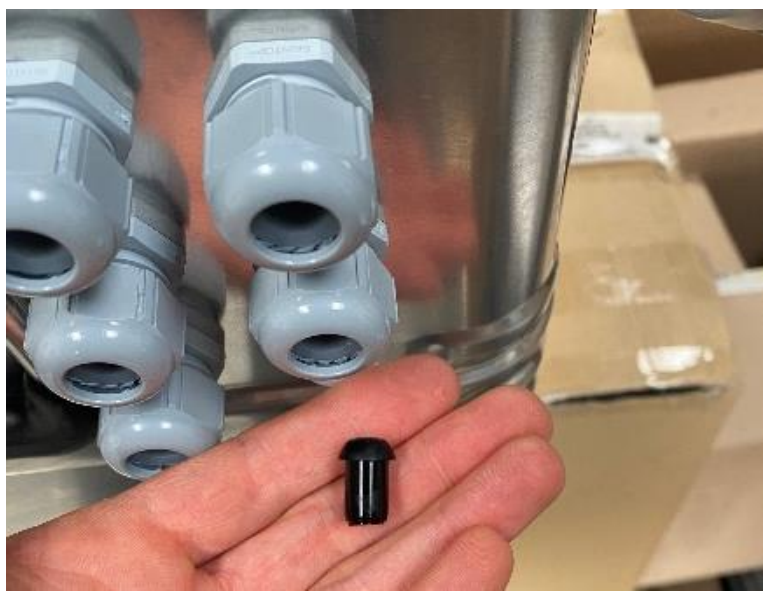

insert gland into lower right hole
leaving only the top two holes open

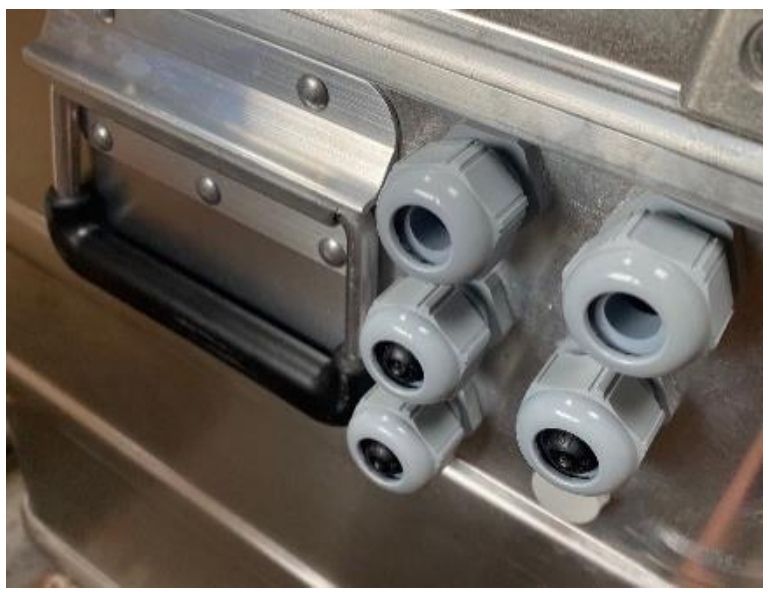

bottom 3 glands blanked top two open

Solar regulator

Mount the solar regulator inside the Zarges box on the hinge side. Then mount the din rail to the right hand side of the solar regulator and mount the terminal blocks and fuses (figure 4).

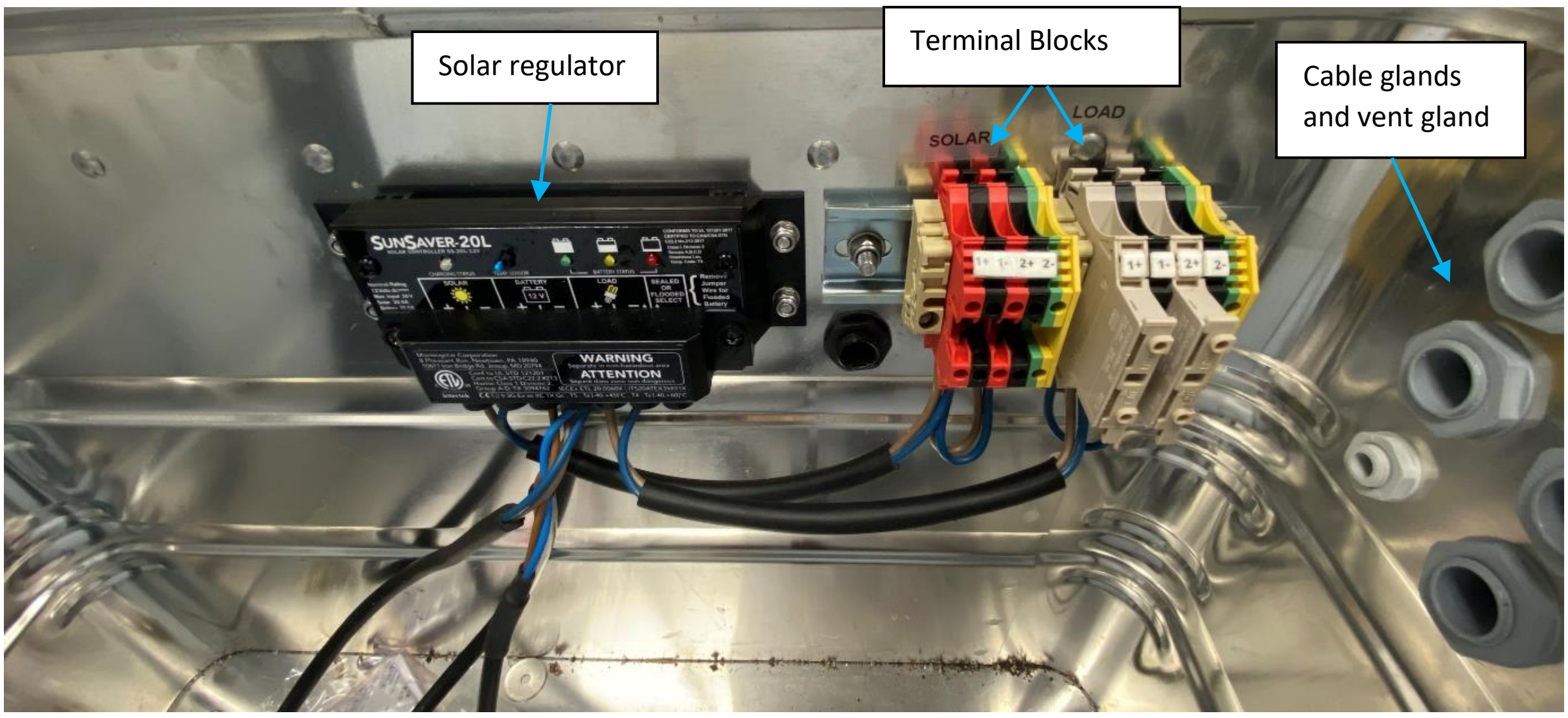

Figure 4: Positioning of the solar regulator and terminal blocks within the battery box

Finally wire up the batteries, solar regulator, solar panels and terminal blocks as shown in figure 5.

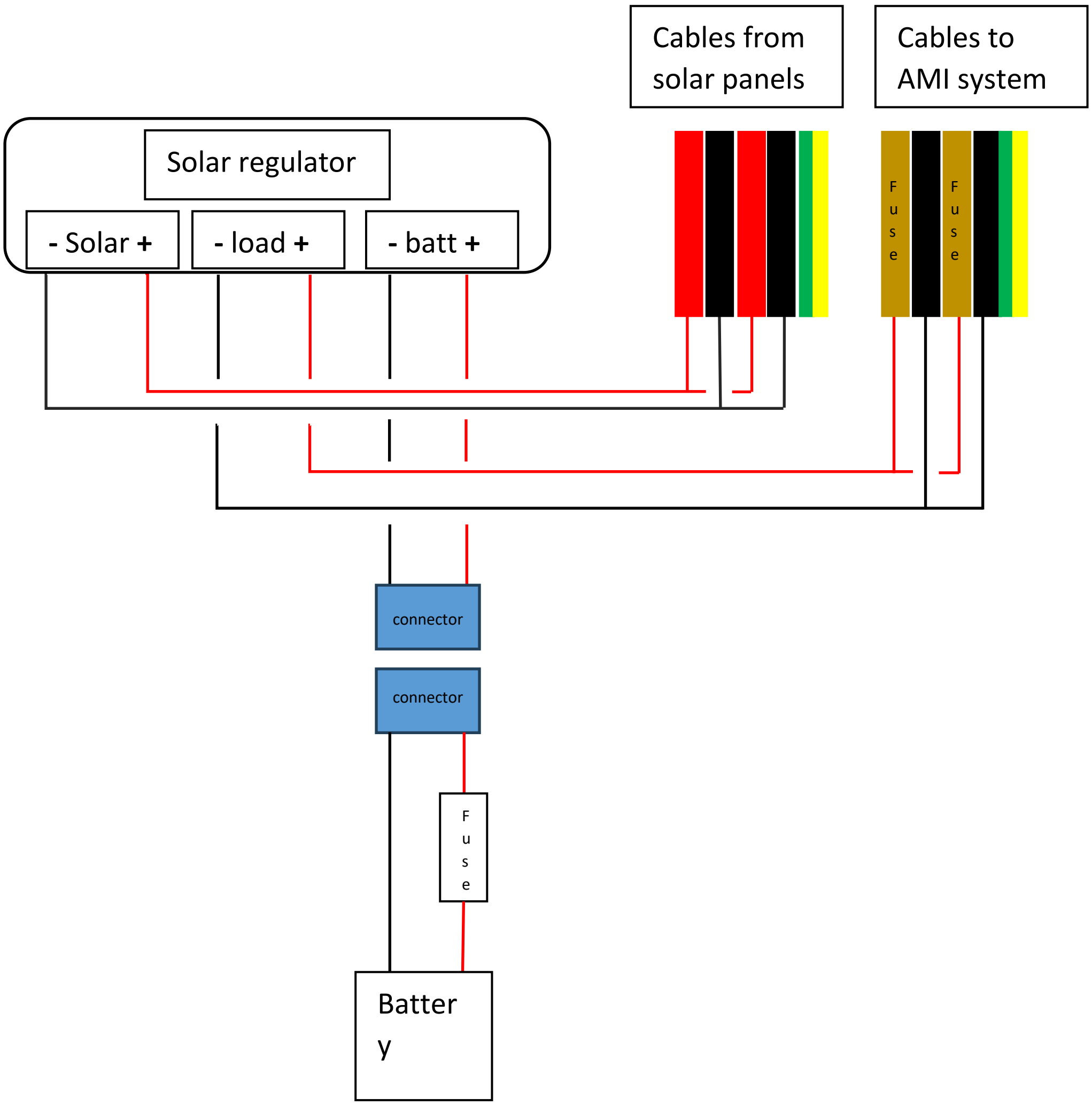

Figure 5 – Wiring diagram for the AMI battery box

Fixing kit

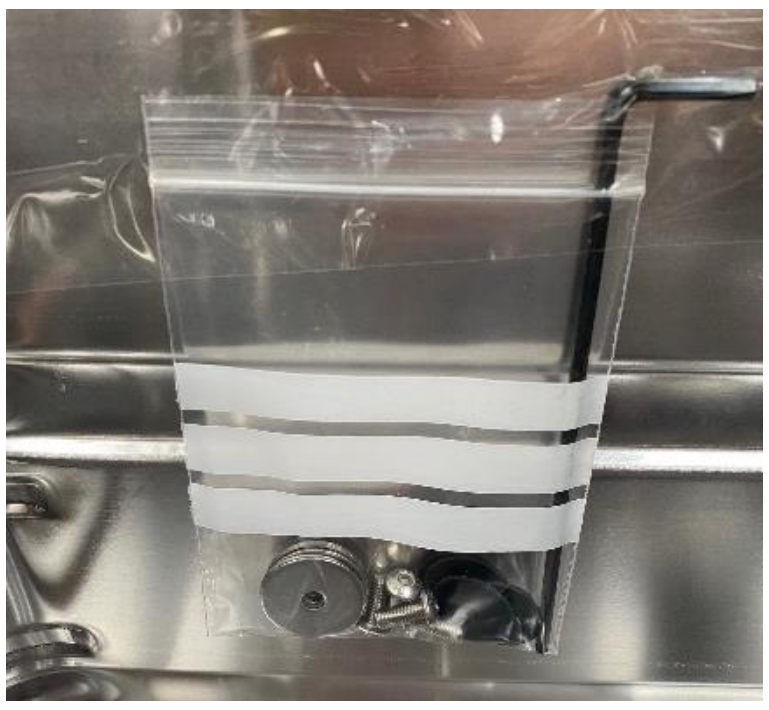

1 x 3 mm hex key
4 x M5 penny washers
4 x M5 button head screws
4 x rubber washer
1 x plastic bag

Other items

1 x back board
4 x 51mm foldback clip

Packing

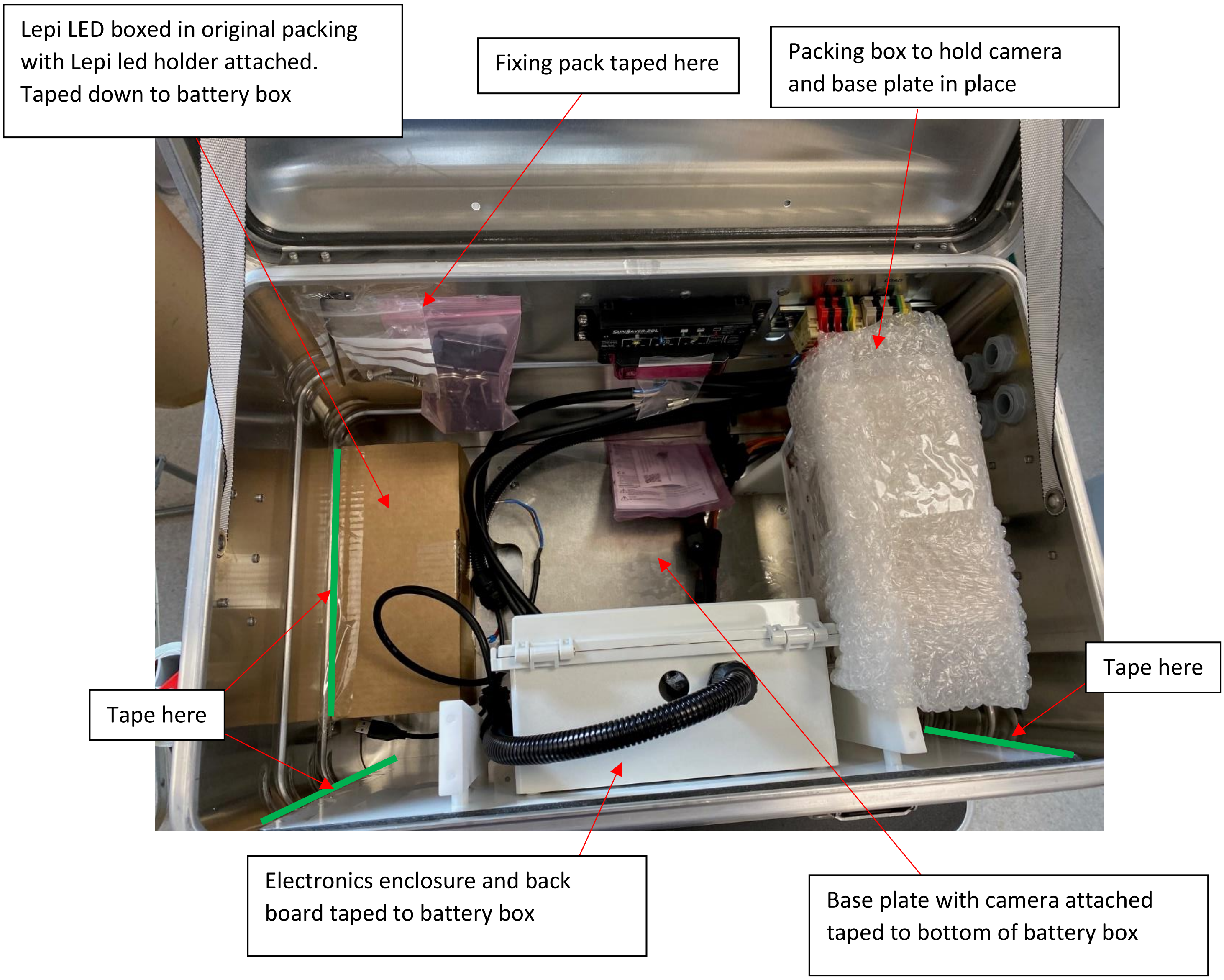

Supplement: Supplementary file 1 [file mmc3.pdf]
