# Supplementary material for: Automated monitoring of insects system: building a non-lethal and scalable solution for monitoring of nocturnal insects
Source: HardwareX. 2026 Aug 6;27:e00822. doi: 10.1016/j.ohx.2026.e00822 (PMC13499100; doi:10.1016/j.ohx.2026.e00822)
Supplement: Supplementary file 2 [file mmc1.docx]

All design files are available on Zenodo: <https://zenodo.org/records/20760744>
